# Identification of trait-associated microRNA modules in liver transcriptome of pig fed with PUFAs-enriched supplementary diet

**DOI:** 10.1007/s13353-024-00912-w

**Published:** 2024-11-15

**Authors:** C. S. Pareek, M. Sachajko, G. Kalra, S. Sultana, A. Szostak, K. Chalaskiewicz, K. Kepka-Borkowska, E. Poławska, M. Ogłuszka, D. Pierzchała, R. Starzyński, H. Taniguchi, E. Juszczuk-Kubiak, A. Lepczyński, B. Ślaska, W. Kozera, U. Czarnik, P. Wysocki, H. N. Kadarmideen, M. F. W. Te Pas, J. Szyda, M. Pierzchała

**Affiliations:** 1 Institute of Veterinary Medicine, Faculty of Biological and Veterinary Sciences, Nicolaus Copernicus University, 87-100 Toruń, Poland; 2 Division of Functional Genomics in Biological and Biomedical Research, Centre for Modern Interdisciplinary Technologies, Nicolaus Copernicus University, 87-100 Torun, Poland; 3 Department of Genomics and Biodiversity, Institute of Genetics and Animal Biotechnology of the Polish Academy of Sciences, Ul. Postepu 36A Str, 05-552 Jastrzebiec, Magdalenka Poland; 4 Maria Sklodowska-Curie National Research Institute of Oncology, W.K. Roentgena 5 Str, 02-781 Warsaw, Poland; 5 Department of Molecular Biology, Institute of Genetics and Animal Biotechnology of the Polish Academy of Sciences, Ul. Postepu 36A Str, 05-552 Jastrzebiec, Magdalenka Poland; 6 Department of Experimental Embryology, Institute of Genetics and Animal Biotechnology of the Polish Academy of Sciences, Ul. Postepu 36A Str, 05-552 Jastrzebiec, Magdalenka Poland; 7 African Genome Center, Mohammed VI Polytechnic University, UM6P, Lot 660, Hay Moulay Rachid Ben Guerir, 43150 Morocco; 8 Laboratory of Biotechnology and Molecular Engineering, Department of Microbiology Prof. Wacław, Dąbrowski Institute of Agriculture and Food Biotechnology – State Research Institute (IBPRS-PIB), Rakowiecka 36 Str, 02-532 Warsaw, Poland; 9 Department of Physiology, Cytobiology and Proteomics, West Pomeranian University of Technology, K. Janickiego 32 Str, 71-270 Szczecin, Poland; 10 Faculty of Animal Sciences and Bioeconomy, University of Life Sciences in Lublin, Akademicka 13 Str, 20-950 Lublin, Poland; 11 Department of Pig Breeding, Department of Animal Biochemistry and Biotechnology, Faculty of Animal Bio-Engineering, University of Warmia and Mazury in Olsztyn, Ul. M. Oczapowskiego 5 Str, 10-719 Olsztyn, Poland; 12 Department of Animal and Veterinary Sciences, Aarhus University, Blichers Alle 20, 8830 Tjele, Denmark; 13 Wageningen Livestock Research, Wageningen University and Research, 6708 WD Wageningen, The Netherlands; 14 Biostatistics Group, Department of Genetics, Wrocław University of Environmental and Life Sciences, Kozuchowska 7, 51-631 Wrocław, Poland

**Keywords:** Breed, Trait, Nutrigenomics, Gene expression profile, Bioinformatics

## Abstract

Dietary lipids provide energy, are cellular structural components, and are involved in physiological processes. Lipids are the dietary source in supplementary diet experiments in pigs. This study aims to investigate the dietary effects of PUFAs on the hepatic transcriptome and physiological pathways of two diets on two pig breeds. Polish Landrace (PL: *n* = 6) and six PLxDuroc (PLxD: *n* = 6) pigs were fed with a normal diet (*n* = 3) or PUFAs-enriched healthy diet (*n* = 3), and the hepatic miRNA profiles were studied for weighted gene co-expression network analysis biological interactions between gene networks and metabolic pathways of DE miRNA genes. The study identified trait-associated modules that were significantly associated with four phenotypic traits in the dietary groups of PL and PLxD: meat colour (a*), shoulder subcutaneous fat thickness, conductivity 24 h post-mortem (PE24), and ashes. Trait-wise, a large set of co-expressed miRNAs of porcine liver were identified in these trait-associated significant modules (9, 7, 2, and 8) in PL and PLxD. Each module is represented by a module eigengene (ME). Forty-four miRNAs out of 94 miRNAs interacted with 6719 statistically significant target genes with a target score > 90. The GO/pathway analysis showed association with pathways including regulation of metallopeptidase activity, sebaceous gland development, collagen fibril organization, WNT signalling, epithelial tube morphogenesis, etc. The study showed the differences in miRNA expression between the dietary groups of PL and PLxD breeds. Hub genes of discovered miRNA clusters can be considered predicted miRNA genes associated with PE24, meat colour, shoulder subcutaneous fat thickness, and ashes. Discovered target genes for miRNA clusters play significant roles in biological functions such as (i) muscle and body growth development, (ii) different cellular processes and developments, (iii) system development, and (iv) metabolic processes.

**Supplementary Information:**

The online version contains supplementary material available at 10.1007/s13353-024-00912-w.

## Introduction

Fatty acids (FAs) are the basic structure of lipids such as fats and phospholipids. Lipids are stored in adipose tissue as triacyl glycerides (Moghadasian and Shahidi 2017). Lipids are hydrophobic molecules and are an important source of metabolic energy. Lipids are the dietary source of FAs in a FA supplementary diet experiment in pigs. Lipids play a key role in the cell membrane structure and in the membrane permeability barrier by acting as a structural matrix (Elmadfa and Kornsteiner 2009; Moghadasian and Shahidi 2017). Fatty acids are involved in several biological functions, such as the transcriptional regulation of physiological processes (Eshak et al. 2017; Moghadasian and Shahidi 2017). Lipids delivered in diets contribute not only to energy supply, cell structure, and gene expression levels but they are involved in several physiological and biological processes associated with the health benefits in humans (health of meat consumers) and meat production in domestic animals (De Smet and Vossen 2016). Dietary lipids are comprised of fatty acids (FAs) and cholesterol. The FAs differ in the length of the carbon chains, which influences their physicochemical properties. Based on the presence or absence of double bonds between the carbon molecules, FAs are divided into three main classes: saturated FAs (SFAs: palmitic acid, myristic acid, and lauric acid), monounsaturated FAs (MUFAs: ω-9 oleic acid (OA), and polyunsaturated FAs (PUFAs) (Petrovic and Arsic 2016). The most common SFAs found in plant and animal tissues are those with a linear chain of 12 to 18 carbons, with palmitic acid (C16:0) being the most abundant and found in most plant oils, fish oil, and in the body fat of some animals. The most common SFAs in the diets are stearic acid (C18:0), myristic acid (C14:0), and lauric acid (C12:0) (Eshak et al. 2017; Moghadasian and Shahidi 2017). In humans, stearic acid is important and related to cholesterol levels. Stearic acid levels contribute to reducing the cholesterol levels in the human blood (Monsma and Ney 1993). The feed intake of myristic acid in the human diet improves long-chain n-3 levels, which contributes to improved cardiovascular health (Dabadie et al. 2005). A small amount of myristic acid, unlike palmitic acid, is rapidly metabolized in cultured rat hepatocytes (Rioux et al. 2000). The PUFAs have two or more double bonds in their aliphatic chain. There are two PUFA families which are non-interconvertible in mammals: the omega-3 (or n-3 or ω-3) and the omega-6 (or n-6 or ω-6) FAs. The essential ω-3 and ω-6 FA for mammals are linolenic acid (ALA; 18:3 ω-3) and linoleic acid (LA; 18:2 ω-6) which are precursors for longer PUFAs. Mammals cannot synthesize ALA and LA de novo due to a lack of specific desaturase enzymes and must be obtained them from food. Among ω-3 FAs, linolenic acid (ALA; 18:3 ω-3) is mostly contained in plants (i.e. flaxseed, canola, soybean, nuts, walnuts, chia seeds), while eicosapentaenoic acid (EPA; 20:5 ω-3) and docosahexaenoic acid (DHA; 22:6 ω-3) are present mostly in fish, seafood, and marine algae (Baker et al. 2016; Moghadasian and Shahidi 2017; Wood et al. 2008 Bork et al. 2020; Caterina 2011). Among ω-6 FAs, linoleic acid (LA; 18:2 ω-6) is provided by the seed oils, soybean, nuts, and cereals, while arachidonic acid (ARA; 20:4 ω-6) is found in poultry meat and eggs (Al-Khalaifah et al. 2020). The land-based food chain is dominated by a higher LA than ALA. These two essential FAs initiate ω-6 and ω-3 series, which have a different impact on the inflammatory response. Both SFA and PUFAs have been recognized as molecules regulating a variety of functions in the cell, such as serving as a source of energy, being a vital component of the cell membranes, and acting as signalling molecules, which regulate different processes, including gene expression. Despite being essential for maintaining homeostasis in animal organisms, a lack of enzymes responsible for endogenous synthesis of the omega-6 and omega-3 FAs by mammals necessitates their constant dietary intake. Hence, they are referred to as essential fatty acids (EFAs) (Simopoulos 2001; El-Badry et al. 2007). The importance of adding and enriching products with specific levels of healthier PUFAs diet is highly recognized by consumers. For example, the oils used in pig diets are palm oil, fish oil, sunflower oil, linseed oil, canola oil (Tognocchi et al. 2023; Souza et al. 2020), and soybean oil (Fanalli et al. 2022). The oil blends supplemented in the diets have been tested in experiments aimed to improve the performance, composition, and deposition of FAs, as well as carcass and meat quality characteristics, such as carcass yield, meat marbling, and enrichment of bacon and loin with omega-6 and stearic acid (Souza et al. 2020). For example, the inclusion of 3% soybean oil or canola oil in pig diets reduced loin shear force and increased oleic acid content in the intramuscular fat (Almeida et al. 2021). The experimental pig breed Landrace is a modern breed and one of the most widely distributed breeds in the world, which has high lean meat content and is known for producing high-quality pork (Vidal et al. 2005; Franco et al. 2014), whereas the Duroc (D) pig breed is characterized by its higher fat levels and is the predominate terminal sire used in the world; it excels for meat quality and eating characteristics, such as high percentage of IMF (marbling) and high pH value (Lonergan et al. 2001). The main aim of the present study was to investigate the effect of dietary omega-6 and omega-3 PUFAs on porcine miRNA gene expression in the liver transcriptome and to identify the physiological and molecular processes associated with co-expression network (WGCNA) at whole hepatic transcriptomic level in Polish Landrace (PL) and PLxD pigs. The porcine hepatic transcriptomes changed by omega-6 and omega-3 PUFAs dietary supplementation feeding were compared with standard diets. In this study, the correlation between co-expressed miRNAs and phenotypic traits was performed (i) to identify the miRNA expression profile and the targeted hub genes in trait-specific detected modules in PL and PLxD pigs, (ii) to identify the trait-associated miRNA genes of significance (GS) in PL and PLxD pigs, and (iii) to identify the hepatic miRNA target gene expression networks and metabolic pathways in trait-specific modules in PL and PLxD pigs.

## Materials and methods

### Animals

Twelve pigs representing PL (*n* = 6) and PLxD (*n* = 6) were investigated in a feed supplementation experiment as outlined earlier (Szostak et al. 2016, Ogłuszka et al. 2017).

### Experimental design

A WGCNA experiment was carried out to investigate the co-expression of porcine liver microRNA in PL and PLxD pigs. Based on the previous study (Szostak et al. 2016), we categorized two dietary groups: a standard (control) diet and a PUFAs-enriched diet to carry out the NGS-based miRNA-seq experiment. The PUFAs-enriched dietary group was classified as a PUFAs-enriched diet (LR, hereafter referred to as PUFAs-enriched diet) which was enriched both with LA and ALA including 660 mg of LA in 100 g of fodder and 64 mg ALA in 100 g of fodder. The control diet contained 268 mg of LA and 25 mg of ALA in 100 g of fodder. Both control and PUFAs-enriched diets were isoenergetic, with ME = 12.86 MJ/kg and ME = 13.51 MJ/kg of dry matter, respectively, and isoproteotic with crude protein percentages rate 15.66% and 15.65%. The WGCNA analysis was performed for the control vs. PUFAs-enriched dietary groups for each breed. The liver samples were investigated with miRNA-seq (*n* = 12) to compare hepatic transcriptomes characterized by low (control groups) with high (PUFAs-enriched diet groups) omega-6/omega-3 fatty acid ratio in both pig genotypes.

### Laboratory procedures of miRNA-seq experiment

Total RNA was extracted from 20 mg of liver samples (*n* = 12) of PL and PLxD pigs using RNeasy Lipid Tissue Mini Kit (Qiagen, Hilden, Germany) following the manufacturer’s instructions. The concentration and purity of RNA were measured using a NanoDrop spectrophotometer (Thermo Scientific, USA). RNA integrity number (RIN) was assessed by Bioanalyzer 2100 and RNA 6000 Nano kit (Agilent Technologies, Inc., Santa Clara, CA, USA). High-quality extracts (RIN ≥ 7) were used for miRNA library preparation. The construction of cDNA libraries was performed using Illumina TruSeq miRNA Sample Prep Kit v2 (Illumina, San Diego, CA, USA). The purification and size selection were performed using the Sage Pippin Prep 3% Cassettes Dye-Free (Sage Science, Inc., Beverly, MA, USA).

### Normalization and quality control (QC) of libraries

The library concentration to 2 nM was normalized using Tris–HCl 10 mM (pH 8.5). The QC procedures were performed using the 30 µl Qiagen EB and 1 µl of prepared library sample on Agilent Bioanalyzer (1000 DNA chip). After the validation of libraries, KAPA quantification with samples in the TSP1 plate was performed. The denaturation of libraries and the MiSeq NGS sequencing were performed according to manufacture guidelines and protocols.

### Pre- and post-processing of the miRNA-seq data (miRNA mapping and reads counting)

The FastQC software was used for quality control of 12 FastQ files with porcine miRNA reads (Brown et al. 2017). Good quality of the reads allowed to skip the trimming head or trail end of reads with QS < 10. The next step was to map obtained reads to the reference genome. For a reference genome, hairpin and mature sequences of porcine were used. The sequences were extracted from miRBase v.22 (Grifith-Jones et al. 2007; Kozomara and Grifith-Jones 2010). To map the reads from FastQ file mapper.pl, a module of miRDeep2 was used (Friedlander et al. 2012). Mapping reads were performed for all 12 FastQ files. The parameter used was *-l 18* which defines the seed region of a read to the first 18 bases in the sequence. The output of this step was the Fasta file with mapped reads. The second feature of miRDeep2 software is the quantifier module. The module maps the sequences to the predefined hairpin and mature forms of miRNA and determines the number of read counts. A read is assumed to represent a sequenced mature miRNA if it falls within the same position on the precursor, plus 2 nt upstream and 5 nt downstream (Friedlander et al. 2012).

### Bioinformatic analysis of miRNA-seq data of the porcine liver transcriptome

After sequencing, 12 Fasta files were obtained, one for every animal. The variance stabilization counts were used to normalize counts for WGCNA analysis (Langfelder and Horvath 2008). For all samples from four experimental groups, one co-expression network was created. Breed and diet information were added to the sample’s phenotypic traits to find the potential correlation between detected modules and breed, diet type, or experimental group. The matrix of raw counts of miRNAs per sample was used as an input for the WGCNA analysis. MiRNAs were placed in rows and samples in columns. Initially, the matrix contained counts for 535 distinct miRNAs for 12 samples. After filtering, 226 miRNAs were normalized with the varianceStabilizingTransformation function from the R DESeq2 library (Anders and Huber 2010; Langfelder and Horvath 2008). Before network construction, the miRNA expression profile was clustered and visualized as a dendrogram. Euclidian distance was used to show the difference between samples. Furthermore, the heatmap for phenotype was generated to compare the quantitative traits between the samples (Horvath and Dong 2008). Later, the soft-thresholding power β was established (Zhang and Horvath 2005). The co-expression network was constructed using the normalized miRNA counts and discovered earlier soft-thresholding power β (Horvath et al. 2006). The co-expression network identified correlated miRNA and clustered them into modules. Each module is represented by a module eigengene (ME), which is the first principal component of the expression values of all genes in the module (Yip et al. 2007). Each module was correlated with the phenotypic trait. Modules specific to the trait were chosen by the *p*-value (< 0.05) (Langfelder and Horvath 2008).

#### Raw expression data preparation and normalization

The WGCNA method is sensitive for the samples with low count numbers which generates statistical noise giving false correlation based on zeros (source: WGCNA package documentation). If the miRNA had counts equal to 0 in ≥ 6 samples, it was removed from the analysis. A total of 94 miRNAs were normalized using the varianceStabilizingTransformation function from the DESeq2 library. Argument blinds were set to *True*, and fit type was set to *local* to obtain the most robust normalization (Anders and Huber 2010).

#### Phenotypic trait description

A total of 34 phenotypic traits were investigated and correlated with identified modules. The investigated traits were related to the body growth, meat colour, components of meat, the content of PUFA acids, and other general meat parameters (Table S1).

#### Finding target genes

The miRDB, an online database with miRNA target predictions, was used to find the target genes. The database contains information about the target genes and their target genes’ score. Target gene score is the parameter describing how much a miRNA is related to the gene. The higher score informs about a stronger relationship between miRNA to a target gene (Liu and Wang 2019). The threshold was set at a score of 90, and all target genes below were removed from further analysis.

### Pathway analysis

All target genes clustered into the modules were included in pathway analysis performed with Cytoscape ClueGo v2.5.9 software (Bindea et al. 2009). The GO/pathway analysis was performed using the GO-BiologicalProcess-EBI-UniProt-GOA database for human genes (Barrel et al. 2009). The statistically adjusted *p*-value threshold as Bonferroni correction was set at *p* < 0.05. Pathways and functional groups were generated for each module of miRNAs’ target genes.

## Results

### Pre- and post-processing of the miRNA-seq data

The MiSeq FastQ miRNA-seq data of both breeds of the pig were submitted to the NCBI SRA database (https://www.ncbi.nlm.nih.gov/sra, Accession: SRX21460914 to SRX21460925) with bio-project accession number PRJNA1008399. Twelve FastQC files obtained during the NGS all samples had score > 20 in Phred-33. The sample with the lowest number of total sequences was 3R1.FastQ with 135,842 total sequences. The largest number of total sequences was present in 1R2.FastQ, which had 4,018,483 total sequences. The lowest GC content was found in the 21R2.FastQ sample with 46%. The highest content of GC bases was 49% in samples 1R2.FastQ, 4R3.FastQ, and 9R3.FastQ (Table 1).

**Table 1LE: Please check if the table captions, table entries, table note, and other relevant details in Tables 1 and 2 are presented correctly. Otherwise, please amend.** Base statistics of FastQC quality control analysis

Filename^1^	Sample^1^	Diet^1^	Breed^1^	Total sequences^1^	Sequence length^1^	GC content (%)^1^
1R1.FastQ.gz	1R1_PLxD_ctrl	C	PLxD	1,990,353	35–51	47
1R2.FastQ.gz	1R2_PLxD_ctrl	C	PLxD	4,018,483	35–51	49
3R2.FastQ.gz	3R2_PLxD_ctrl	C	PLxD	1,703,333	35–51	47
2R1.FastQ.gz	2R1_PLxD_treat	T	PLxD	315,868	35–51	47
4R1.FastQ.gz	4R1_PLxD_treat	T	PLxD	3,410,675	35–51	47
4R3.FastQ.gz	4R3_PLxD_treat	T	PLxD	826,995	35–51	49
3R1.FastQ.gz	3R1_PL_ctrl	C	PL	135,842	35–51	47
30R2.FastQ.gz	30R2_PL_ctrl	C	PL	194,906	35–51	47
32R3.FastQ.gz	32R3_PL_ctrl	C	PL	1,335,600	35–51	47
9R3.FastQ.gz	9R3_PL_treat	T	PL	1,383,595	35–51	49
20R3.FastQ.gz	20R3_PL_treat	T	PL	1,949,792	35–51	48
21R2.FastQ.gz	21R2_PL_treat	T	PL	541,104	35–51	46

^1^Filename: name of the FastQ file input. Sample: sample id. Diet: animal diet. *C* standard diet, *T* treated group, fed with a diet enriched with PUFA. Breed: breed of the animal. *PL* Polish Landrace, *PLxD* Polish Landrace Duroc crossbreed. Total sequences: number of reads in file. Sequence length: length of reads present in the sample. GC content (%): amount of G and C bases in the reads in percent

### Identification of trait-specific co-expressed miRNA target genes in PL and PLxD pigs

#### Clustering dendrogram and heatmap of the investigated phenotypic trait associated with each pig

**Fig. 1:**
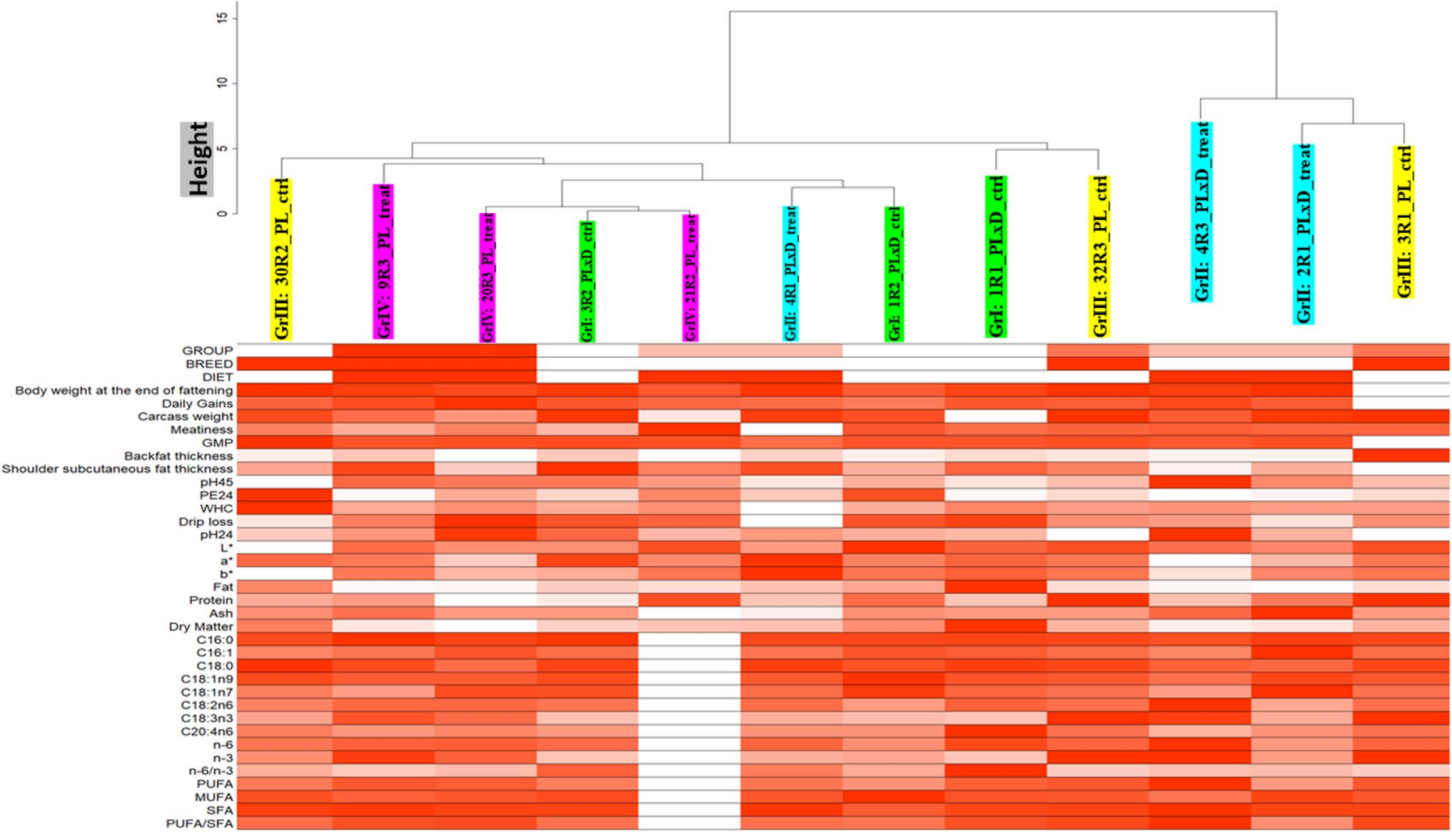
Clustering dendrogram of samples based on their Euclidean distance and heatmap of the investigated phenotypic trait associated with each pig. The more intense the colour, the higher the value of the phenotypic trait. White colour represents samples with no phenotypic records, because of missing phenotypic records. The heatmap displays the phenotypic traits (*y*-axis) of investigated PL and PLxD pigs (*x*-axis). On top, clustering dendrogram denotes the sample ID of PLxD pigs (group I (1R1_PLxD_ctrl, 1R2_PLxD_ctrl, and 3R2_PLxD_ctrl), group II (2R1_PLxD_treat, 4R1_PLxD_treat, and 4R3_PLxD_treat)) and the sample ID of PL pigs (group III (3R1_PL_ctrl, 30R2_PL_ctrl, 32R3_PL_ctrl), group IV (9R3_PL_treat, 20R3_PL_treat, and 21R2_PL_treat)), respectively

Before network construction and module detection, 12 liver transcriptomes from PLxD (group I (control: with standard diet) and group II (treated with PUFAs-enriched diet including standard diet + linseed oil + rapeseed oil diets)) and PL (group III (control: with standard diet) and group IV (treated with PUFAs-enriched diet including standard diet + linseed oil + rapeseed oil diets)) were clustered and visualized in a heatmap to define how the body growth related (BW) trait (Table S1) correlated to the sample dendrogram (Fig. 1).

#### Selecting and choosing the soft-thresholding power (analysis of network topology)

**Fig. 2:**
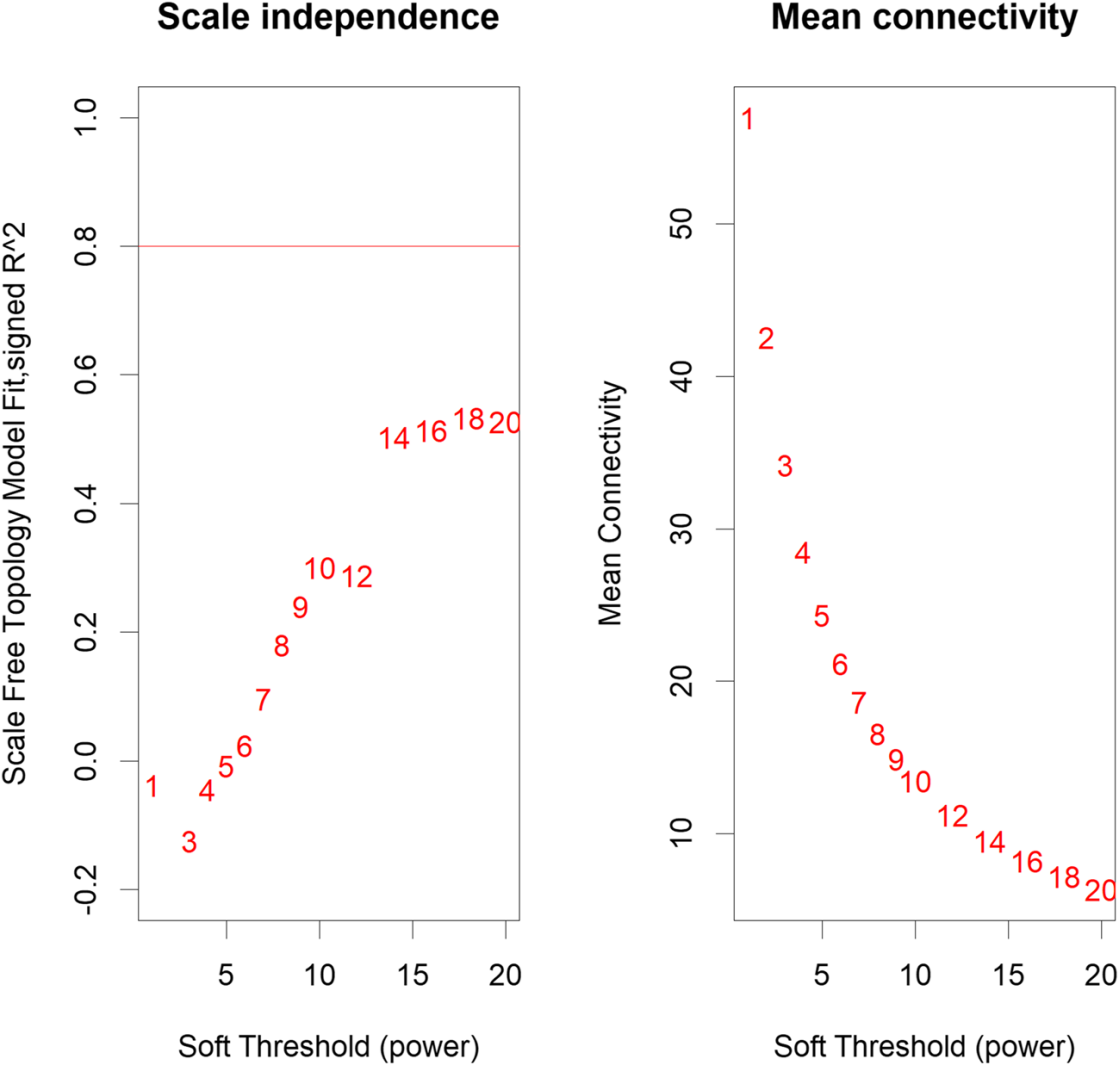
Determination of network topology for various soft-thresholding powers in the porcine liver transcriptome using WGCNA. The left panel plot displays the scale-free topology fit index (*y*-axis) as a function of the various soft-thresholding powers (β) (*x*-axis). The right panel plot displays the mean connectivity (degree, *y*-axis) as a function of the various soft-thresholding powers (*x*-axis)

The first step of the WGCNA co-expression analysis was to establish the soft threshold power β. Since it was impossible to reach R2 ≥ 0.8 (Fig. 2), soft-thresholding power β was set for 18 as suggested in the WGCNA documentation.

#### Co-expression network construction and module detection

**Fig. 3:**
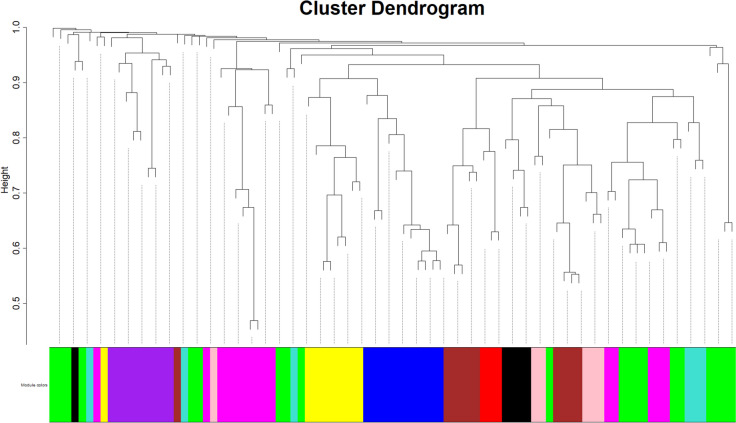
miRNA clustering dendrogram and module of co-expression genes, with dissimilarity based on the topological overlap, together with assigned module for porcine liver transcriptome. Correlated miRNA was grouped into modules identified through the Dynamic Tree Cut function. Each leaf, which is a short vertical line, corresponds to a specific miRNA. Branches of the dendrogram group together densely interconnected highly co-expressed miRNAs

The block-wise modules function from the WGCNA library was used to create the gene network. The hierarchical agglomerative clustering was constructed considering the 1-TOM as a distance to identify the groups of trait-associated co-expressed miRNAs. This cut-off was chosen considering the miRNA transcriptome’s small size and the fact that a single miRNA can target multiple RNA transcripts. With this procedure, 10 different modules of eigengene RNA were identified (Fig. 3). The size of each miRNA module was green (20), magenta (15), blue (11), brown (10), purple (9), yellow (9), pink (6), turquoise (6), black (5), and red (3), respectively.

#### Correlation between modules and investigated phenotypic trait and quantification of the trait-associated modules

**Fig. 4:**
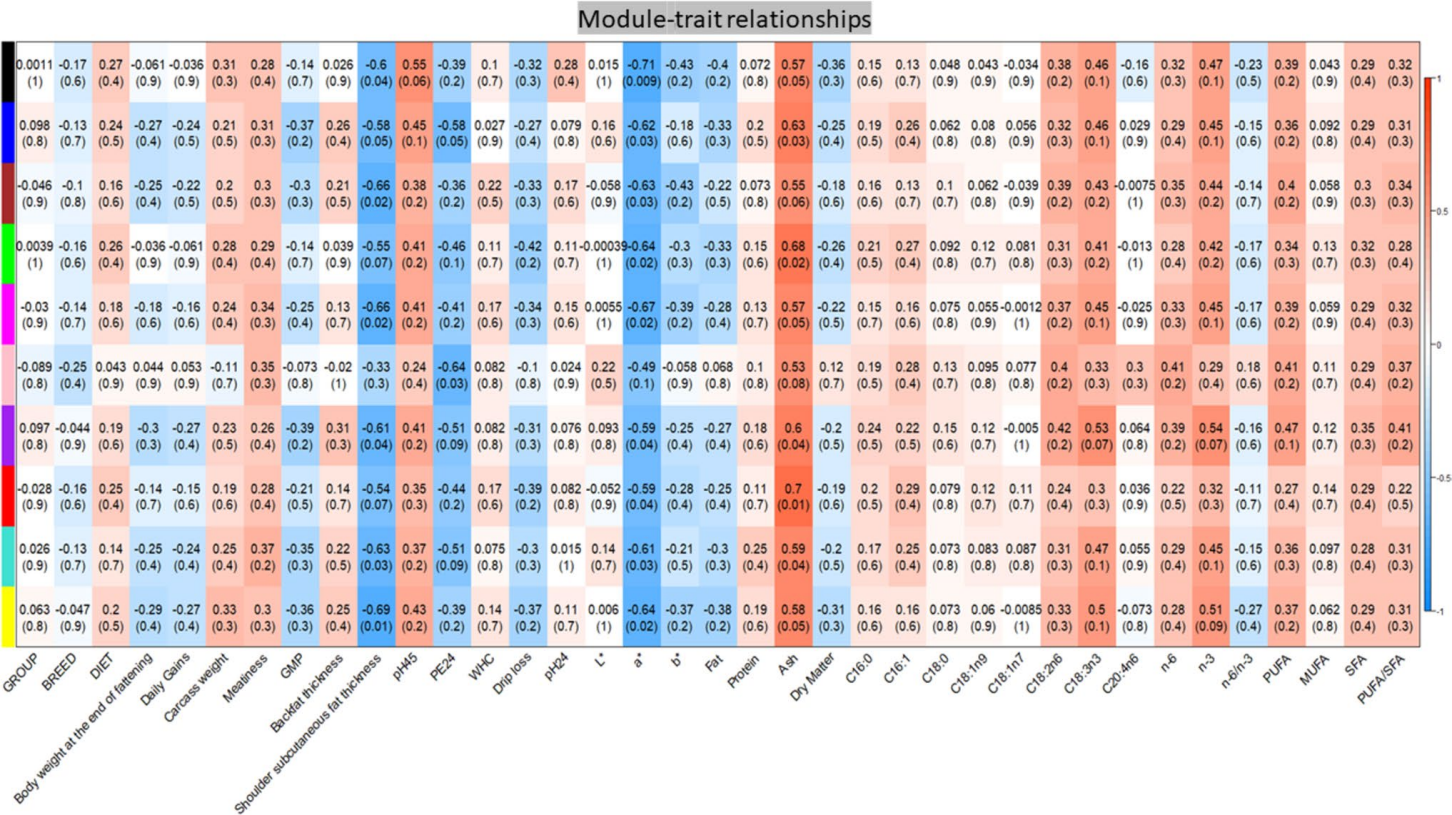
Heatmap of module-trait correlations: the correlations of identified clusters and phenotypic trait. Each row in the table corresponds to a module and each column to a phenotypic trait. Numbers in the table report correlation scores between modules and traits, with the *p*-values of the correlations in parentheses. The table is colour-coded by correlation according to the colour legend on the right. Blue indicates a negative correlation while red indicates a positive one

A total of 37 porcine phenotypic traits (Table S1) were investigated in the WGCNA analysis. The absolute value of the correlation of paired miRNAs was used to define the trait-associated gene co-expression network. In this analysis, the trait-associated modules that are significantly associated with the measured phenotypic traits were identified. We correlated eigengenes with external traits and selected the most significant associations. To deal with missing data, the pairwise deletion was applied. The resulting colour-coded table representing the trait-associated MEMs is shown in Fig. 4. The analysis identifies the several significant trait-associated modules, with weighted values of the investigated porcine phenotypic trait.

Modules with *p*-value ≤ 0.05 were classified as statistically significant for the trait, and four traits were identified with significant modules. Shoulder subcutaneous fat thickness is in correlation with seven modules (black, blue, brown, magenta, purple, turquoise, and yellow). The strongest correlation for this trait was present with the yellow module (corr =  − 0.69, *p*-value = 0.01). The a* trait was correlated with nine modules (black, blue, brown, green, magenta, purple, red, turquoise, and yellow). The strongest correlation was observed with the black module (corr =  − 0.71, *p*-value = 0.009). Ash was positively correlated with eight modules (black, blue, green, magenta, purple, red, turquoise, and yellow), where the strongest correlation was with the red module (corr = 0.7, *p*-value = 0.01). PE24 (conductivity 24 h post-mortem) correlated with two modules (blue and pink). The strongest correlation was with the pink module (corr =  − 0.64, *p*-value = 0.03).

#### Expression profile of detected modules

**Fig. 5:**
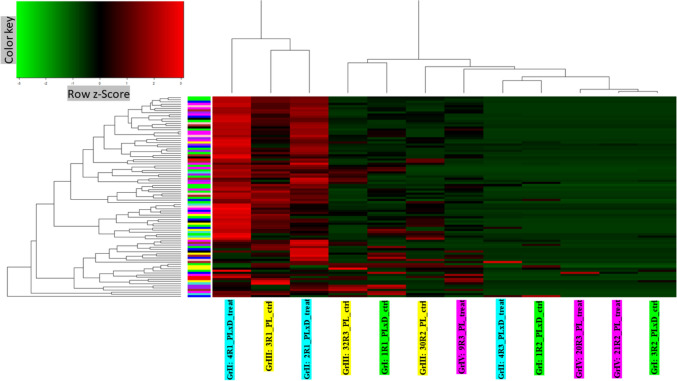
Expression heatmap of all detected modules. The greener colour indicates a lower expression while the redder colour indicates a higher expression. Columns represent samples. The dendrogram represents the Euclidean distance between rows and columns. On top of the dendrogram is the miRNA expression profile. On the bottom sample, IDs of PLxD pigs (group I (1R1_PLxD_ctrl, 1R2_PLxD_ctrl, and 3R2_PLxD_ctrl), group II (2R1_PLxD_treat, 4R1_PLxD_treat, and 4R3_PLxD_treat)) and the sample ID of PL pigs (group III (3R1_PL_ctrl, 30R2_PL_ctrl, 32R3_PL_ctrl), group IV (9R3_PL_treat, 20R3_PL_treat, and 21R2_PL_treat)), respectively. Rows represent miRNAs. On the left of the dendrogram is the Euclidean distance profile of miRNA expression. The colour symbolizes the module membership of a miRNA

A more detailed insight into the miRNAs clustered into the trait-correlated modules was obtained with an expression heatmap of miRNAs in specific clusters. The heatmaps were generated for all modules grouped, for every trait with its significantly correlated modules grouped, and for every module separately. Expression data were centred and scaled by the rows which represent the miRNA and columns represent the sample ID. Furthermore, the dendrogram to represent the distance was generated and is visible in the heatmap. The dendrogram represents the Euclidean distance between rows and columns. The heatmap with all detected modules (Fig. 5) shows a close distance between samples 4R3_PLxD_treat (group II), 2R1_PLxD_treat (group II), and 3R1_PL_ctrl (group III) with relatively high expression of miRNA in the modules. Samples 4R1_PLxD_ctrl (group I), 1R2_PLxD_ctrl (group I), 20R3_PL_treat (group IV), 21R2_PLxD_treat (group II), and 3R2_PLxD_ctrl (group I) characterize the relatively low expression of miRNA in all modules. A small distance was also observed between 32R3_PL_ctrl (group III), 1R1_PLxD_ctrl (group I), 30R2_PLxD_ctrl (group I), and 9R3_PL_treat (group IV) with mixed, highly and lowly expressed miRNAs.

Shoulder subcutaneous fat thickness (Fig. S1), a* (Fig. S2), and ash (Fig. S3) traits have similar Euclidean distances between samples. The sample distance based on the expression of genes from modules correlated with the PE24 trait (Fig. S4) differs from the dendrogram of Fig. 5. A higher distance of the 3R1_PL_ctrl sample from the 4R3_PLxD_treat and 2R1_PLxD_treat samples were observed. The rest of the samples were characterized with a similar distance compared to the distance calculated for all the miRNAs in eight modules (Fig. S5 to Fig. S12).

#### Identification of trait-associated miRNA genes of significance (GS) in PL and PLxD pigs

Intra-modular analysis for MEM (module membership of miRNA) was carried out to identify the correlation between the miRNA and the investigated porcine phenotypic trait. Four phenotypic traits in PL and PLxD pigs were identified, viz. a*, shoulder subcutaneous fat thickness, conductivity 24 h post-mortem (PE24), and ashes, respectively. Trait-wise, a total of 9, 7, 2, and 8 trait-associated significant modules in PL and PLxD pigs were identified. A detailed description is described in supplementary Figures S13-S38.

#### Mapping of the fixed target genes of miRNA pig

The list of target genes for porcine miRNA was downloaded from the miRDB.org database (https://mirdb.org/). The target genes with a target score greater than 90 were classified as significant. A total of 94 miRNA with the module membership was established during WGCNA analysis. The obtained results revealed that a total of 44 miRNAs (out of 94 miRNA) had 6719 statistically significant target genes with the target score > 90 (Table S2). The highest number of target genes was found for ssc-miR-30e-5p (520) which is a member of the magenta module. The second highest number of target genes was found for the ssc-miR-30b-5p and ssc-miR-30c-5p (518 each) included in the modules pink and yellow, respectively. The lowest number of target genes was found for ssc-miR-126-3p and ssc-miR-423-3p (1 each) included in the modules purple and brown, respectively (Table S2). Target genes were grouped by the module of its miRNA target. miRNAs from the yellow module had the highest number of target genes (1308); the brown module miRNAs identified 1114 genes, and the green and magenta module miRNAs targeted 963 genes, respectively. The lowest number of target genes was present in the red module (*n* = 27) (Table S3).

### Biological gene networks and pathway analysis of trait-specific co-expressed miRNA target genes in PL and PLxD pigs

#### Identification of miRNAs’ target genes representing biological gene networks and GO/pathways for the trait-specific black module in PL and PLxD pigs

The ClueGO analysis identified 38 GO/pathway-specific terms associated with the black module miRNAs’ target genes in PL and PLxD pigs (Fig. S39). Furthermore, the ClueGO analysis identified 14 functional groups for the trait-associated black module miRNAs’ target genes in PL and PLxD pigs (Fig. S40). Finally, the distribution of all 38 module-specific GO/pathways-specific terms, represented in the 14 functional group networks, is visualized in supplementary Figures S39-S41.

#### Identification of miRNAs’ target genes representing biological gene networks and GO/pathways for the trait-specific blue module in PL and PLxD pigs

The ClueGO analysis identified 57 GO/pathway-specific terms associated with the blue module miRNAs’ target genes in PL and PLxD pigs (Fig. S42). Furthermore, the ClueGO analysis identified 8 functional groups for the trait-associated blue module miRNAs’ target genes in PL and PLxD pigs (Fig. S42-S44). Finally, the distribution of all 57 module-specific GO/pathways-specific terms, represented in the 8 functional group networks, is visualized in supplementary Fig. S44.

#### Identification of miRNAs’ target genes representing biological gene networks and GO/pathways for the trait-specific brown module in PL and PLxD pigs

The ClueGO analysis identified 88 GO/pathway-specific terms associated with the brown module miRNAs’ target genes in PL and PLxD pigs (Fig. S45). Furthermore, the ClueGO analysis identified 6 functional groups for the trait-associated brown module miRNAs’ target genes in PL and PLxD pigs (Fig. S46). Finally, the distribution of all 88 module-specific GO/pathways-specific terms, represented in the 6 functional group networks, is visualized in supplementary Figures S45-S47.

#### Identification of miRNAs’ target genes representing biological gene networks and GO/pathways for the trait-specific green module in PL and PLxD

The ClueGO analysis identified 90 GO/pathway-specific terms associated with the green module miRNAs’ target genes in PL and PLxD pigs (Fig. S48). Furthermore, the ClueGO analysis identified 5 functional groups for the trait-associated green module miRNAs’ target genes in PL and PLxD pigs (Fig. S49). Finally, the distribution of all 90 module-specific GO/pathways-specific terms, represented in the 5 functional group networks, is visualized in supplementary Figures S48-S50.

#### Identification of miRNAs’ target genes representing biological gene networks and GO/pathways for the trait-specific magenta module in PL and PLxD pigs

The ClueGO analysis identified 57 GO/pathway-specific terms associated with the magenta module miRNAs’ target genes in PL and PLxD pigs (Fig. S51). Furthermore, the ClueGO analysis identified 4 functional groups for the trait-associated magenta module miRNAs’ target genes in PL and PLxD pigs (Fig. S52). Finally, the distribution of all 57 module-specific GO/pathways-specific terms, represented in the 4 functional group networks, is visualized in supplementary Figures S51-S53.

#### Identification of miRNAs’ target genes representing biological gene networks and GO/pathways for the trait-specific pink module in PL and PLxD pigs

The ClueGO analysis identified 54 GO/pathway-specific terms associated with the pink module miRNAs’ target genes in PL and PLxD pigs (Fig. S54). Furthermore, the ClueGO analysis identified 9 functional groups for the trait-associated pink module miRNAs’ target genes in PL and PLxD pigs (Fig. S55). Finally, the distribution of all 54 module-specific GO/pathways-specific terms, represented in the 9 functional group networks, is visualized in supplementary Figures S54-S56.

#### Identification of miRNAs’ target genes representing biological gene networks and GO/pathways for the trait-specific purple module in PL and PLxD pigs

The ClueGO analysis identified 16 GO/pathway-specific terms associated with the purple module miRNAs’ target genes in PL and PLxD pigs (Fig. S57). Furthermore, the ClueGO analysis identified 9 functional groups for the trait-associated purple module miRNAs’ target genes in PL and PLxD pigs (Fig. S58). Finally, the distribution of all 16 module-specific GO/pathways-specific terms, represented in the 9 functional group networks, is visualized in supplementary Figures S57-S59.

#### Identification of miRNAs’ target genes representing biological gene networks and GO/pathways for the trait-specific turquoise module in PL and PLxD pigs

The ClueGO analysis identified 14 GO/pathway-specific terms associated with the turquoise module miRNAs’ target genes in PL and PLxD pigs (Fig. S60). Furthermore, the ClueGO analysis identified 7 functional groups for the trait-associated turquoise module miRNAs’ target genes in PL and PLxD pigs (Fig. S61). Finally, the distribution of all 14 module-specific GO/pathways-specific terms, represented in the 7 functional group networks, is visualized in supplementary Figures S60-S62.

#### Identification of miRNAs’ target genes representing biological gene networks and GO/pathways for the trait-specific yellow module in PL and PLxD pigs

The ClueGO analysis identified 78 GO/pathway-specific terms associated with the yellow module miRNAs’ target genes in PL and PLxD pigs (Fig. S63). Furthermore, the ClueGO analysis identified 6 functional groups for the trait-associated yellow module miRNAs’ target genes in PL and PLxD pigs (Fig. S64). Finally, the distribution of all 78 module-specific GO/pathways-specific terms, represented in the 6 functional group networks, is visualized in supplementary Figure S63-S65.

## Discussion

The research interest in the dietary effects of PUFAs, particularly the omega-3 and omega-6 PUFAs, has been growing substantially in the past few years (Szostak, et al. 2016, Ogłuszka et al. 2017, Shrestha et al. 2020, Lin et al. 2019, Zhang et al. 2019a, b). As important essential dietary constituents, omega-3 and omega-6 PUFAs play a wide-range of physiological roles. Omega-3 and omega-6 PUFAs regulate lipidomic and biophysical homeostasis improving membrane fluidity to maintain cellular fitness (Levental et al. 2020), improve health by being precursors of anti-inflammatory derivatives (Molfino et al. 2017, Zárate et al. 2017), and regulate foetal programming (Shrestha et al. 2020). Disturbances in omega-6/omega-3 fatty acid ratio due to western diet composition relate to the metabolic syndrome in adults (Mirmiran et al. 2012) and increase the risk for obesity (Simopoulos 2016), obesity-related diseases like type II diabetes, cardiovascular diseases (Bowen et al. 2016), and disorders of lipid metabolism (Jacometo et al. 2014). The triglyceride metabolism in the liver through disturbances in omega-6/omega-3 fatty acid ratio in western diet is closely related to the prevalence of obesity (Simopoulos 2016) and non-alcoholic fatty liver disease (Jump et al. 2018) and direct differentiation of the membrane phenotype in mesenchymal stem cells to potentiate osteogenesis (Levental et al. 2017) and breast cancer (Zanoaga et al. 2018). Dietary PUFAs play significant roles in liver physiology by removal of cholesterol excess from hepatocytes, protection from excessive release of FFA from TG, enhancement of catabolic processes in β-oxidation, or counteraction of liver steatosis presumably through pathways (Terracciano et al. 2018). We performed a functional miRNA gene expression analysis in pigs. Previous studies revealed that the specific effect of dietary PUFAs on hepatic miRNA gene expression depending on the breed of pigs was characterized by distinct fatness, carcass, and growth performance traits (Franco et al. 2014). The present study is based on investigating the dietary effect of fatty acid metabolism; therefore, the best choice for the analysed organ was the liver. Furthermore, we can investigate not only the major mechanism influenced by omega fatty acids on metabolism but additionally indicate the breed-specific differences in pigs fed with PUFAs-enriched diets. Moreover, this study is relevant also for human studies, as an animal model to explain the different predisposition factors to obesity or the ability of lipid storage.

### Trait-associated miRNA hub genes and metabolic pathway analysis

In the WGCNA experiments, we performed the co-expression analysis by identifying the trait-associated hub miRNA genes of the porcine liver transcriptome. Using the WGCNA R script, nine trait-associated modules with *p*-value ≤ 0.05 were identified as statistically significant for the trait in PL and PLxD pigs. As a result, four traits were identified with significant modules: (i) the shoulder subcutaneous fat thickness, (ii) meat colour (a*), (iii) conductivity 24 h post-mortem (PE24), and (iv) ash, respectively. Hepatic miRNA expression could potentially affect meat quality traits via metabolites, e.g. through FA metabolism. These identified trait-associated miRNA modules can be suggested as novel hepatic miRNA trait-associated modules. The intra-modular analysis for MEM (modules in colour) was carried out to identify the relationship between the miRNA and the identified porcine phenotypic trait. Trait-wise, a total of 9, 7, 2, and 8 trait-associated significant modules in PL and PLxD pigs were identified representing four traits. In general, a total of 63 significant (*p*-value ≤ 0.05) porcine hepatic miRNAs were identified representing nine trait-associated modules (Table 2). However, previously numerous studies identified these putative miRNA biomarkers by investigating other transcriptomes including porcine liver, and the phenotypic traits, particularly the porcine growth and development, meat quality, health, and reproduction (Table 2).

**Table 2:** List of significantly trait-correlated module putative porcine hepatic miRNA hub genes identified in this study and list of references which identified these porcine miRNAs in the liver and other tissues (skeletal muscle, adipose, tumour, etc.)

Trait-correlated significant module	Identified miRNA hub genes in trait-associated modules	References
Black	ssc-miR-142-3p, ssc-miR-30a-3p, ssc-miR-21-5p	Su et al. (2017), Kiss et al. (2020), Mármol-Sánchez et al. (2020), Hua et al. (2020), Tang et al. (2022), Li et al. (2021), Wang et al. (2022), Xu et al. (2020)
Blue	ssc-miR-425-5p, ssc-miR-199a-5p, ssc-miR-29a-3p, ssc-miR-122-5p, ssc-miR-199a-3p, ssc-miR-199b-3p, ssc-miR-186-5p, ssc-miR-22-3p	Davoli et al. (2018), Liao et al. (2022), Mármol-Sánchez et al. (2020), Swain et al. (2021), Gao et al. (2022), Gòdia et al. (2020), Yang et al. (2022), Li et al. (2018)
Brown	ssc-miR-486, ssc-miR-423-3p, ssc-miR-181b, ssc-miR-140-3p, ssc-miR-28-3p, ssc-miR-151-3p, ssc-miR-148a-3p, ssc-miR-26b-5p, ssc-miR-30a-5p	Zhang et al. (2022a, b, c), Zhu et al. (2022), Zhang et al. (2018), Zuo et al. (2015), Ding et al. (2020), Xie et al. (2010), Mármol-Sánchez et al. (2020), Zhang et al. (2020)
Green	ssc-miR-7142-3p, ssc-miR-127, ssc-miR-10b, ssc-miR-374a-5p, ssc-miR-146a-5p, ssc-let-7d-5p, ssc-miR-340, ssc-miR-122-3p, ssc-miR-24-3p, ssc-miR-374a-3p, ssc-miR-7134-3p, ssc-miR-423-5p, ssc-miR-15a, ssc-miR-148b-3p	Segura-Wang et al. (2021), Daza et al. (2017), Stachowiak et al. (2017), Li et al. (2015), Li et al. (2019), Kiss et al. (2020), Huang et al. (2019), Zhang et al. (2015a, b), Swain et al. (2021), Zhang et al. (2014), Truong et al. (2023), Wang et al. (2017), Liu et al. (2022), Ding et al. (2020), Zhang et al. (2022a, b, c), Brogaard et al. (2018)
Magenta	ssc-miR-99b, ssc-miR-542-3p, ssc-miR-142-5p, ssc-miR-30e-5p, ssc-miR-92a, ssc-miR-103, ssc-let-7i-5p, ssc-let-7a, ssc-miR-101, ssc-miR-221-3p	Oczkowicz et al. (2022), Jang and Lee (2021), Sun et al. (2021), Grenier et al. (2019), Zhang et al. (2016), Qianqian et al. (2021), Zhang et al. (2022a, b, c), Ding et al. (2020), Zhang et al. (2015a, b), Zuo et al. (2015), Córdoba et al. (2015), Zhang et al. (2020), Timoneda et al. (2012), Wang et al. (2015), Li et al. (2015), Hua et al. (2021), Zhang et al. (2022a), Truong et al. (2023), Song et al. (2020), Zhang et al. (2015a, b)
Pink	ssc-miR-339, ssc-miR-339-5p, ssc-miR-19b, ssc-miR-99a-5p, ssc-miR-30e-3p, ssc-miR-30b-5p	Feng et al. (2022), Jia et al. (2012), Zhang et al. (2019a, b), Kiss et al. (2020), Mármol-Sánchez et al. (2020)
Purple	ssc-miR-125b, ssc-miR-10a-5p, ssc-miR-151-5p, ssc-miR-143-3p, ssc-miR-126-3p	Jang and Lee (2021), Li et al. (2019), Xie et al. (2010), Chen et al. (2017), Kaczmarek et al. (2021), Timoneda et al. (2013), Fleming and Miller (2019), Kiss et al. (2020), Ding et al. (2020), Qianqian et al. (2021), Li et al. (2017), Zuo et al. (2015), Zhong et al. (2020), Stachowiak et al. (2017)
Turquoise	ssc-miR-146b, ssc-miR-1285, ssc-miR-92b-3p, ssc-miR-181c	Tao and Xu (2013), Tao et al. (2017), Oczkowicz et al. (2022), Zhang et al. (2016), Davoli et al. (2018), Stachowiak et al. (2017), Sun et al. (2021), Jang and Lee (2021), Alvarez-Rodriguez et al. (2020), Zhu et al. (2022), Wang et al. (2022)
Yellow	ssc-miR-16, ssc-miR-30c-5p, ssc-miR-126-5p, ssc-miR-194a-5p, ssc-miR-378, ssc-let-7f-5p, ssc-miR-27b-3p, ssc-miR-181a	Jiang et al. (2021), Zhang et al. (2019a, b), Timoneda et al. (2012), Wang et al. (2015), Segura-Wang et al. (2021), Gòdia et al. (2020), Zuo et al. (2015), Yang et al. (2022), Hou et al. (2012), Daza et al. (2017), Hua et al. (2020), Ye et al. (2012), Lian et al. (2012)

#### Trait-associated miRNA hub genes and metabolic pathways in the trait-specific black module

We identified the putative porcine hepatic miRNAs ssc-miR-142-3p, ssc-miR-30a-3p, and ssc-miR-21-5p, respectively. Several of these miRNAs have been identified before. The GO and KEGG pathway analysis indicated that the target genes of these miRNAs are mainly associated with cell proliferation, apoptosis, necrosis, inflammation, and fibrosis (Su et al. 2017). In the porcine *gluteus medius* muscle, ssc-miR-148a-3p, ssc-miR-22-3p, and ssc-miR-1 DE-miRNAs were identified to play key roles in the regulation of glucose and lipid metabolism. The study showed that seven miRNAs (ssc-miR-148a-3p, ssc-miR-151-3p, ssc-miR-30a-3p, ssc-miR-30e-3p, ssc-miR-421-5p, ssc-miR-493-5p, and ssc-miR-503) putatively interact with the PDK4 mRNA, one of the master regulators of glucose utilization and fatty acid oxidation (Mármol-Sánchez et al. 2020; Cai et al. 2023). We identified ssc-miR-21, a well-known biomarker that plays a significant role in the pathogenesis of inflammatory diseases. Elevated ssc-miR-21-5p (miR-21) levels in porcine circovirus type 2 (PCV2) infected porcine kidney 15 (PK-15) cells caused postweaning multisystemic wasting syndrome (PMWS) and other PCV-associated diseases (PCVADs). Furthermore, miR-21 overexpression induced the NF-κB pathway along with inflammation in cells exposed to PCV2 (Li et al. 2021). We conclude that the miRNAs in this module affect both muscle composition and thereby meat quality and porcine health.

#### Trait-associated miRNA hub genes and metabolic pathways in the trait-specific blue modules

We identified the putative porcine hepatic miRNAs ssc-miR-425-5p, ssc-miR-199a-5p, ssc-miR-29a-3p, ssc-miR-122-5p, ssc-miR-199a-3p, ssc-miR-199b-3p, ssc-miR-186-5p, and ssc-miR-22-3p, respectively. Several of these miRNAs have been identified before.

By investigating the divergent backfat deposition trait in Italian Large White pig backfat tissue, Davoli et al. (2018) detected 31 significant DE-miRNAs, including 14 upregulated (including ssc-miR-132, ssc-miR-146b, ssc-miR-221-5p, ssc-miR-365-5p, and ssc-moR-21-5p) and 17 downregulated (including ssc-miR-136, ssc-miR-195, ssc-miR-199a-5p, and ssc-miR-335). In other studies, numerous miRNAs and mRNA networks were expressed related to backfat-related pig QTL (Davoli et al. 2018). The ssc-miR-122-5p and ssc-miR-192 miRNAs were downregulated in porcine adipose tissue affected by feed efficiency in pigs (Liao et al. 2022). Furthermore, the GO and KEGG analyses indicated that these miRNAs were significantly related to lipid metabolism, and these miRNAs modulated FE by regulating lipid metabolism. Mármol-Sánchez et al. (2020) investigated the porcine gluteus medius muscle miRNA expression before and after food intake in gilts and identified ssc-miR-148a-3p, ssc-miR-22-3p, and ssc-miR-1, which play key roles in the regulation of glucose and lipid metabolism. We conclude that the miRNAs in this module affect feed efficiency and lipid metabolism, which are major traits in our feed supplementation study.

#### Trait-associated miRNA hub genes and metabolic pathways in the trait-specific brown modules

We identified the putative porcine hepatic miRNAs ssc-miR-486, ssc-miR-423-3p, ssc-miR-181b, ssc-miR-140-3p, ssc-miR-28-3p, ssc-miR-151-3p, ssc-miR-148a-3p, ssc-miR-26b-5p, and ssc-miR-30a-5p, respectively. Several of these miRNAs have been identified before. Regulatory functions of miRNAs in the subcutaneous adipose tissue of Laiwu and Large White (LW) pig breeds identified 39 known miRNAs and 56 novel miRNAs, respectively, in the LW and LY pig breeds (Zhang et al. (2022a, b, c). The Gene Ontology and KEGG pathway analysis identified predicted miRNAs that were involved in several fat-associated pathways, such as the peroxisome proliferator-activated receptor (PPAR), mitogen-activated protein kinases (MAPK), and Wnt signalling pathways. Ssc-miR-133a-3p, ssc-miR-486, and ssc-miR-1 biomarkers regulated the development of porcine subcutaneous fat through the PPAR signalling pathway (Zhang et al. 2022a, b, c). Ssc-miR-1 and ssc-miR-133, belonging to the MyomiRs, regulated muscle myosin content, myofiber identity, and muscle performance. The overexpression and inhibition of ssc-miR-143-3p in porcine skeletal muscle satellite cells induced the increase and reduction of the slow muscle fibre gene and protein (MYH7), indicating that miR-143 activity regulated muscle fibre differentiated in skeletal muscle (Zuo et al. 2022). We conclude that the miRNAs in this module may be involved in the regulation of fatty acid metabolism and muscle development.

#### Identification of the trait-associated miRNA hub genes and the metabolic pathways in the trait-specific green modules

We identified the putative porcine hepatic miRNAs ssc-miR-7142-3p, ssc-miR-127, ssc-miR-10b, ssc-miR-374a-5p, ssc-miR-146a-5p, ssc-let-7d-5p, ssc-miR-340, ssc-miR-122-3p, ssc-miR-24-3p, ssc-miR-374a-3p, ssc-miR-7134-3p, ssc-miR-423-5p, ssc-miR-15a, and ssc-miR-148b-3p, respectively. Several of these miRNAs have been identified before. The porcine liver and jejunum transcriptome profiling showed that ssc-miR-10b is downregulated in the liver of deoxynivalenol-exposed pigs. The identified predicted microRNA target genes showed enrichment of pathways including PIK3-AKT, Wnt/β-catenin, and adherents’ junctions (Segura-Wang et al. 2021). miRNA expression profiling of the *longissimus dorsi* (LD) muscle of a Duroc × Pietrain resource population showed that the most abundant miRNAs were ssc-miR-1, ssc-miR-133a-3p, ssc-miR-378, ssc-miR-206, and ssc-miR-10b (Daza et al. 2017). In a similar transcriptome study, ssc-miR-146a-5p and ssc-miR-221-5p miRNAs were upregulated in LPS-challenged pig skeletal muscle. We conclude that the miRNAs in this module in the liver potentially regulated muscle development and immune traits.

#### Identification of the trait-associated miRNA hub genes and the metabolic pathways in the trait-specific magenta modules

We identified the putative porcine hepatic miRNAs ssc-miR-99b, ssc-miR-542-3p, ssc-miR-142-5p, ssc-miR-30e-5p, ssc-miR-92a, ssc-miR-103, ssc-let-7i-5p, ssc-let-7a, ssc-miR-101, and ssc-miR-221-3p, respectively. Several of these miRNAs have been identified before in a dietary fat study related to adipose tissue and circulating miRNAs. Various dietary sources of fat (rapeseed oil, beef tallow, coconut oil) affected the miRNA profile in pig adipose tissue, which is the main source of circulating miRNAs. Coconut oil showed the highest number of differentially expressed miRNAs (Oczkowicz et al. (2022). The study proposed a subset of diet-related, adipose-specific, conservative miRNAs: ssc-miR-99b, ssc-miR-4334-3p, ssc-miR-146b, and ssc-miR-23a (Shen et al. 2016). Around weaning the small intestine of pigs, 133 candidate targets for miR-196a were identified using a target prediction database (Jang and Lee 2021). Gene ontology and Kyoto Encyclopaedia of Genes and Genomes (KEGG) pathway analyses showed that the target genes were associated with 19 biological processes, 4 cellular components, 8 molecular functions, and 7 KEGG pathways, including anterior/posterior pattern specification as well as cancer, PI3K-Akt, MAPK, GnRH, and neurotrophin signalling pathways (Jang and Lee 2021). We conclude that the miRNAs in this module potentially affected the regulation of adipose-specific circulating miRNAs potentially affecting several signalling traits in a dietary-fat-specific trait.

#### Trait-associated miRNA hub genes and metabolic pathways in the trait-specific pink modules

We identified the putative porcine hepatic miRNAs ssc-miR-339, ssc-miR-339-5p, ssc-miR-19b, ssc-miR-99a-5p, ssc-miR-30e-3p, and ssc-miR-30b-5p, respectively. Several of these miRNAs have been identified before. Feng et al. (2022) identified 265 differentially expressed circRNAs, of which 187 upregulated circRNA and 78 downregulated circRNA in IMF in the regulation of intramuscular and subcutaneous adipose tissue of Laiwu pigs. The host genes were mainly involved in GO terms and signalling pathways related to adipogenesis. Functional annotation of indirect target genes and protein network analysis showed that circRNA_06424 affects the expression of PPARD, MMP9, UBA7, and other indirect target genes by competitively binding to miRNAs such as ssc-miR-339-5p, ssc-miR-744, and ssc-miR-328 and participates in signalling pathways such as the PPAR signalling pathway, Wnt signalling pathway, unsaturated fatty acid, and other signalling pathways, resulting in the difference of fat deposition between IMF and SCF (Feng et al. 2022). Ssc-miR-339-5p and ssc-miR-532-3p, targeting the G6PC 3′ untranslated region, were significantly upregulated by a low protein sow diet only in newborn Meishan females suggesting that a maternal low-protein diet during pregnancy causes hepatic activation of G6PC gene expression in male pigs, which possibly contributes to adult-onset hyperglycemia (Jia et al. 2012). We conclude that the miRNAs in this module may be involved in the regulation and differential filling of different fat depots due to both dietary fat and protein.

#### Trait-associated miRNA hub genes and metabolic pathways in the trait-specific purple modules

We identified the putative porcine hepatic miRNAs ssc-miR-125b, ssc-miR-10a-5p, ssc-miR-151-5p, ssc-miR-143-3p, and ssc-miR-126-3p, respectively. Several of these miRNAs have been identified before. miRNA expression profiling in the small intestine of pigs around weaning identified 38 DE miRNAs related to weaning, including ssc-miR-196a, ssc-miR-451, ssc-miR-499-5p, ssc-miR-7135-3p, ssc-miR-144, ssc-miR-542-3p, ssc-miR-214, ssc-miR-758, ssc-miR-4331, ssc-miR-105–1, ssc-miR-1285, ssc-miR-10a-5p, ssc-miR-4332, ssc-miR-503, ssc-miR-6782-3p, and ssc-miR-424-5p, respectively. The target genes were associated with 19 biological processes, 4 cellular components, 8 molecular functions, and 7 KEGG pathways, including anterior/posterior pattern specification and several signalling pathways (Jang et al. 2021). We conclude that the miRNAs in this module may be involved in the regulation of small intestine function around weaning, which is known for dietary reactions.

#### Trait-associated miRNA hub genes and metabolic pathways in the trait-specific turquoise modules

We identified the putative porcine hepatic miRNAs ssc-miR-146b, ssc-miR-1285, ssc-miR-92b-3p, and ssc-miR-181c, respectively. Several of these miRNAs have been identified before. Tao et al. (2017) investigated the porcine miRNA transcriptome of the small intestine during weaning and suckling pigs. The study identified a total of 136 differentially expressed miRNAs. Overexpression of miR-146b plays a significant regulatory role in intestinal epithelial cell viability, proliferation, and apoptosis in pigs. We conclude that the miRNAs in this module may be involved in the regulation of dietary effects on intestinal epithelial cell function.

#### Trait-associated miRNA hub genes and metabolic pathways in the trait-specific yellow modules

We identified the putative porcine hepatic miRNAs ssc-miR-16, ssc-miR-30c-5p, ssc-miR-126-5p, ssc-miR-194a-5p, ssc-miR-378, ssc-let-7f-5p, ssc-miR-27b-3p, and ssc-miR-181a, respectively. Several of these miRNAs have been identified before. Jiang et al. (2021) identified 63 DE miRNAs between biceps femoris vs. soleus, a fast-twitch and a slow-twitch muscle, respectively, including four skeletal muscle-highly expressed miRNAs, ssc-miR-378, ssc-let-7f, ssc-miR-26a, and ssc-miR-27b-3p. Furthermore, Zhang et al. (2019a, b) showed that dietary resveratrol supplementation increased the IMF content and decreased serum lipids levels and enhanced the expression of ssc-miR-181a, ssc-miR-370, and ssc-miR-21 and reduced the expression of ssc-miR-27a in longissimus dorsi. Resveratrol is a strong antioxidant to remove free radicals. We conclude that the miRNAs in this module may be related to meat quality traits via muscle fibre type and intramuscular fat levels. Furthermore, the miRNAs may be related to overall health via the regulation of antioxidant activity.

## Conclusions

Our study identified ten modules with co-expressed miRNAs in the porcine liver transcriptome. It shows that both investigated breeds in each dietary group miRNA expression profiles are characterized by the strong interconnection between miRNAs, which are highly co-expressed with each other. The study identified phenotypic traits correlated with co-expressed miRNAs. Detected modules are significantly correlated with PE24, meat colour, shoulder subcutaneous fat thickness, and ash traits. Therefore, the identified miRNAs from significant modules of mentioned phenotypic traits can be considered predicted miRNA genes associated with meat and carcass traits. Further investigation is needed to validate their potential functionality. Among all trait-correlated modules, targets for co-expressed miRNAs were identified. The identified co-expressed miRNA affects the phenotypic traits probably by specific functional pathways regulating the expression of target genes. The study identified the target genes in each module for each significantly correlated trait. The identified miRNA genes with the highest module membership are strongly predicted miRNA genes for the investigated traits. The identified hepatic miRNA target gene expression networks and metabolic pathways in trait-specific modules in PL and PLxD pigs could play a significant role in biological functions such as (i) muscle tissue development, (ii) different cellular processes and developments, (iii) system development, (iv) metabolic processes, and (v) adipose tissue development.

## Supplementary Information

Below is the link to the electronic supplementary material.

Supplementary file1 (PDF 5600 kb)

## Data Availability

All data obtained in this study (protocol, miRNA-seq sequences) is fully presented in the article and supplementary materials.
