# Supplementary material for: Identification of trait-associated microRNA modules in liver transcriptome of pig fed with PUFAs-enriched supplementary diet
Source: J Appl Genet. 2024 Nov 15;66(2):389–407. doi: 10.1007/s13353-024-00912-w (PMC12000271; doi:10.1007/s13353-024-00912-w)
Supplement: Supplementary file 1 — Supplementary file1 (PDF 5600 kb) [file 13353_2024_912_MOESM1_ESM.pdf]

Supplementary Table S1. A list of investigated phenotypic traits utilized in WGCNA analysis of PL and PLxD pigs.

Trait	Abbreviation
Body growth related at end of fattening	BWF
Daily gains	DG
Body growth related	BW
Meatiness	M
GMP	GMP
Backfat thickness	BFT
Shoulder subcutanesous fat thickness	SSFT
pH 45 min post mortem	PH45
Conductivity 24 hours post mortem	PE24
Water Holding Capacity	WHC
Drip loss	DL
pH 24 hours post mortem	PH24
L* - meat color	L*
a* - meat color	a*
b* - meat color	b*
Fat	FAT
Protein	Pr
Ash	ASH
Dry matter	DM
Trait	Abbreviation
C16:0	C16:0
C16:1	C16:1
C18:0	C18:0
C18:1n9	C18:1n9
C18:1n7	C18:1n7
C18:2n6	C18:2n6
C18:3n3	C18:3n3
C20:4n6	C20:4n6
n-6	n-6
n-3	n-3
n-6/n-3	n-6/n-3
PUFA	PUFA
MUFA	MUFA
Saturated Fatty Acid	SFA
PUFA/SFA	PUFA/SFA

Supplementary Table S2. The number of target genes found for porcine miRNA with target score > 90.

miRNA	Number of target genes	module
ssc-miR-30e-5p	520	magenta
ssc-miR-30b-5p	518	pink
ssc-miR-30c-5p	518	yellow
ssc-miR-30a-5p	517	brown
ssc-miR-27b-3p	326	yellow
ssc-miR-26b-5p	322	brown
ssc-miR-92b-3p	312	turquoise
ssc-miR-186-5p	285	blue
ssc-miR-126-5p	283	yellow
ssc-miR-29a-3p	252	blue
ssc-miR-148a-3p	211	brown
ssc-miR-148b-3p	211	green
ssc-miR-374a-5p	186	green
ssc-let-7i-5p	184	magenta
ssc-let-7f-5p	181	yellow
ssc-miR-30e-3p	162	pink
ssc-miR-30a-3p	161	black
ssc-miR-107	148	green
ssc-let-7d-5p	146	green
ssc-miR-24-3p	143	green
ssc-miR-142-5p	141	magenta
ssc-miR-143-3p	91	purple
ssc-miR-199a-5p	86	blue
ssc-miR-199a-3p	81	blue
ssc-miR-199b-3p	81	blue
ssc-miR-221-3p	80	magenta
ssc-miR-142-3p	73	black
ssc-miR-21-5p	72	black
ssc-miR-22-3p	60	blue
ssc-miR-374a-3p	54	green
ssc-miR-140-3p	49	brown
ssc-miR-423-5p	38	green
ssc-miR-542-3p	38	magenta
ssc-miR-146a-5p	35	green
ssc-miR-122-5p	34	blue
ssc-miR-10a-5p	30	purple
ssc-miR-148a-5p	27	red
ssc-miR-339-5p	20	pink
ssc-miR-425-5p	18	blue
ssc-miR-28-3p	14	brown
ssc-miR-99a-5p	7	pink

ssc-miR-122-3p	2	green
ssc-miR-126-3p	1	purple
ssc-miR-423-3p	1	brown

Supplementary Table S3. Identification of target genes with a target score > 90 for porcine miRNAs in each of the modules.

Module	Number of target genes	miRNA	Highest target score gene	Target Score
Yellow	1308	ssc-miR-126-5p	DENND1B	99.9999999806704
		ssc-miR-126-5p	FAM168A	99.9999992954528
		ssc-miR-126-5p	GRIK2	99.999997669618
		ssc-miR-126-5p	RFX4	99.9999894463683
		ssc-miR-126-5p	GNE	99.9998900518947
Brown	1114	ssc-miR-30a-5p	CELSR3	99.998490412936
		ssc-miR-30a-5p	PPARGC1B	99.9829001483369
		ssc-miR-30a-5p	WDR7	99.9536578864795
		ssc-miR-30a-5p	STOX2	99.9306249844605
		ssc-miR-30a-5p	ANKRA2	99.8285694902398
Green	963	ssc-miR-107	DICER1	99.9830481731394
		ssc-let-7d-5p	TRIM71	99.9620105034215
		ssc-miR-146a-5p	TRAF6	99.9583778416227
		ssc-let-7d-5p	HMGA2	99.9464199196979
		ssc-miR-374a-5p	PRDM11	99.9019803393543
Magenta	963	ssc-miR-30e-5p	CELSR3	99.998490412936
		ssc-let-7i-5p	TRIM71	99.9837730009893
		ssc-miR-30e-5p	PPARGC1B	99.9829001483369
		ssc-miR-30e-5p	WDR7	99.9536578864795
		ssc-let-7i-5p	HMGA2	99.9502531848861
Blue	897	ssc-miR-29a-3p	TET3	99.9994760552698
		ssc-miR-186-5p	TBL1XR1	99.9855216659122

		ssc-miR-186-5p	GABRA4	99.9746693277311
		ssc-miR-186-5p	STK17B	99.9742948949727
		ssc-miR-22-3p	GRM5	99.9270382681564
Pink	707	ssc-miR-30b-5p	CELSR3	99.998490412936
		ssc-miR-30b-5p	PPARGC1B	99.9829001483369
		ssc-miR-30b-5p	WDR7	99.9536578864795
		ssc-miR-30b-5p	STOX2	99.9306249844605
		ssc-miR-30e-3p	PCLO	99.8725051981171
Turquoise	312	ssc-miR-92b-3p	CD69	99.9846637529554
		ssc-miR-92b-3p	MAN2A1	99.9084233380634
		ssc-miR-92b-3p	SLC12A5	99.7980543780163
		ssc-miR-92b-3p	FBXW7	99.66863766777
		ssc-miR-92b-3p	SLC12A5	99.6523443307826
Black	306	ssc-miR-30a-3p	PCLO	99.8725051981171
		ssc-miR-30a-3p	NUFIP2	99.7764011073975
		ssc-miR-30a-3p	CDC73	99.6234578884547
		ssc-miR-142-3p	TASOR2	99.616660325564
		ssc-miR-30a-3p	ZEB2	99.526955288982
Purple	122	ssc-miR-143-3p	ABL2	99.4866669688238
		ssc-miR-143-3p	DENND1B	99.1801630196855
		ssc-miR-143-3p	ABL2	99.0902856466229
		ssc-miR-143-3p	VASH1	99.0834705051181
		ssc-miR-10a-5p	CADM2	98.88699985084
Red	27	ssc-miR-148a-5p	FAM169A	99.2989908804394
		ssc-miR-148a-5p	GSR	97.0207929388
		ssc-miR-148a-5p	ZFP42	96.6976333192
		ssc-miR-148a-5p	PHTF2	96.5165090438856
		ssc-miR-148a-5p	SIX4	96.2874152912125

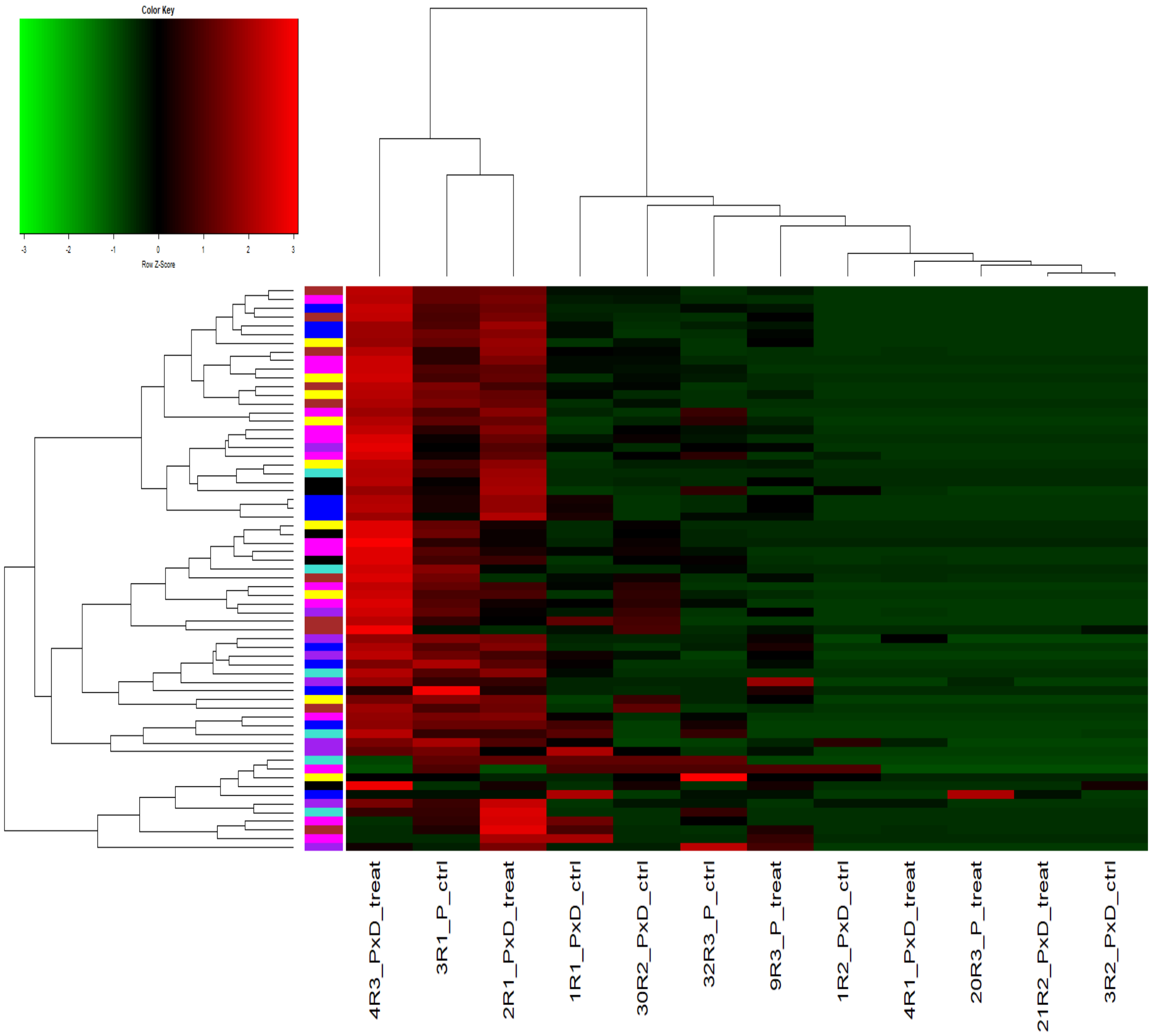

Supp. Figure 1. Expression heatmap of modules significantly correlated with the **Shoulder subcutaneous fat thickness** trait. The greener color the lower expression while the redder color the higher expression. Columns represent samples. On top the dendrogram based on euclidian distance of samples miRNA expression profile. On the bottom sample ids. Rows represent miRNAs. On the left, dendrogram based on euclidian distance of miRNAs expression and the color which symbolize the module membership of miRNA.

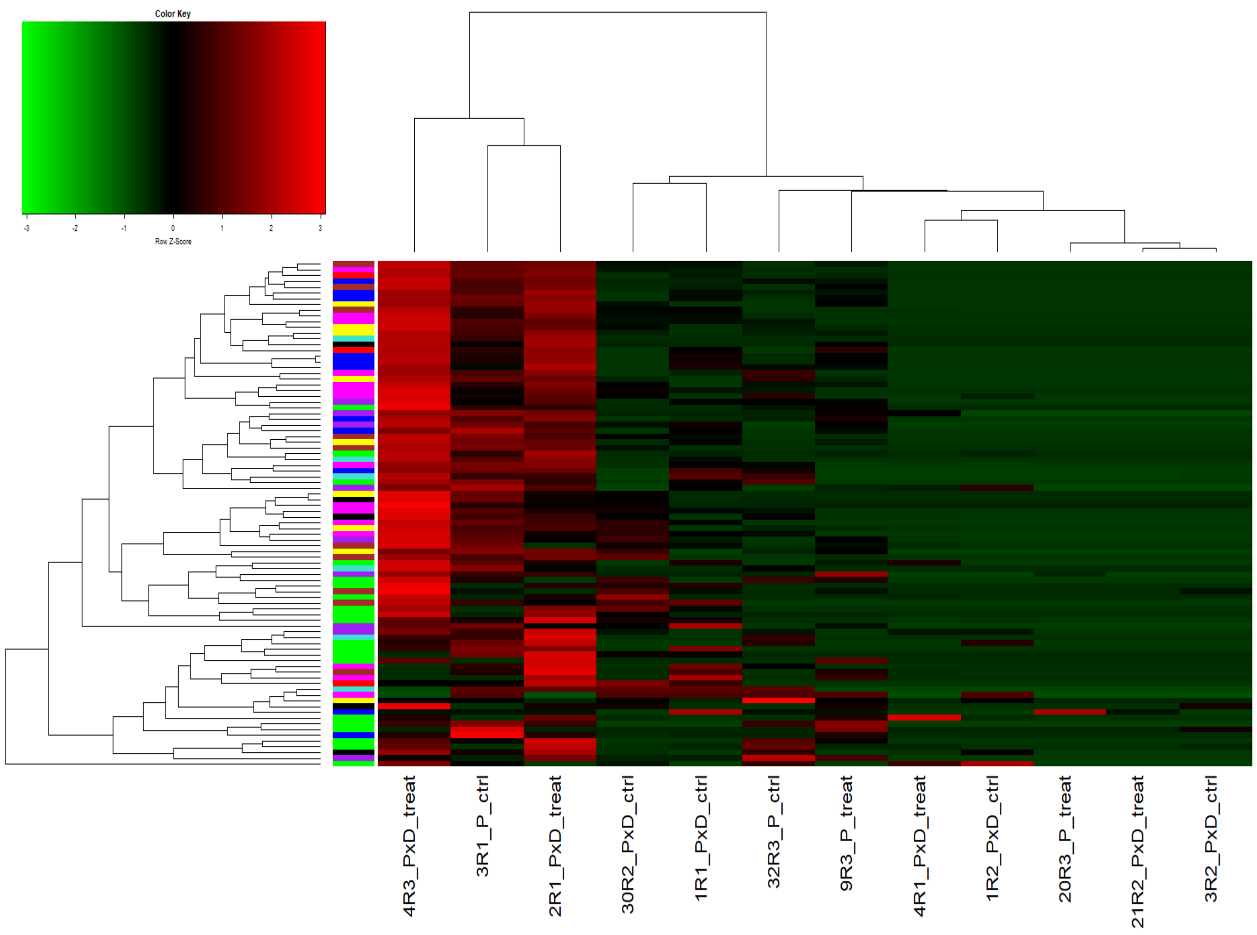

Supp. Figure 2. Expression heatmap of modules significantly correlated with the **a*** trait. The greener color the lower expression while the redder color the higher expression. Columns represent samples. On top the dendrogram based on euclidian distance of samples miRNA expression profile. On the bottom sample ids. Rows represent miRNAs. On the left, dendrogram based on euclidian distance of miRNAs expression and the color which symbolize the module membership of miRNA.

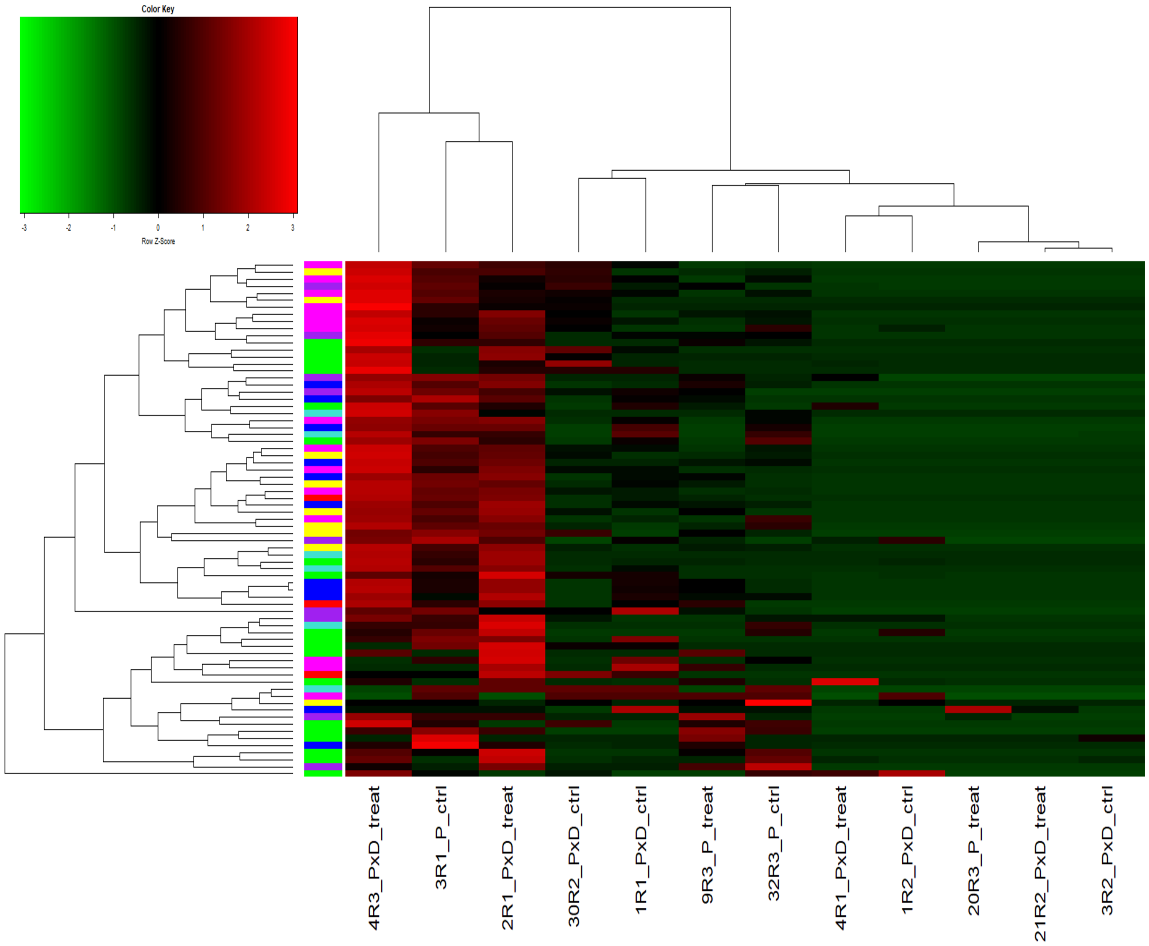

Supp. Figure 3. Expression heatmap of modules significantly correlated with the **Ash** trait. The greener color the lower expression while the redder color the higher expression. Columns represent samples. On top the dendrogram based on euclidian distance of samples miRNA expression profile. On the bottom sample ids. Rows represent miRNAs. On the left, dendrogram based on euclidian distance of miRNAs expression and the color which symbolize the module membership of miRNA.

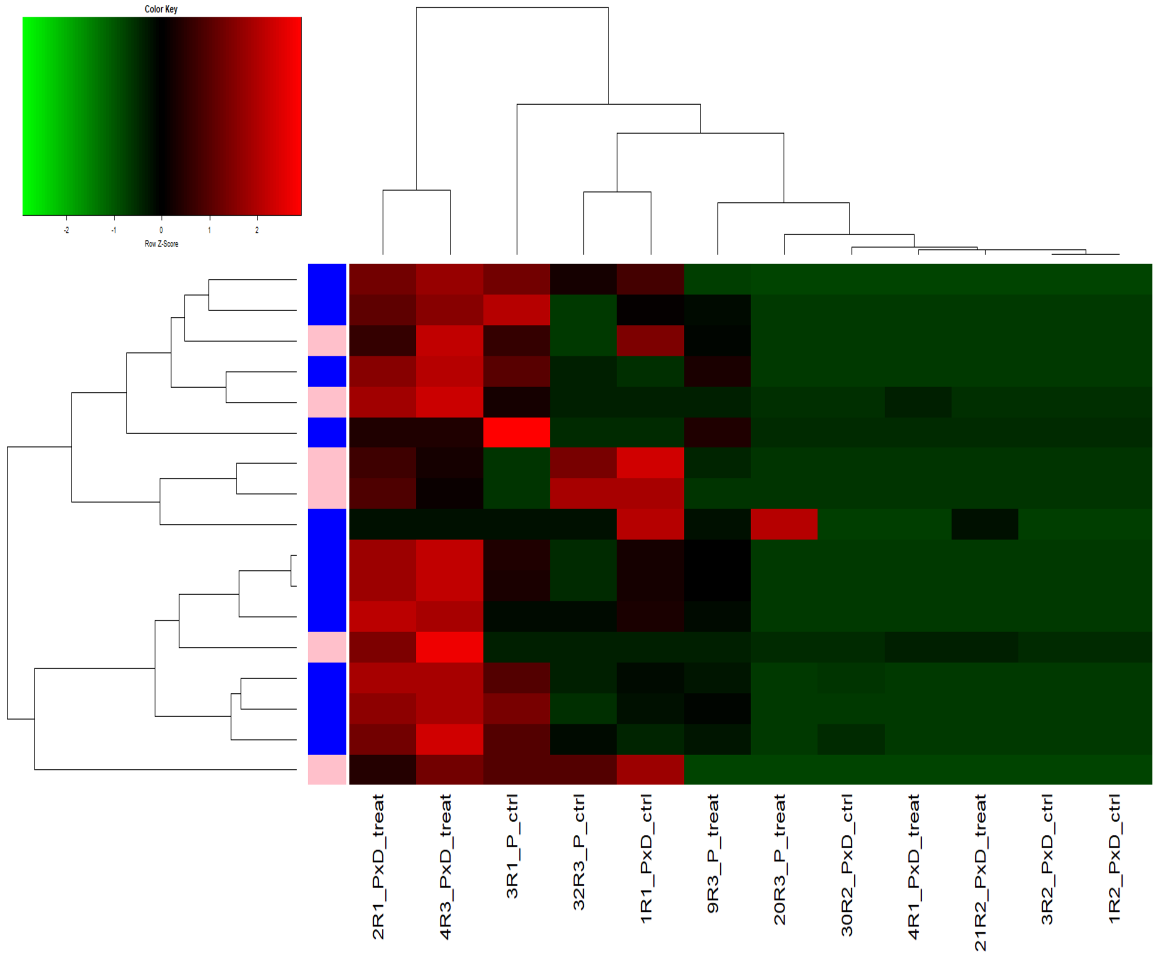

Supp. Figure 4. Expression heatmap of modules significantly correlated with the **PE24** trait. The greener color the lower expression while the redder color the higher expression. Columns represent samples. On top the dendrogram based on euclidian distance of samples miRNA expression profile. On the bottom sample ids. Rows represent miRNAs. On the left, dendrogram based on euclidian distance of miRNAs expression and the color which symbolize the module membership of miRNA.

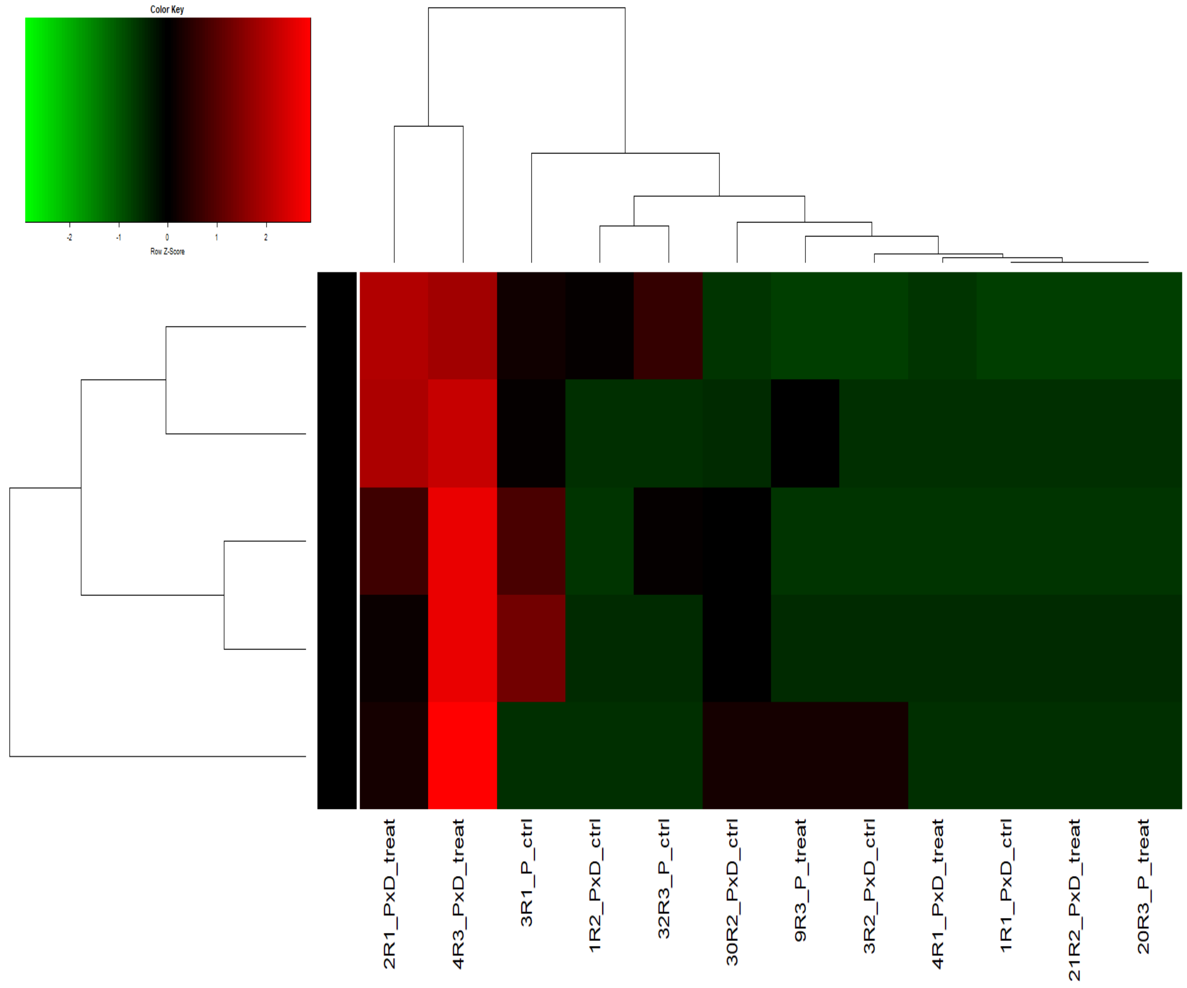

Supp. Figure 5. Expression heatmap of black module. The greener color the lower expression while the redder color the higher expression. Columns represent samples. On top the dendrogram based on euclidian distance of samples miRNA expression profile. On the bottom sample ids. Rows represent miRNAs. On the left, dendrogram based on euclidian distance of miRNAs expression and the color which symbolize the module membership of miRNA.

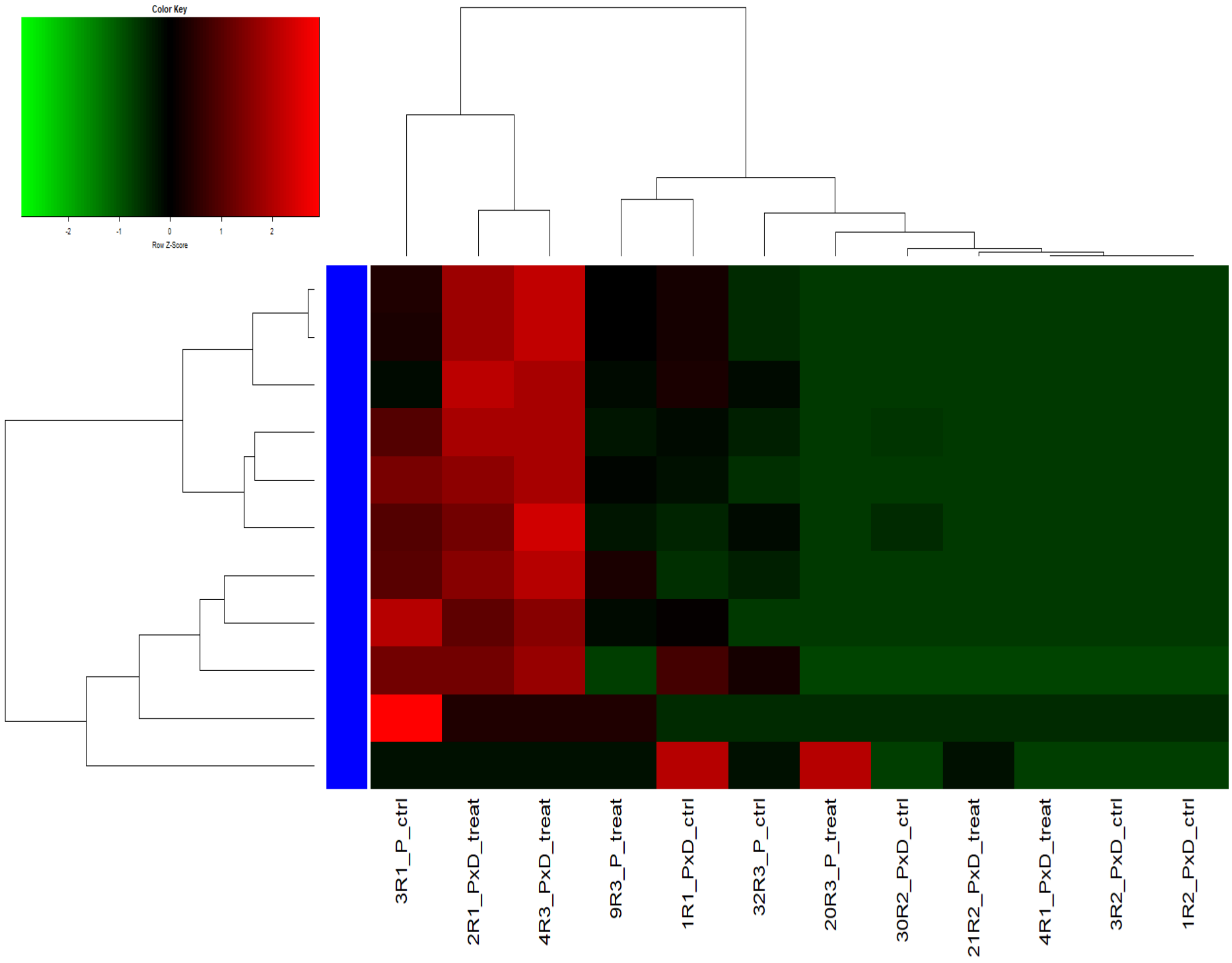

Supp. Figure 6. Expression heatmap of blue module. The greener color the lower expression while the redder color the higher expression. Columns represent samples. On top the dendrogram based on euclidian distance of samples miRNA expression profile. On the bottom sample ids. Rows represent miRNAs. On the left, dendrogram based on euclidian distance of miRNAs expression and the color which symbolize the module membership of miRNA.

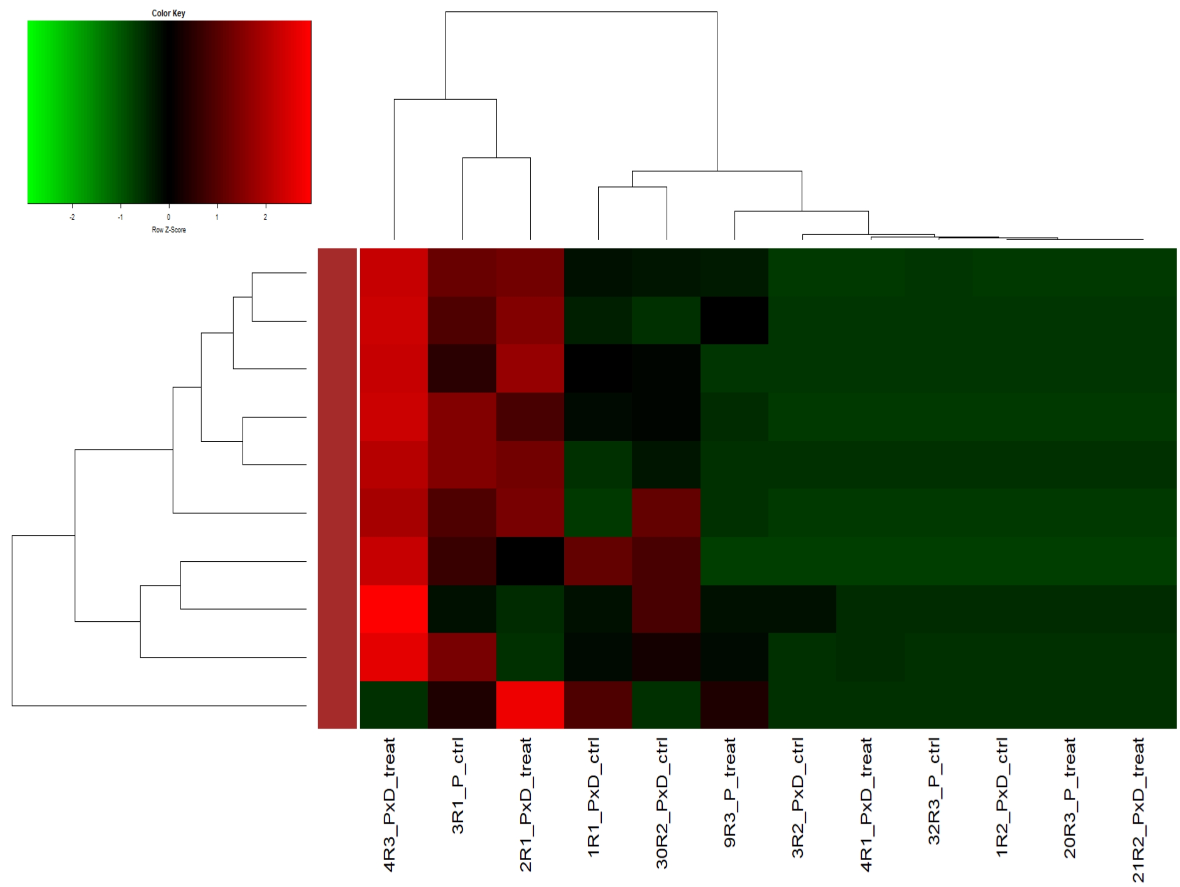

Supp. Figure 7. Expression heatmap of brown module. The greener color the lower expression while the redder color the higher expression. Columns represent samples. On top the dendrogram based on euclidian distance of samples miRNA expression profile. On the bottom sample ids. Rows represent miRNAs. On the left, dendrogram based on euclidian distance of miRNAs expression and the color which symbolize the module membership of miRNA.

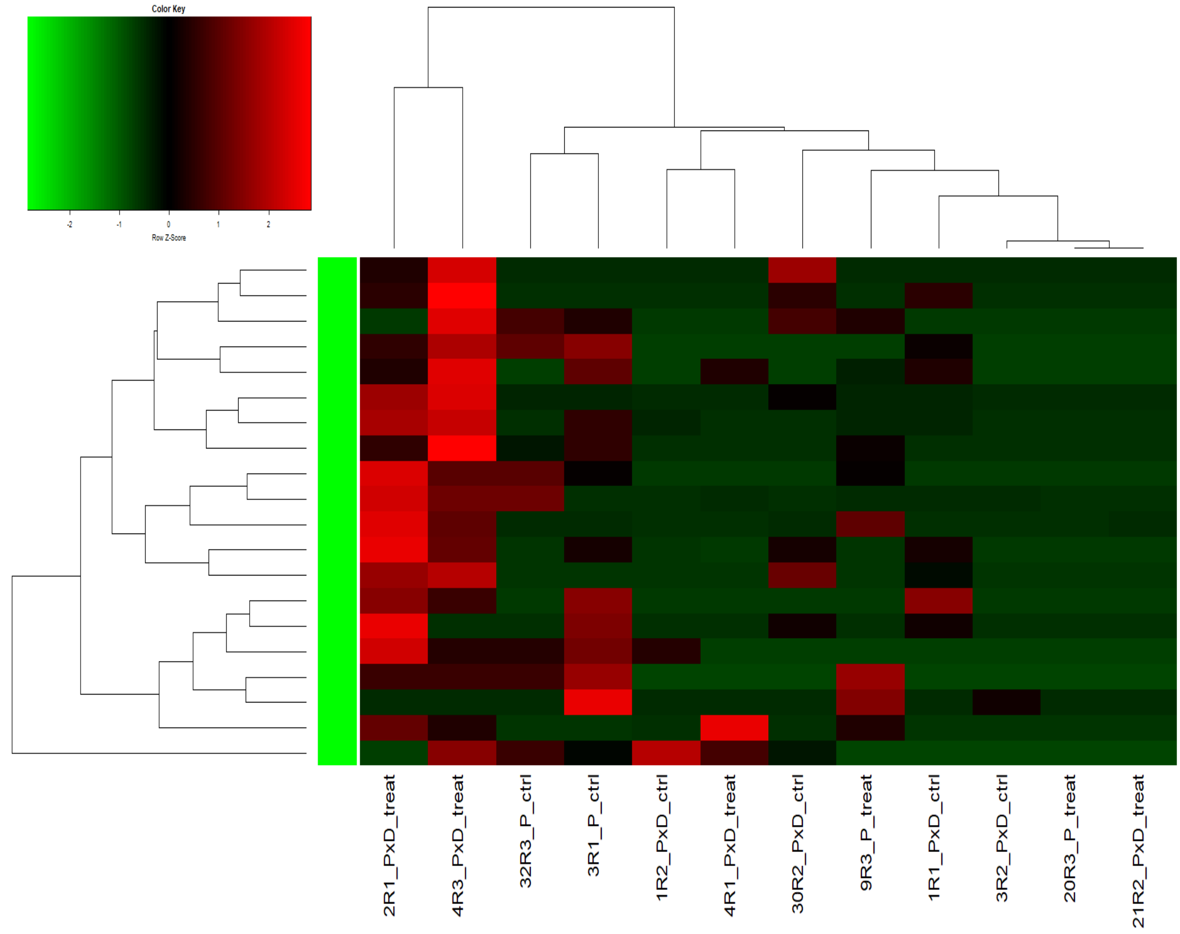

Supp. Figure 8. Expression heatmap of green module. The greener color the lower expression while the redder color the higher expression. Columns represent samples. On top the dendrogram based on euclidian distance of samples miRNA expression profile. On the bottom sample ids. Rows represent miRNAs. On the left, dendrogram based on euclidian distance of miRNAs expression and the color which symbolize the module membership of miRNA.

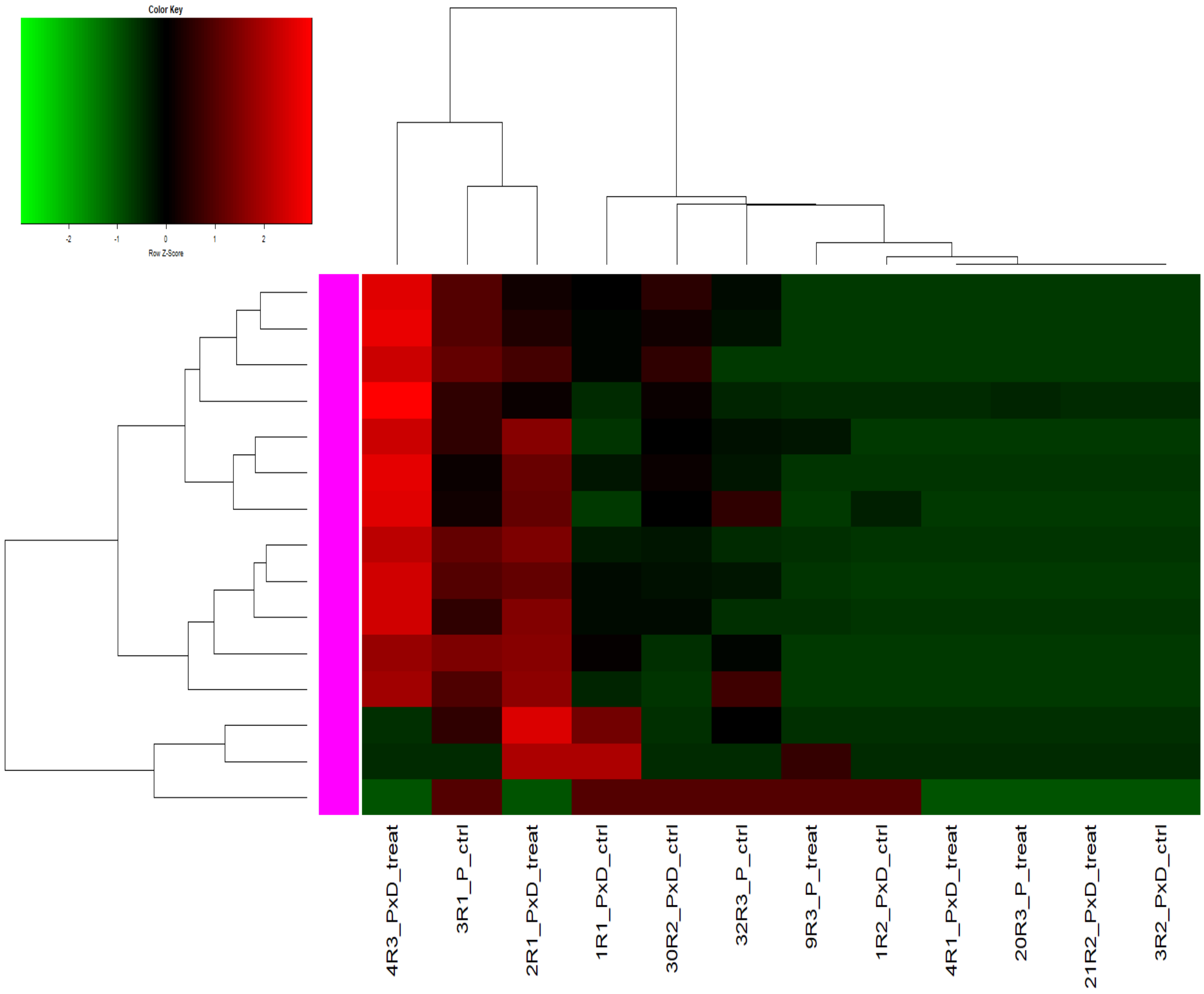

Supp. Figure 9. Expression heatmap of magenta module. The greener color the lower expression while the redder color the higher expression. Columns represent samples. On top the dendrogram based on euclidian distance of samples miRNA expression profile. On the bottom sample ids. Rows represent miRNAs. On the left, dendrogram based on euclidian distance of miRNAs expression and the color which symbolize the module membership of miRNA.

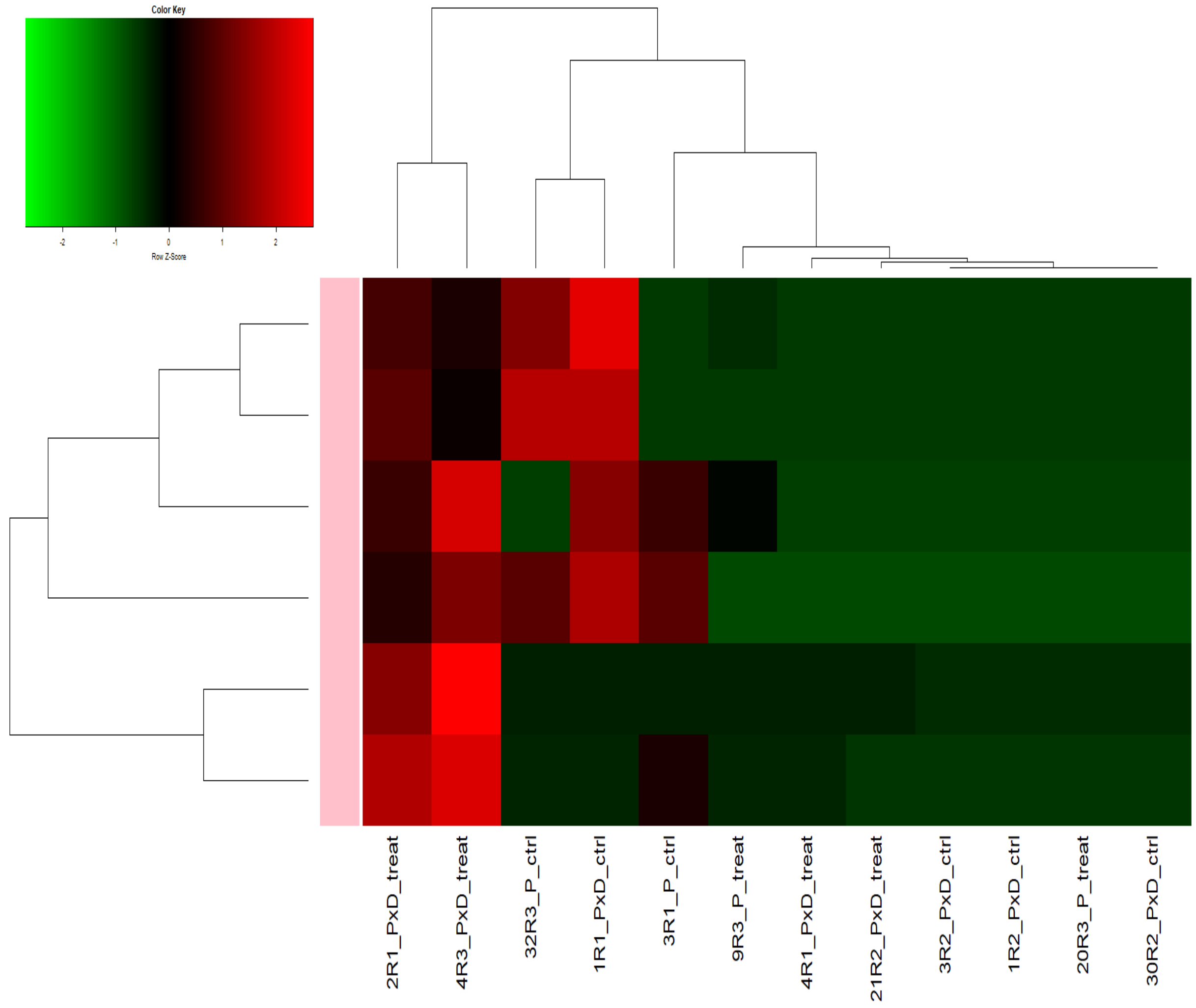

Supp. Figure 10. Expression heatmap of pink module. The greener color the lower expression while the redder color the higher expression. Columns represent samples. On top the dendrogram based on euclidian distance of samples miRNA expression profile. On the bottom sample ids. Rows represent miRNAs. On the left, dendrogram based on euclidian distance of miRNAs expression and the color which symbolize the module membership of miRNA.

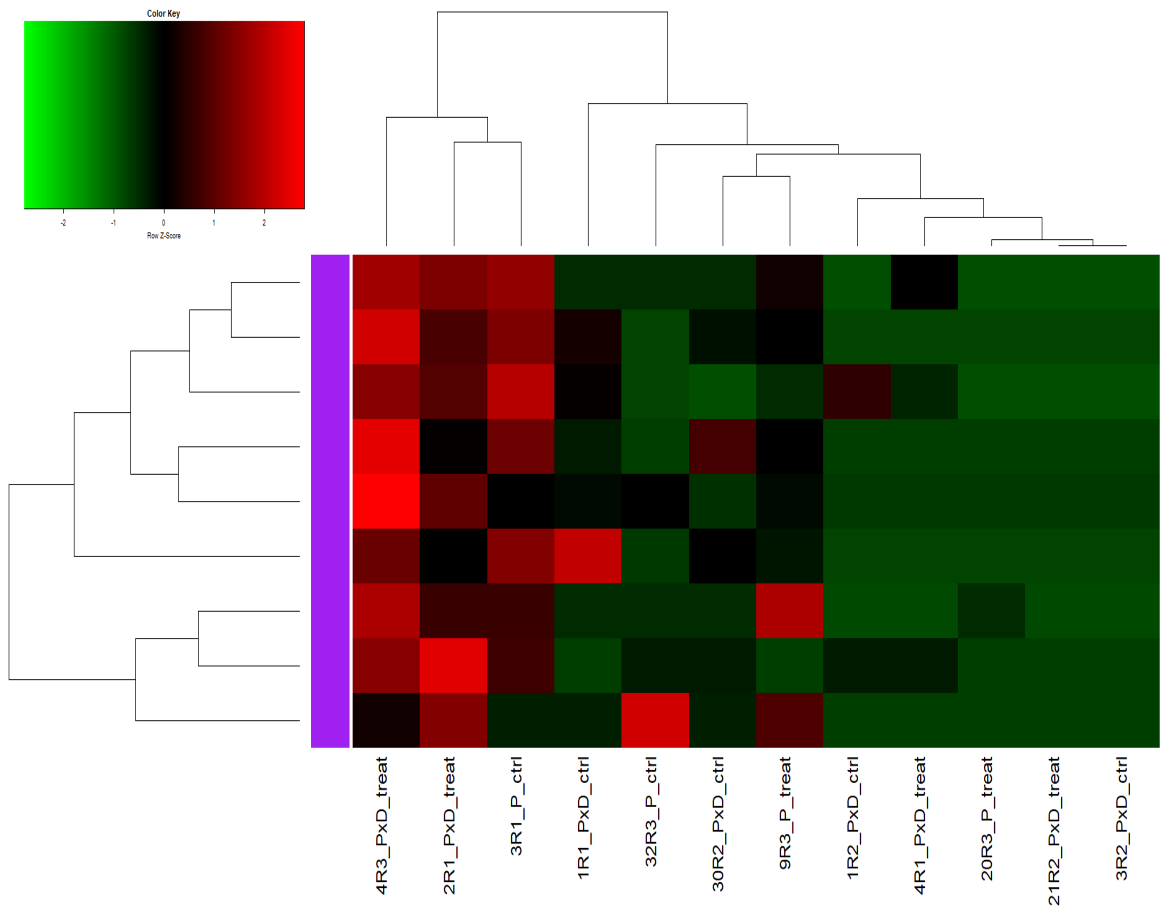

Supp. Figure 11. Expression heatmap of purple module. The greener color the lower expression while the redder color the higher expression. Columns represent samples. On top the dendrogram based on euclidian distance of samples miRNA expression profile. On the bottom sample ids. Rows represent miRNAs. On the left, dendrogram based on euclidian distance of miRNAs expression and the color which symbolize the module membership of miRNA.

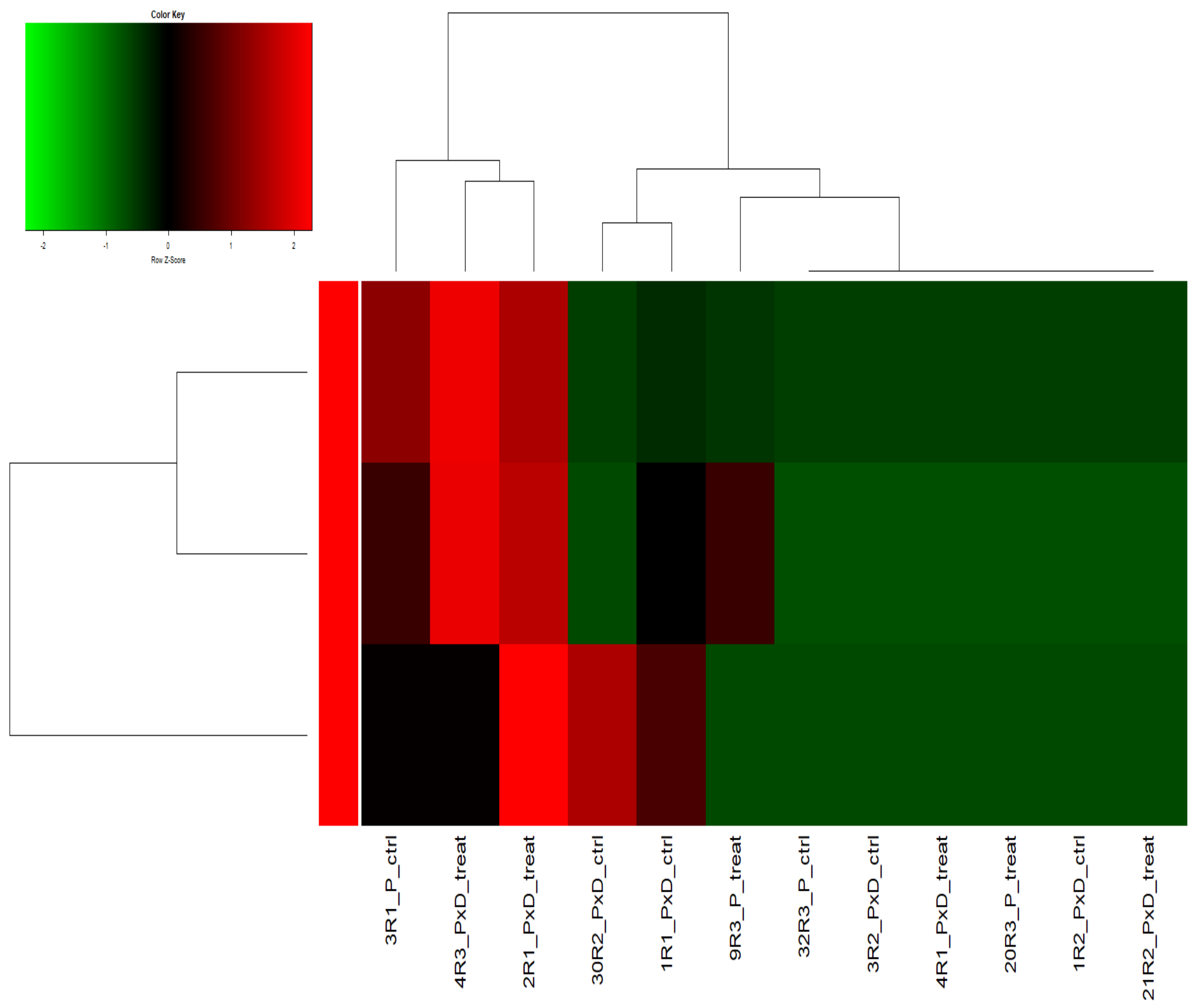

Supp. Figure 12. Expression heatmap of red module. The greener color the lower expression while the redder color the higher expression. Columns represent samples. On top the dendrogram based on euclidian distance of samples miRNA expression profile. On the bottom sample ids. Rows represent miRNAs. On the left, dendrogram based on euclidian distance of miRNAs expression and the color which symbolize the module membership of miRNA.

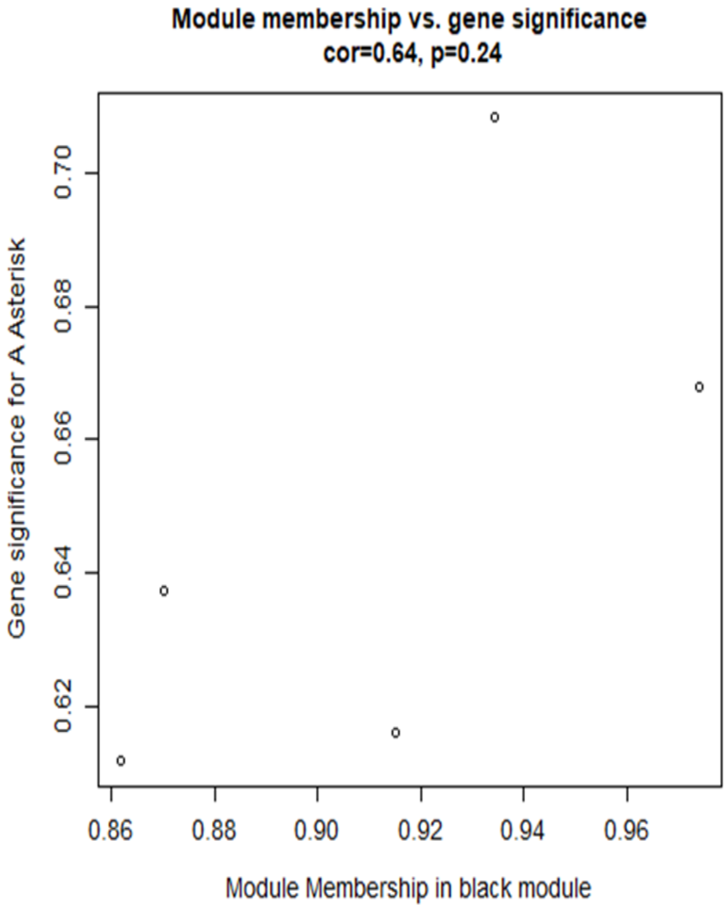

SN	Ensembl	p.GS	GS
1	ssc-miR-142-3p	0.034526086	-0.611739192
2	ssc-miR-204	0.025793242	-0.637352009
3	ssc-miR-30a-3p	0.03292799	-0.616046046
4	ssc-let-7c	0.009938651	-0.708290887
5	ssc-miR-21-5p	0.017595916	-0.667972486

Supp. Figure 13. Intra-modular analysis for MEM (black module). The figure shows the scatter plot of GS (y-axis) vs. MM (x-axis) for phenotypic trait **A asterisk** (a*) in MEM black. The **GS** is the absolute value describing the relationship between the miRNA and the phenotypic trait a*, while the **MM** describes the correlation between the MEM and the miRNA expression profile.

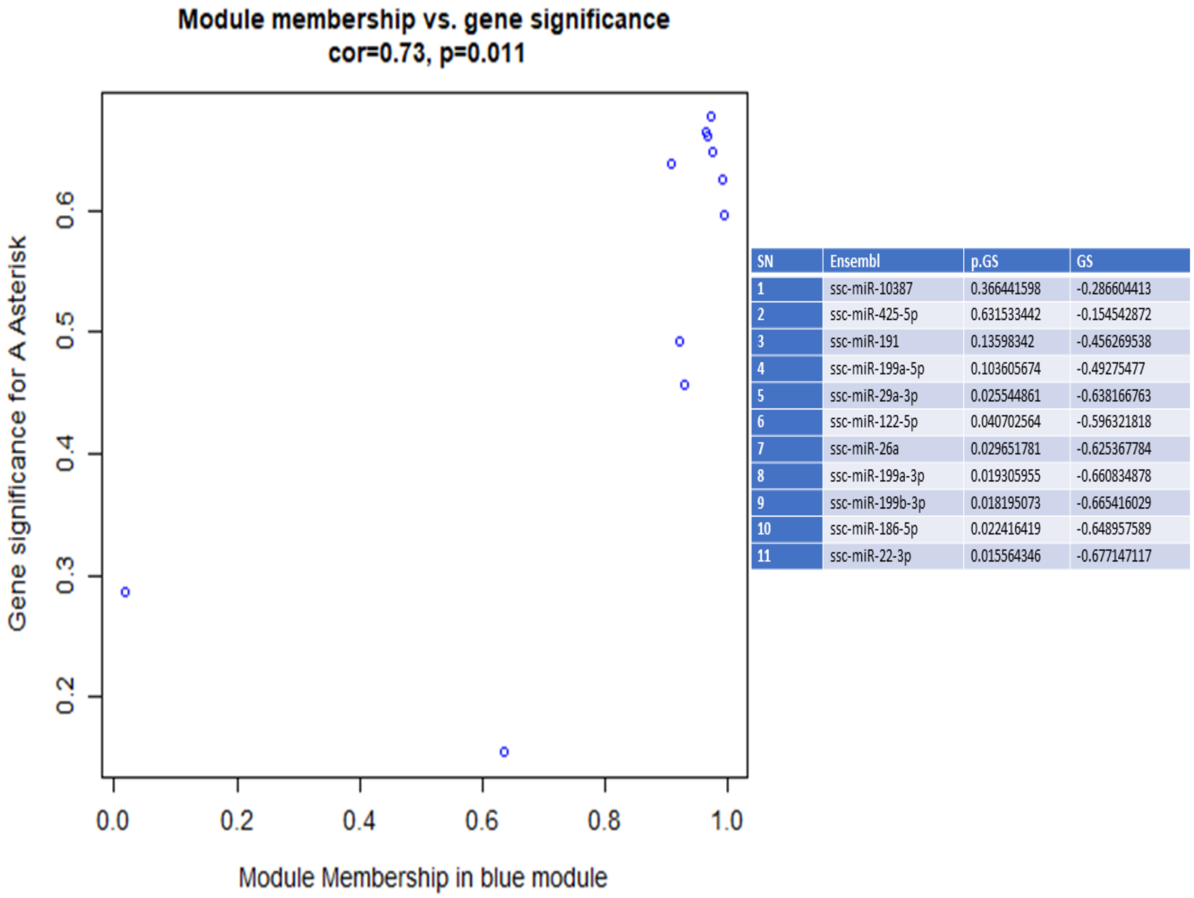

SN	Ensembl	p.GS	GS
1	ssc-miR-10387	0.366441598	-0.286604413
2	ssc-miR-425-5p	0.631533442	-0.154542872
3	ssc-miR-191	0.13598342	-0.456269538
4	ssc-miR-199a-5p	0.103605674	-0.49275477
5	ssc-miR-29a-3p	0.025544861	-0.638166763
6	ssc-miR-122-5p	0.040702564	-0.596321818
7	ssc-miR-26a	0.029651781	-0.625367784
8	ssc-miR-199a-3p	0.019305955	-0.660834878
9	ssc-miR-199b-3p	0.018195073	-0.665416029
10	ssc-miR-186-5p	0.022416419	-0.648957589
11	ssc-miR-22-3p	0.015564346	-0.677147117

Supp. Figure 14. Intra-modular analysis for MEM (blue module). The figure shows the scatter plot of GS (y-axis) vs. MM (x-axis) for phenotypic trait a*in MEM blue. The **GS** is the absolute value describing the relationship between the miRNA and the phenotypic trait a*, while the **MM** describes the correlation between the MEM and the miRNA expression profile.

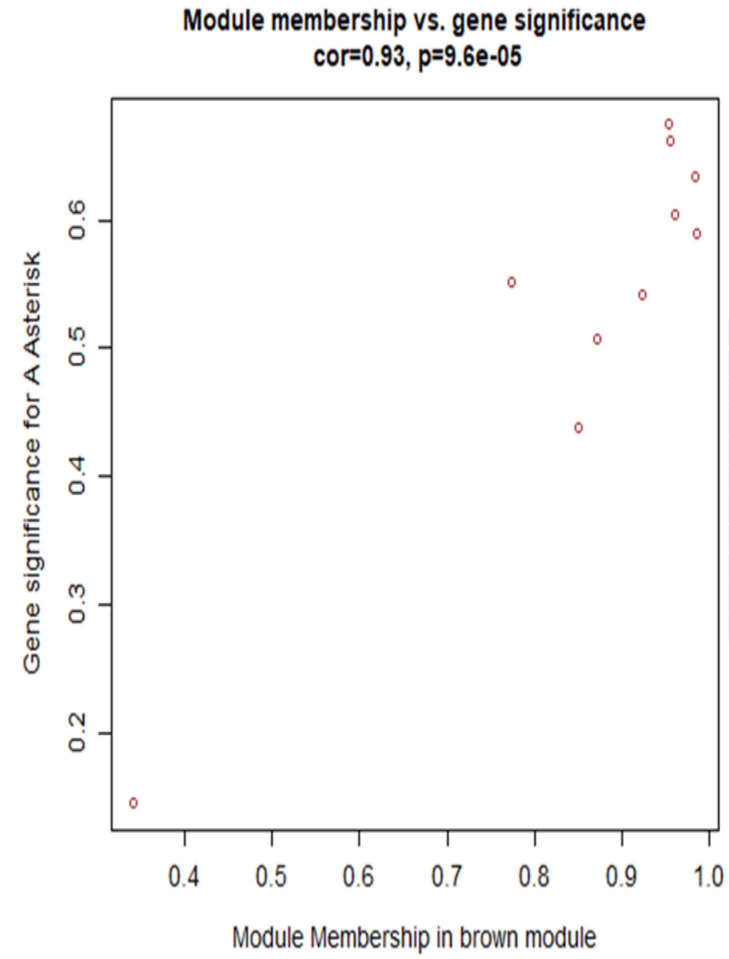

SN	Ensembl	p.GS	GS
1	ssc-miR-125a	0.654101506	-0.144503977
2	ssc-miR-486	0.15477077	-0.437653617
3	ssc-miR-423-3p	0.062632933	-0.55223593
4	ssc-miR-181b	0.092592808	-0.506883309
5	ssc-miR-140-3p	0.069192977	-0.541203943
6	ssc-miR-28-3p	0.043596652	-0.589667089
7	ssc-miR-151-3p	0.037383665	-0.604380864
8	ssc-miR-148a-3p	0.026710103	-0.634392741
9	ssc-miR-26b-5p	0.018943348	-0.662308517
10	ssc-miR-30a-5p	0.016073785	-0.674767401

Supp. Figure 15. Intra-modular analysis for MEM (Brown module). The figure shows the scatter plot of GS (y-axis) vs. MM (x-axis) for phenotypic trait a*in MEM brown. The **GS** is the absolute value describing the relationship between the miRNA and the phenotypic trait a*, while the **MM** describes the correlation between the MEM and the miRNA expression profile.

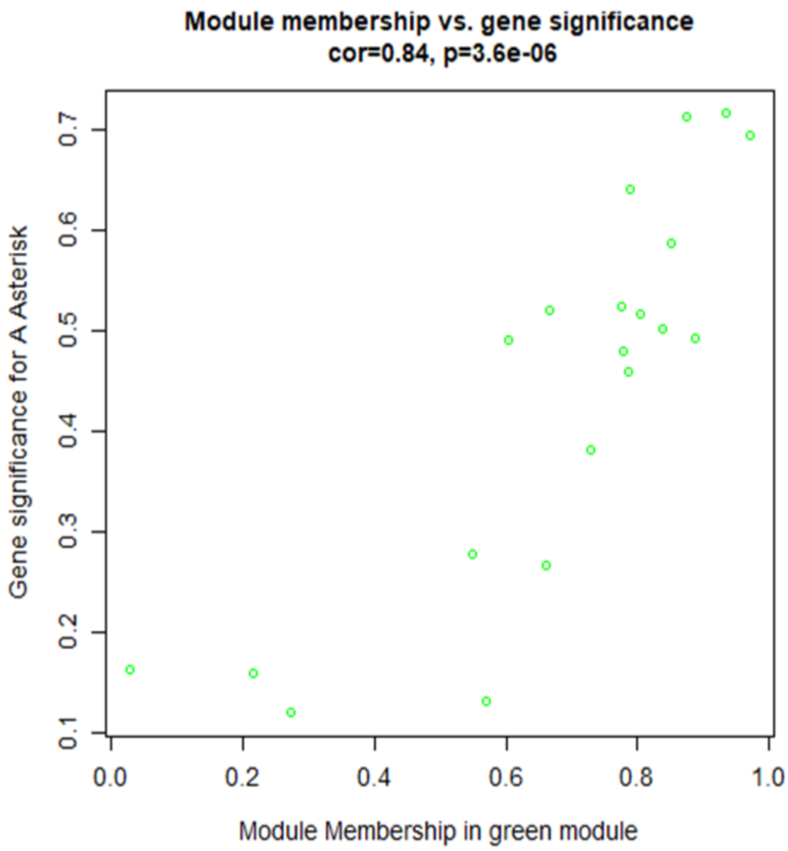

SN	Ensembl	p.GS	GS
1	ssc-miR-7142-3p	0.614398636	0.162250613
2	ssc-miR-127	0.711337401	0.119546706
3	ssc-miR-100	0.686422526	-0.130328818
4	ssc-miR-10b	0.623298587	-0.158237519
5	ssc-miR-374a-5p	0.383695746	-0.276844251
6	ssc-miR-146a-5p	0.40378785	-0.265758905
7	ssc-miR-320	0.221818318	-0.380940931
8	ssc-let-7d-5p	0.079643197	-0.525029918
9	ssc-miR-340	0.085803095	-0.516166579
10	ssc-miR-122-3p	0.095714591	-0.50276876
11	ssc-miR-24-3p	0.104198779	-0.492022994
12	ssc-miR-374a-3p	0.105227753	-0.490759997
13	ssc-miR-7134-3p	0.082484246	-0.520886146
14	ssc-miR-423-5p	0.114019185	-0.48028958
15	ssc-miR-375	0.044230137	-0.588252448
16	ssc-miR-10383	0.133018367	-0.45935647
17	ssc-miR-15a	0.024791945	-0.640672134
18	ssc-miR-107	0.008728864	-0.716652408
19	ssc-miR-148b-3p	0.01221033	-0.694447235
20	ssc-let-7e	0.009240335	-0.713018056

Supp. Figure 16. Intra-modular analysis for MEM (Green module). The figure shows the scatter plot of GS (y-axis) vs. MM (x-axis) for phenotypic trait a*in MEM green. The **GS** is the absolute value describing the relationship between the miRNA and the phenotypic trait a*, while the **MM** describes the correlation between the MEM and the miRNA expression profile.

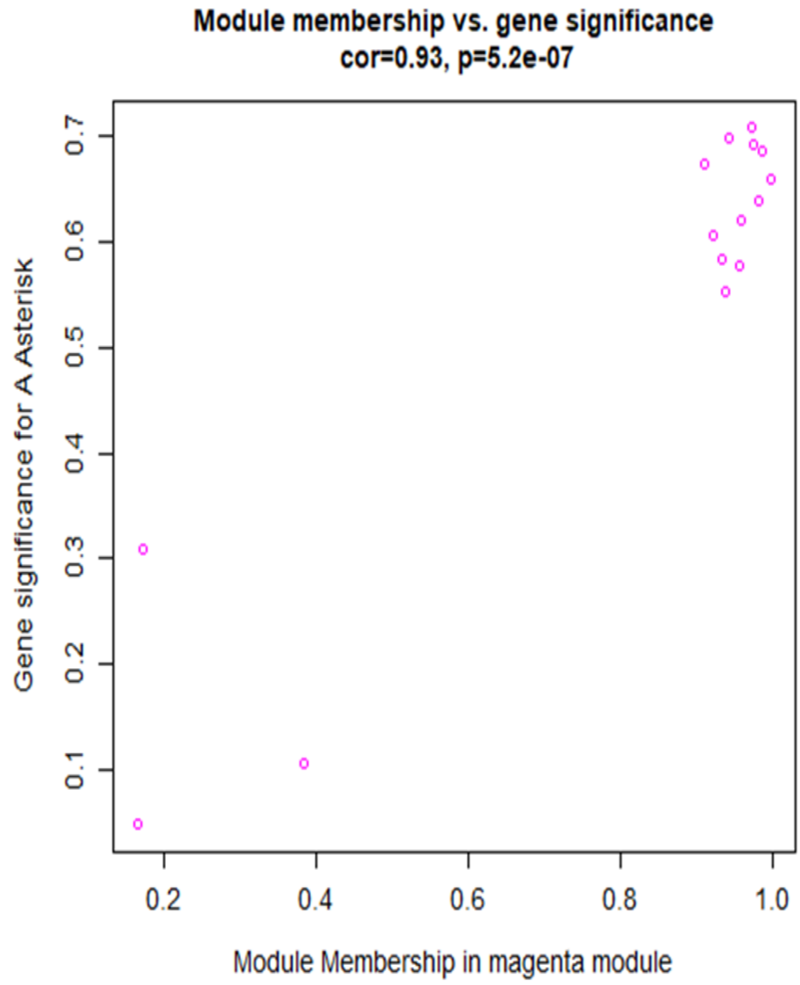

SN	Ensembl	p.GS	GS
1	ssc-miR-10390	0.880393846	-0.04875607
2	ssc-miR-99b	0.742026153	-0.106418063
3	ssc-miR-542-3p	0.32675432	0.310019415
4	ssc-miR-142-5p	0.062229855	-0.552939379
5	ssc-miR-30e-5p	0.037000288	-0.605344209
6	ssc-miR-92a	0.046871237	-0.582504284
7	ssc-miR-103	0.049516065	-0.576972519
8	ssc-let-7i-5p	0.03136874	-0.620395175
9	ssc-miR-222	0.016169428	-0.674326838
10	ssc-miR-192	0.025623568	-0.637907967
11	ssc-miR-98	0.011713458	-0.697300513
12	ssc-let-7a	0.019926706	-0.658358451
13	ssc-miR-101	0.013776206	-0.685983522
14	ssc-miR-221-3p	0.010064819	-0.707463404
15	ssc-miR-30d	0.012588669	-0.692331802

Supp. Figure 17: Intra-modular analysis for MEM (Magenta module). The figure shows the scatter plot of GS (y-axis) vs. MM (x-axis) for phenotypic trait a*in MEM magenta. The **GS** is the absolute value describing the relationship between the miRNA and the phenotypic trait a*, while the **MM** describes the correlation between the MEM and the miRNA expression profile.

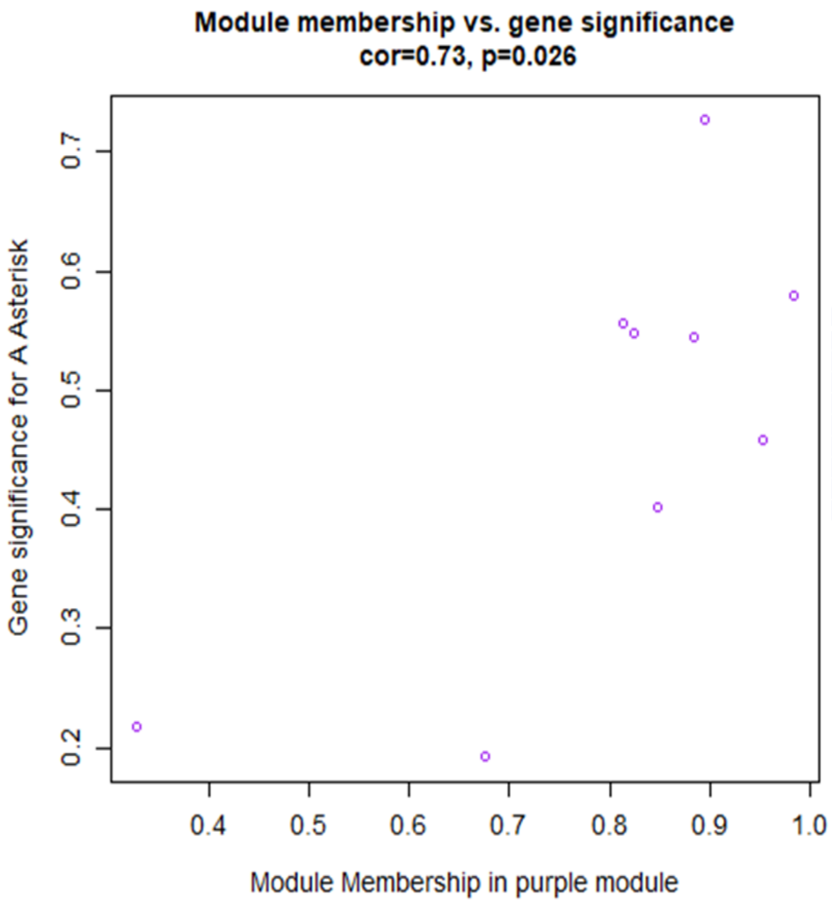

SN	Ensembl	p.GS	GS
1	ssc-miR-125b	0.496423587	-0.217834702
2	ssc-miR-451	0.548872448	-0.192517193
3	ssc-miR-10a-5p	0.195491246	-0.401741017
4	ssc-miR-23a	0.060265579	-0.556413419
5	ssc-miR-23b	0.065248488	-0.547745843
6	ssc-miR-151-5p	0.133536969	-0.458813352
7	ssc-miR-143-3p	0.066693127	-0.545319034
8	ssc-miR-215	0.048169213	-0.579762797
9	ssc-miR-126-3p	0.007461755	-0.726396812

Supp. Figure 18. Intra-modular analysis for MEM (Purple module). The figure shows the scatter plot of GS (y-axis) vs. MM (x-axis) for phenotypic trait a*in MEM purple. The **GS** is the absolute value describing the relationship between the miRNA and the phenotypic trait a*, while the **MM** describes the correlation between the MEM and the miRNA expression profile.

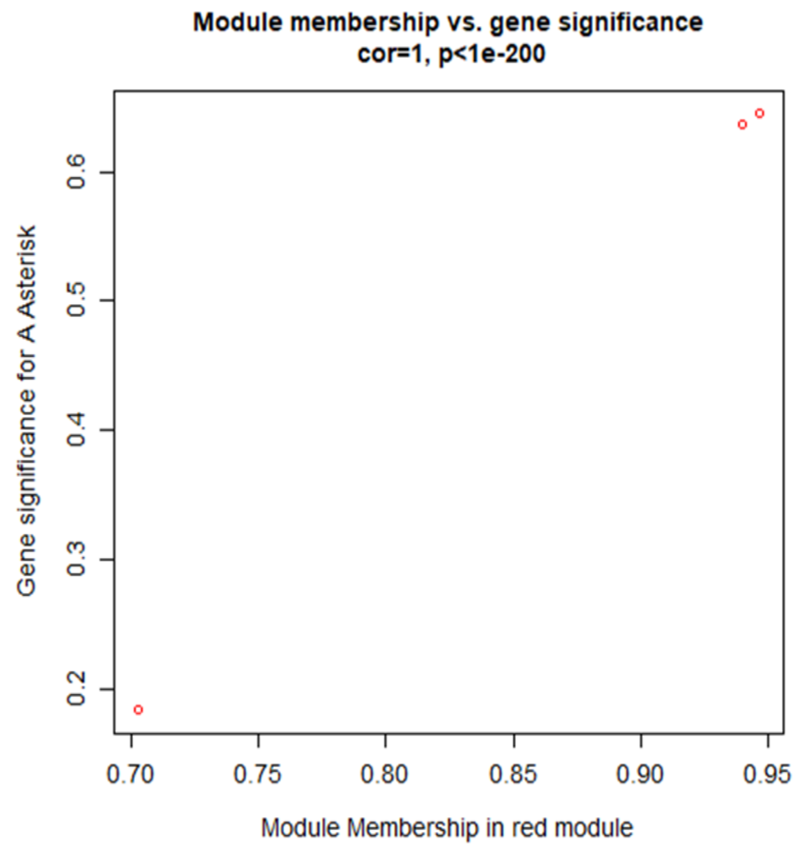

SN	Ensembl	p.GS	GS
1	ssc-miR-27a	0.567574527	-0.183739199
2	ssc-miR-148a-5p	0.023662707	-0.644535284
3	ssc-let-7g	0.02588081	-0.637066116

Supp. Figure 19. Intra-modular analysis for MEM (Red module). The figure shows the scatter plot of GS (y-axis) vs. MM (x-axis) for phenotypic trait a*in MEM red. The **GS** is the absolute value describing the relationship between the miRNA and the phenotypic trait a*, while the **MM** describes the correlation between the MEM and the miRNA expression profile.

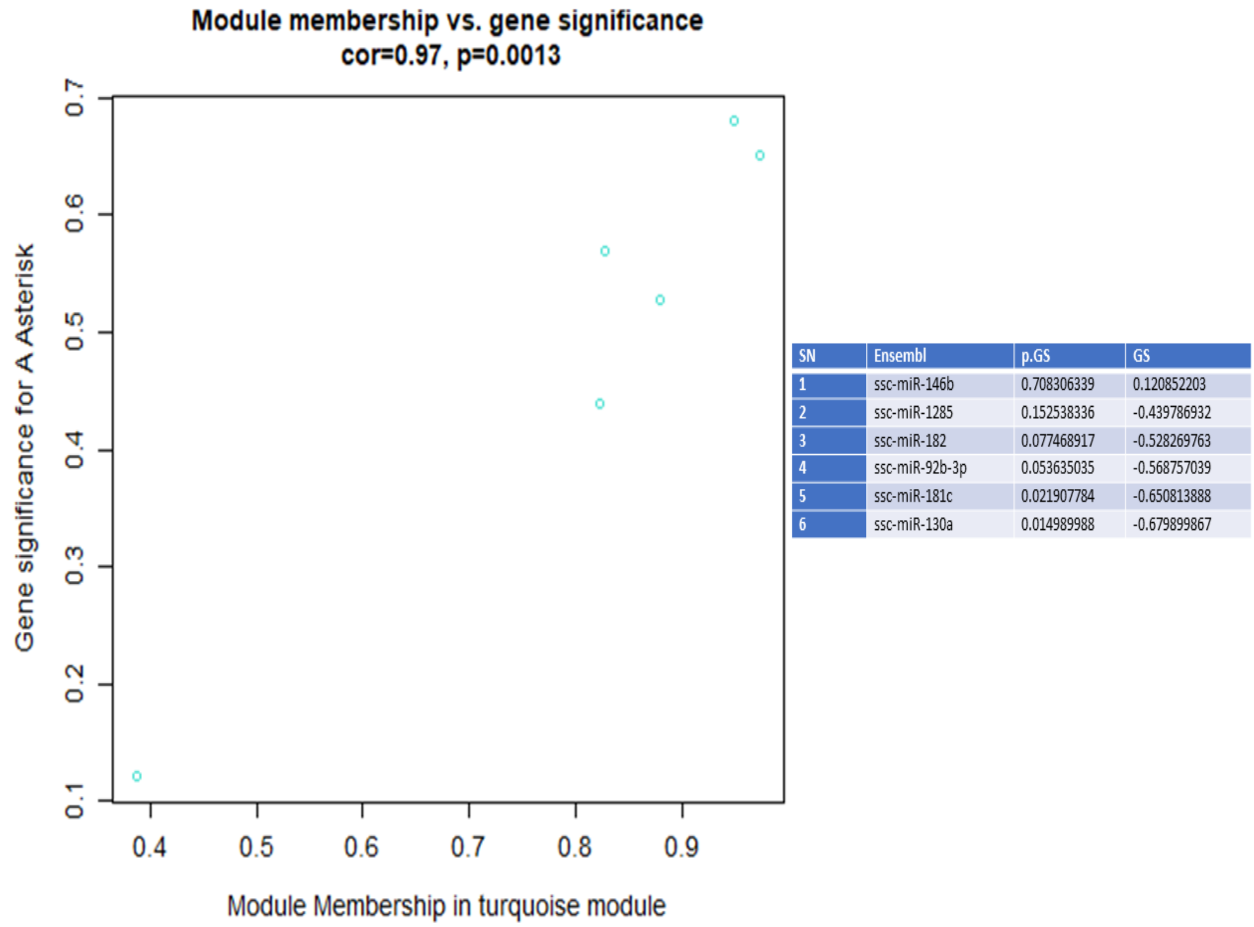

SN	Ensembl	p.GS	GS
1	ssc-miR-146b	0.708306339	0.120852203
2	ssc-miR-1285	0.152538336	-0.439786932
3	ssc-miR-182	0.077468917	-0.528269763
4	ssc-miR-92b-3p	0.053635035	-0.568757039
5	ssc-miR-181c	0.021907784	-0.650813888
6	ssc-miR-130a	0.014989988	-0.679899867

Supp. Figure 20. Intra-modular analysis for MEM (Turquoise module). The figure shows the scatter plot of GS (y-axis) vs. MM (x-axis) for phenotypic trait a*in MEM turquoise. The **GS** is the absolute value describing the relationship between the miRNA and the phenotypic trait a*, while the **MM** describes the correlation between the MEM and the miRNA expression profile.

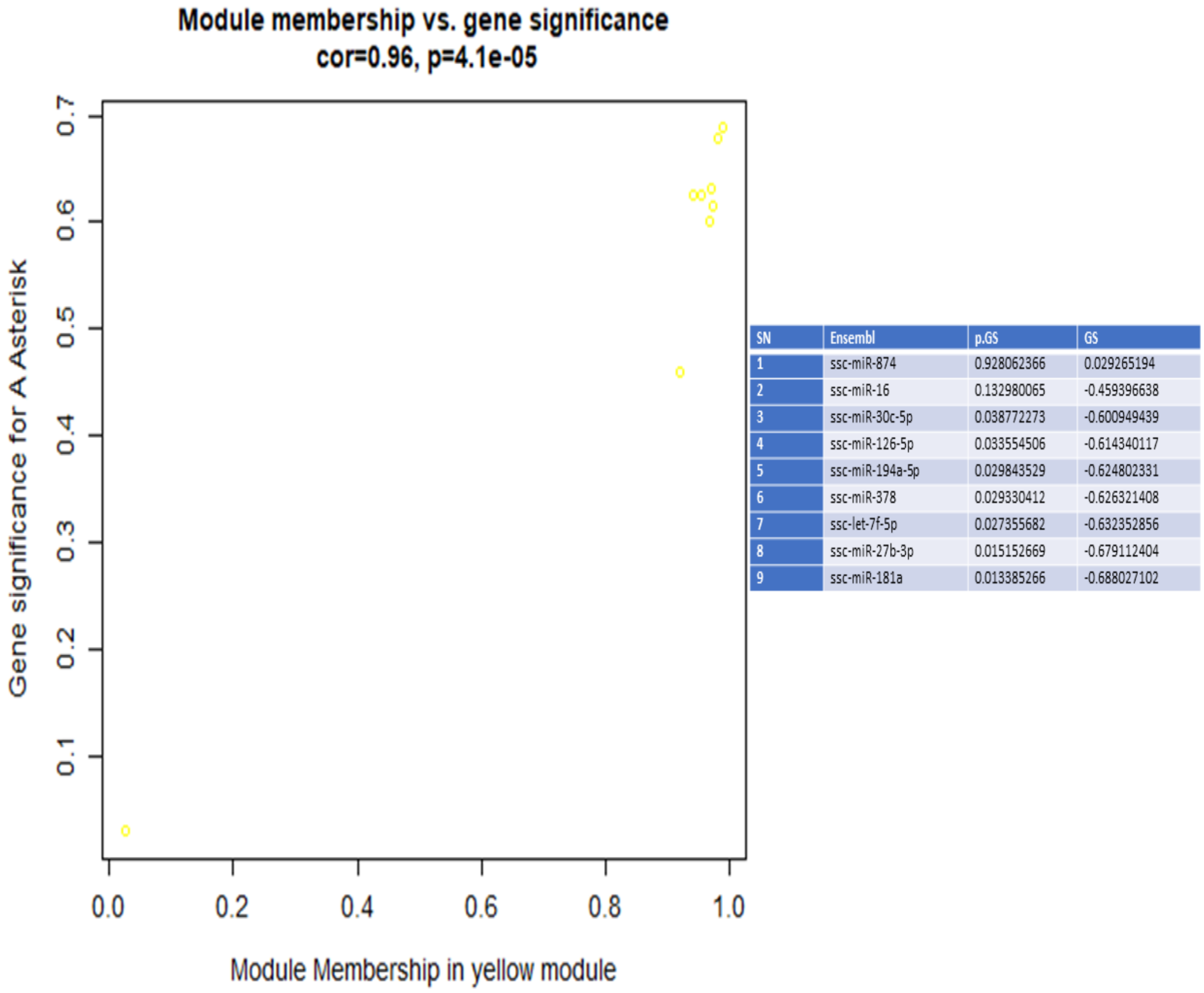

SN	Ensembl	p.GS	GS
1	ssc-miR-874	0.928062366	0.029265194
2	ssc-miR-16	0.132980065	-0.459396638
3	ssc-miR-30c-5p	0.038772273	-0.600949439
4	ssc-miR-126-5p	0.033554506	-0.614340117
5	ssc-miR-194a-5p	0.029843529	-0.624802331
6	ssc-miR-378	0.029330412	-0.626321408
7	ssc-let-7f-5p	0.027355682	-0.632352856
8	ssc-miR-27b-3p	0.015152669	-0.679112404
9	ssc-miR-181a	0.013385266	-0.688027102

Supp. Figure 21. Intra-modular analysis for MEM (Yellow module). The figure shows the scatter plot of GS (y-axis) vs. MM (x-axis) for phenotypic trait a*in MEM yellow. The **GS** is the absolute value describing the relationship between the miRNA and the phenotypic trait a*, while the **MM** describes the correlation between the MEM and the miRNA expression profile.

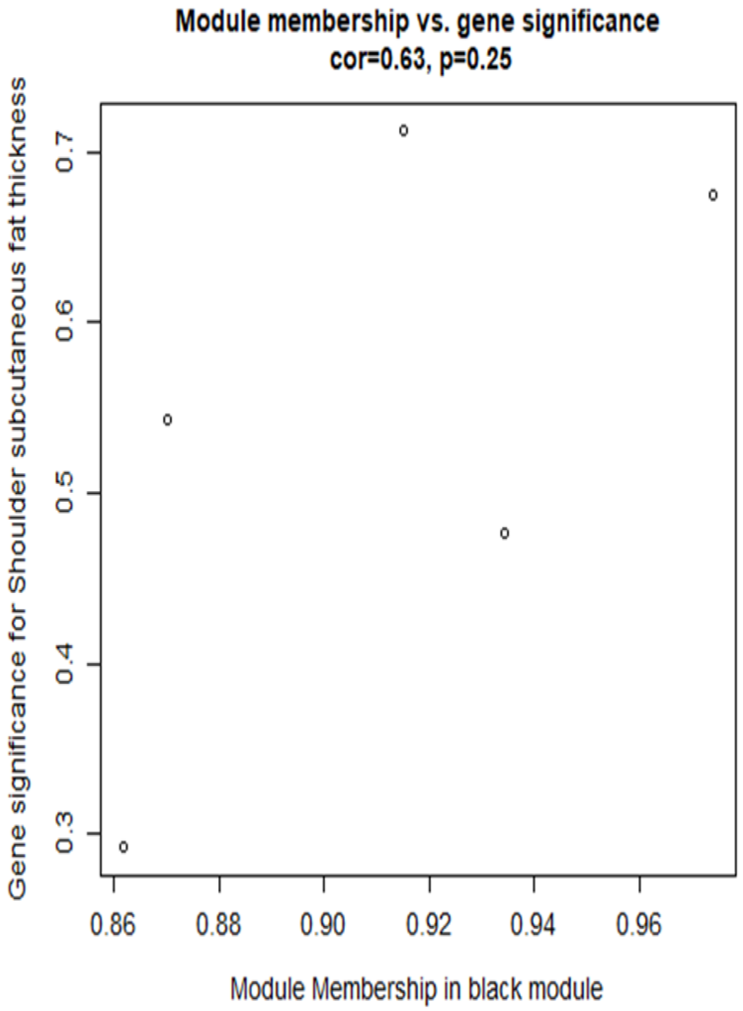

SN	Ensembl	p.GS	GS
1	ssc-miR-30a-3p	0.009365987	-0.712148271
2	ssc-miR-21-5p	0.015933205	-0.675418476
3	ssc-miR-204	0.067966849	-0.543209271
4	ssc-let-7c	0.117054996	-0.476798461
5	ssc-miR-142-3p	0.356226989	-0.292496064

Supp. Figure 22: Intra-modular analysis for MEM (black module). The figure shows the scatter plot of GS (y-axis) vs. MM (x-axis) for phenotypic trait shoulder subcutaneous fat thickness in MEM black. The **GS** is the absolute value describing the relationship between the miRNA and the phenotypic trait shoulder subcutaneous fat thickness, while the **MM** describes the correlation between the MEM and the miRNA expression profile.

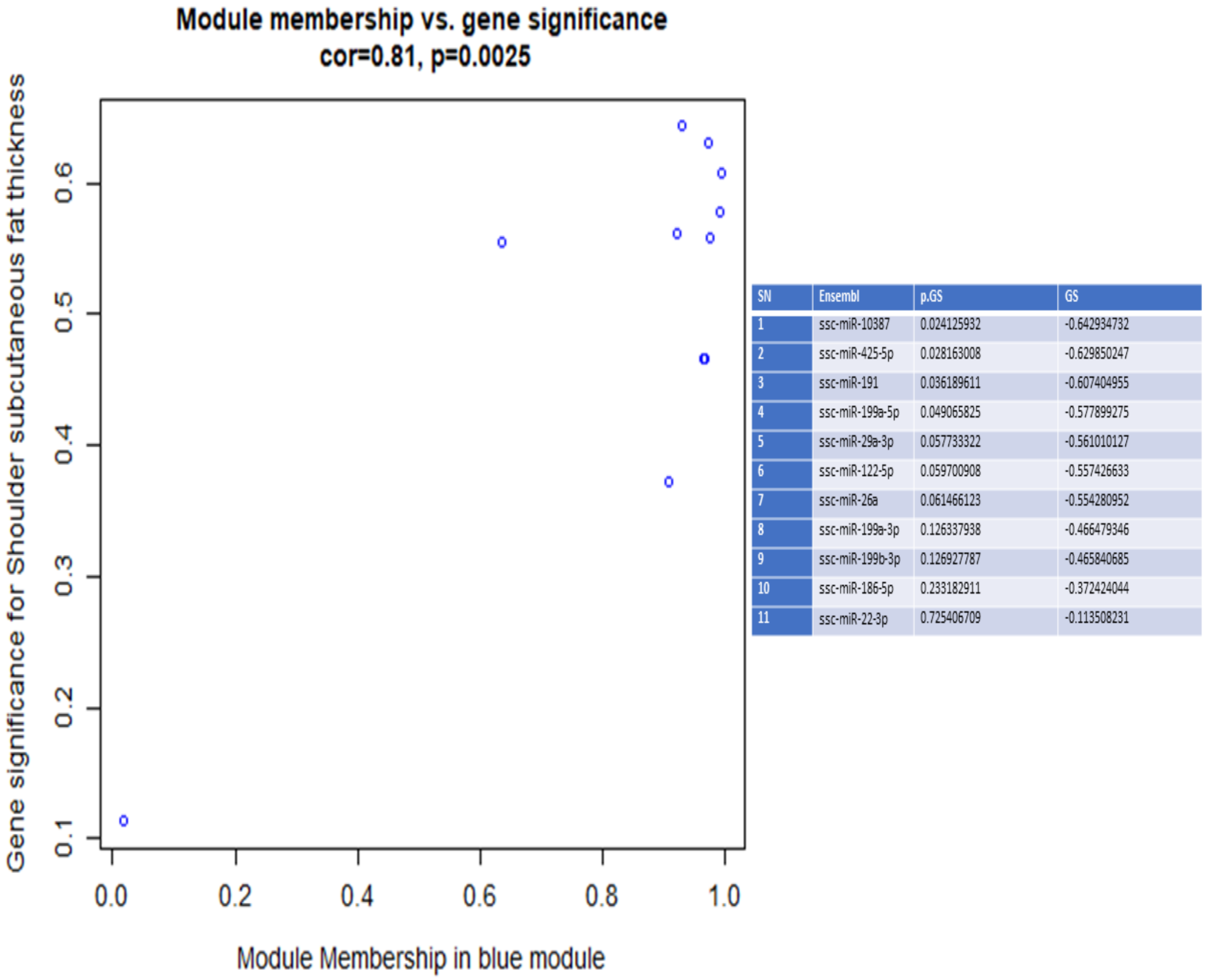

SN	Ensembl	p.GS	GS
1	ssc-miR-10387	0.024125932	-0.642934732
2	ssc-miR-425-5p	0.028163008	-0.629850247
3	ssc-miR-191	0.036189611	-0.607404955
4	ssc-miR-199a-5p	0.049065825	-0.577899275
5	ssc-miR-29a-3p	0.057733322	-0.561010127
6	ssc-miR-122-5p	0.059700908	-0.557426633
7	ssc-miR-26a	0.061466123	-0.554280952
8	ssc-miR-199a-3p	0.126337938	-0.466479346
9	ssc-miR-199b-3p	0.126927787	-0.465840685
10	ssc-miR-186-5p	0.233182911	-0.372424044
11	ssc-miR-22-3p	0.725406709	-0.113508231

Supp. Figure 23: Intra-modular analysis for MEM (blue module). The figure shows the scatter plot of GS (y-axis) vs. MM (x-axis) for phenotypic trait shoulder subcutaneous fat thickness in MEM blue. The **GS** is the absolute value describing the relationship between the miRNA and the phenotypic trait shoulder subcutaneous fat thickness, while the **MM** describes the correlation between the MEM and the miRNA expression profile.

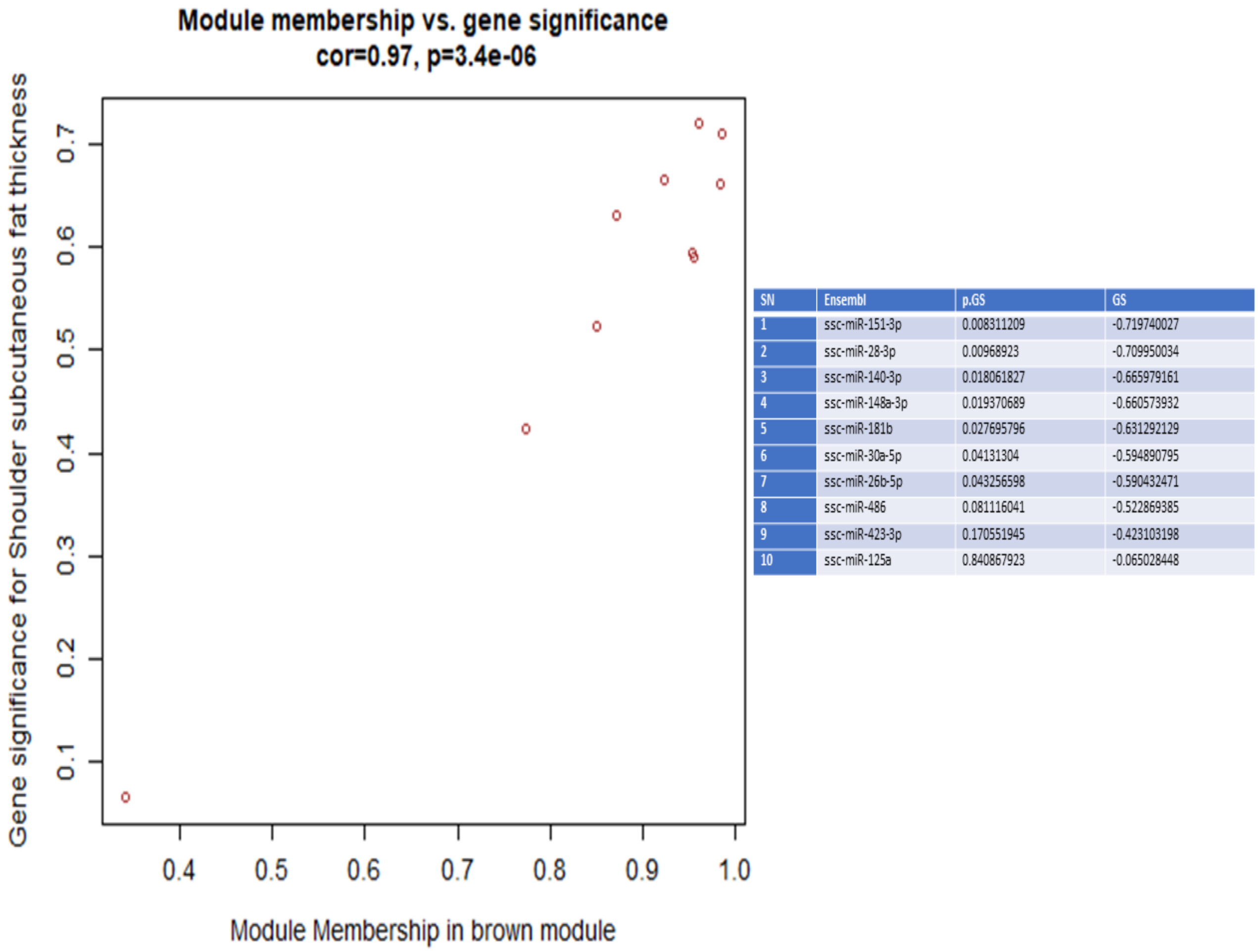

SN	Ensembl	p.GS	GS
1	ssc-miR-151-3p	0.008311209	-0.719740027
2	ssc-miR-28-3p	0.00968923	-0.709950034
3	ssc-miR-140-3p	0.018061827	-0.665979161
4	ssc-miR-148a-3p	0.019370689	-0.660573932
5	ssc-miR-181b	0.027695796	-0.631292129
6	ssc-miR-30a-5p	0.04131304	-0.594890795
7	ssc-miR-26b-5p	0.043256598	-0.590432471
8	ssc-miR-486	0.081116041	-0.522869385
9	ssc-miR-423-3p	0.170551945	-0.423103198
10	ssc-miR-125a	0.840867923	-0.065028448

Supp. Figure 24. Intra-modular analysis for MEM (Brown module). The figure shows the scatter plot of GS (y-axis) vs. MM (x-axis) for phenotypic trait shoulder subcutaneous fat thickness in MEM brown. The **GS** is the absolute value describing the relationship between the miRNA and the phenotypic trait shoulder subcutaneous fat thickness, while the **MM** describes the correlation between the MEM and the miRNA expression profile.

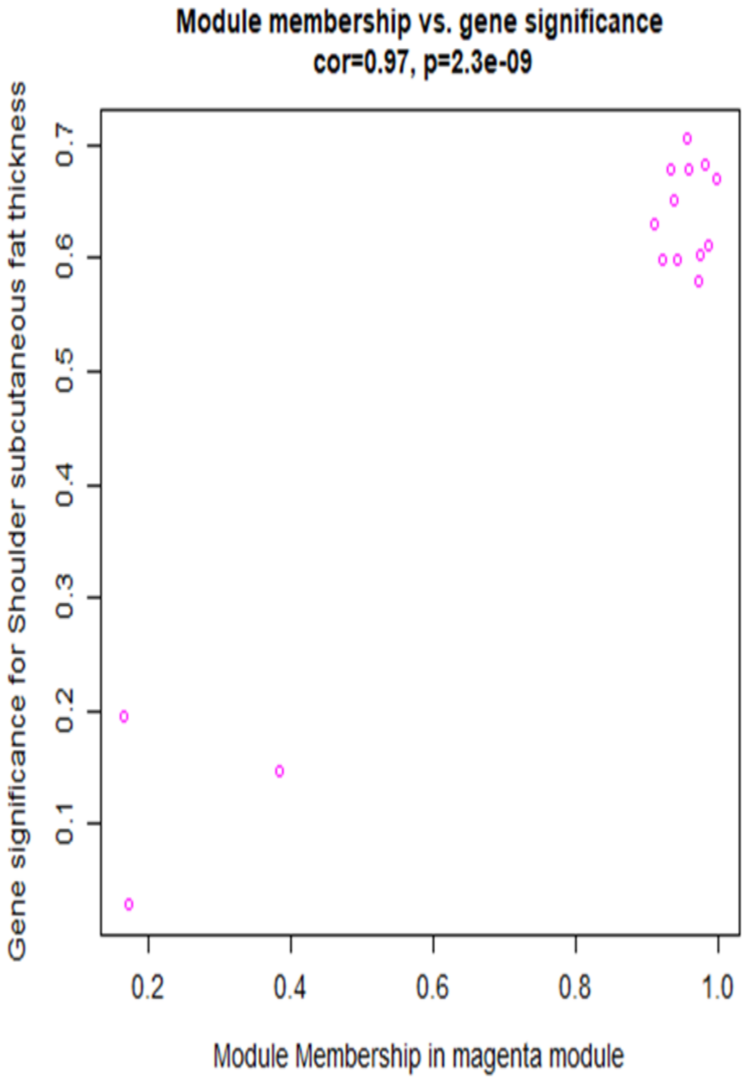

SN	Ensembl	p.GS	GS
1	ssc-miR-103	0.01046956	-0.704859933
2	ssc-miR-192	0.014641211	-0.681609603
3	ssc-let-7i-5p	0.015392268	-0.677963878
4	ssc-miR-92a	0.015420967	-0.677827196
5	ssc-let-7a	0.016981789	-0.670659961
6	ssc-miR-142-5p	0.021907089	-0.650816446
7	ssc-miR-222	0.02839583	-0.629138162
8	ssc-miR-101	0.034942138	-0.610641424
9	ssc-miR-30d	0.038355189	-0.601970778
10	ssc-miR-30e-5p	0.039719499	-0.598658611
11	ssc-miR-98	0.039835542	-0.598380646
12	ssc-miR-221-3p	0.048669306	-0.578720421
13	ssc-miR-10390	0.544802726	0.194443581
14	ssc-miR-99b	0.65027118	-0.146199214
15	ssc-miR-542-3p	0.928677706	-0.0290143

Supp. Figure 25. Intra-modular analysis for MEM (Magenta module). The figure shows the scatter plot of GS (y-axis) vs. MM (x-axis) for phenotypic trait shoulder subcutaneous fat thickness in MEM magenta. The **GS** is the absolute value describing the relationship between the miRNA and the phenotypic trait shoulder subcutaneous fat thickness, while the **MM** describes the correlation between the MEM and the miRNA expression profile.

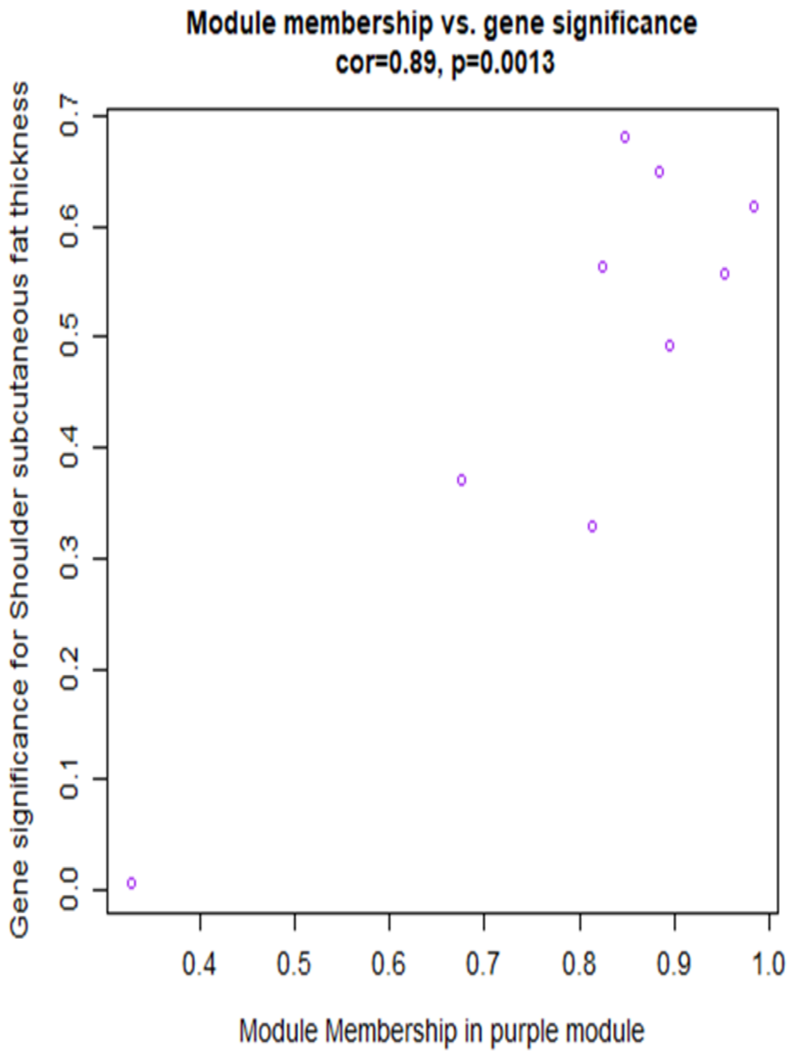

SN	Ensembl	p.GS	GS
1	ssc-miR-10a-5p	0.014990356	-0.679898078
2	ssc-miR-143-3p	0.022445695	-0.648851678
3	ssc-miR-215	0.032376456	-0.617567173
4	ssc-miR-23b	0.05675669	-0.562820495
5	ssc-miR-151-5p	0.060356335	-0.556251187
6	ssc-miR-126-3p	0.103861677	-0.492438571
7	ssc-miR-451	0.236180871	-0.370218815
8	ssc-miR-23a	0.297247491	-0.328450977
9	ssc-miR-125b	0.984406952	0.006336562

Supp. Figure 26. Intra-modular analysis for MEM (Purple). The figure shows the scatter plot of GS (y-axis) vs. MM (x-axis) for phenotypic trait shoulder subcutaneous fat thickness in MEM purple. The **GS** is the absolute value describing the relationship between the miRNA and the phenotypic trait shoulder subcutaneous fat thickness, while the **MM** describes the correlation between the MEM and the miRNA expression profile.

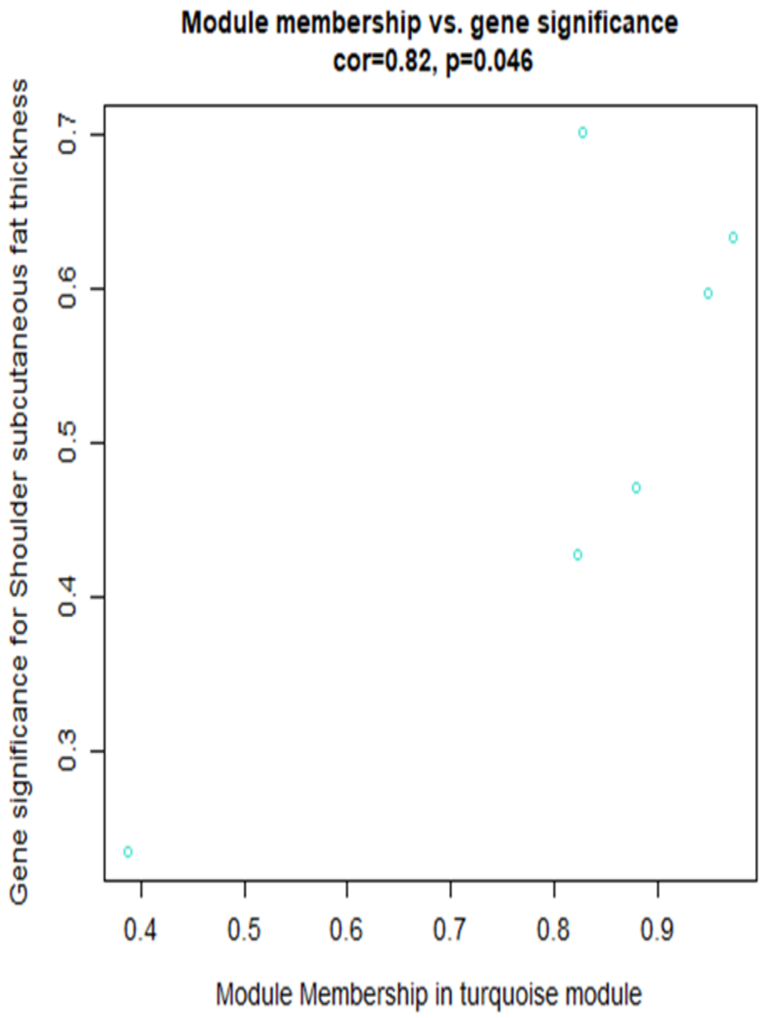

SN	Ensembl	p.GS	GS
1	ssc-miR-146b	0.011046821	-0.701273269
2	ssc-miR-1285	0.026811785	-0.634069101
3	ssc-miR-182	0.040247206	-0.597399182
4	ssc-miR-92b-3p	0.121819035	-0.471438159
5	ssc-miR-181c	0.165368849	-0.427784396
6	ssc-miR-130a	0.464511463	-0.23382345

Supp. Figure 27. Intra-modular analysis for MEM (Turquoise module). The figure shows the scatter plot of GS (y-axis) vs. MM (x-axis) for phenotypic trait shoulder subcutaneous fat thickness in MEM turquoise. The **GS** is the absolute value describing the relationship between the miRNA and the phenotypic trait shoulder subcutaneous fat thickness, while the **MM** describes the correlation between the MEM and the miRNA expression profile.

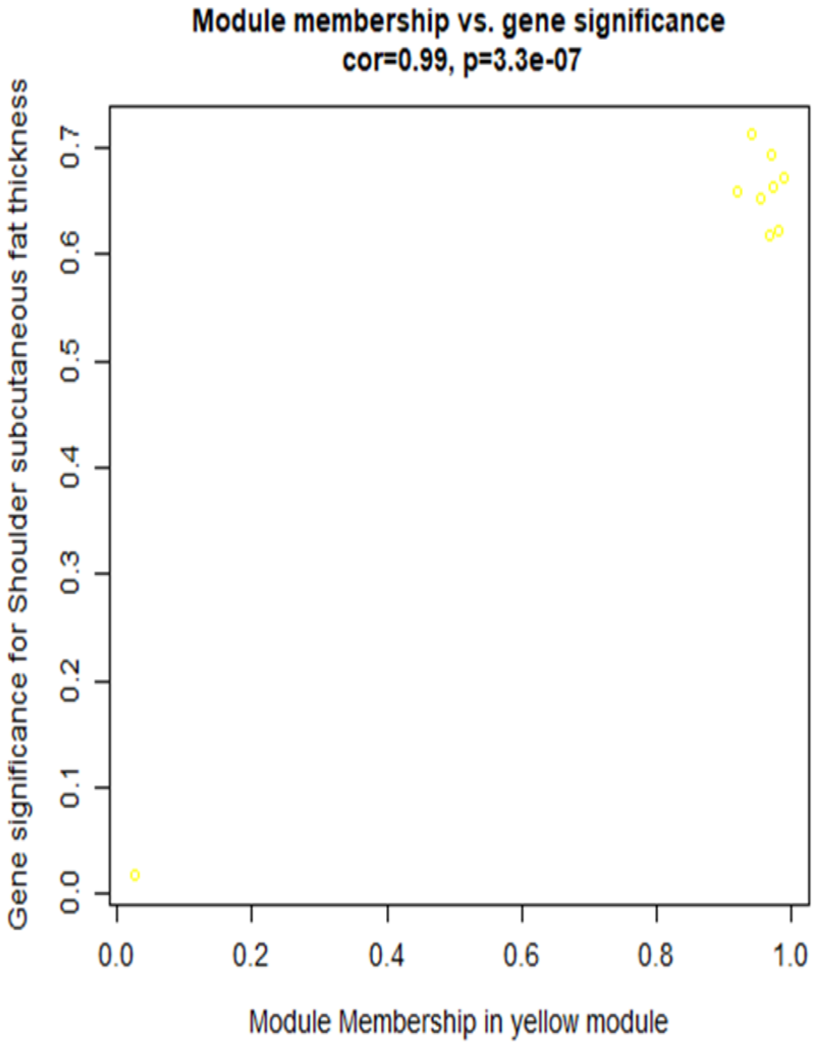

SN	Ensembl	p.GS	GS
1	ssc-miR-378	0.009304947	-0.712569708
2	ssc-let-7f-5p	0.012273438	-0.694091035
3	ssc-miR-181a	0.016768607	-0.67160954
4	ssc-miR-126-5p	0.018575802	-0.663823441
5	ssc-miR-16	0.019645279	-0.659474087
6	ssc-miR-194a-5p	0.021613357	-0.6519028
7	ssc-miR-27b-3p	0.030262221	-0.623576669
8	ssc-miR-30c-5p	0.032373612	-0.617575066
9	ssc-miR-874	0.958103475	-0.017031205

Supp. Figure 28. Intra-modular analysis for MEM (yellow module). The figure shows the scatter plot of GS (y-axis) vs. MM (x-axis) for phenotypic trait shoulder subcutaneous fat thickness in MEM yellow. The **GS** is the absolute value describing the relationship between the miRNA and the phenotypic trait shoulder subcutaneous fat thickness, while the **MM** describes the correlation between the MEM and the miRNA expression profile.

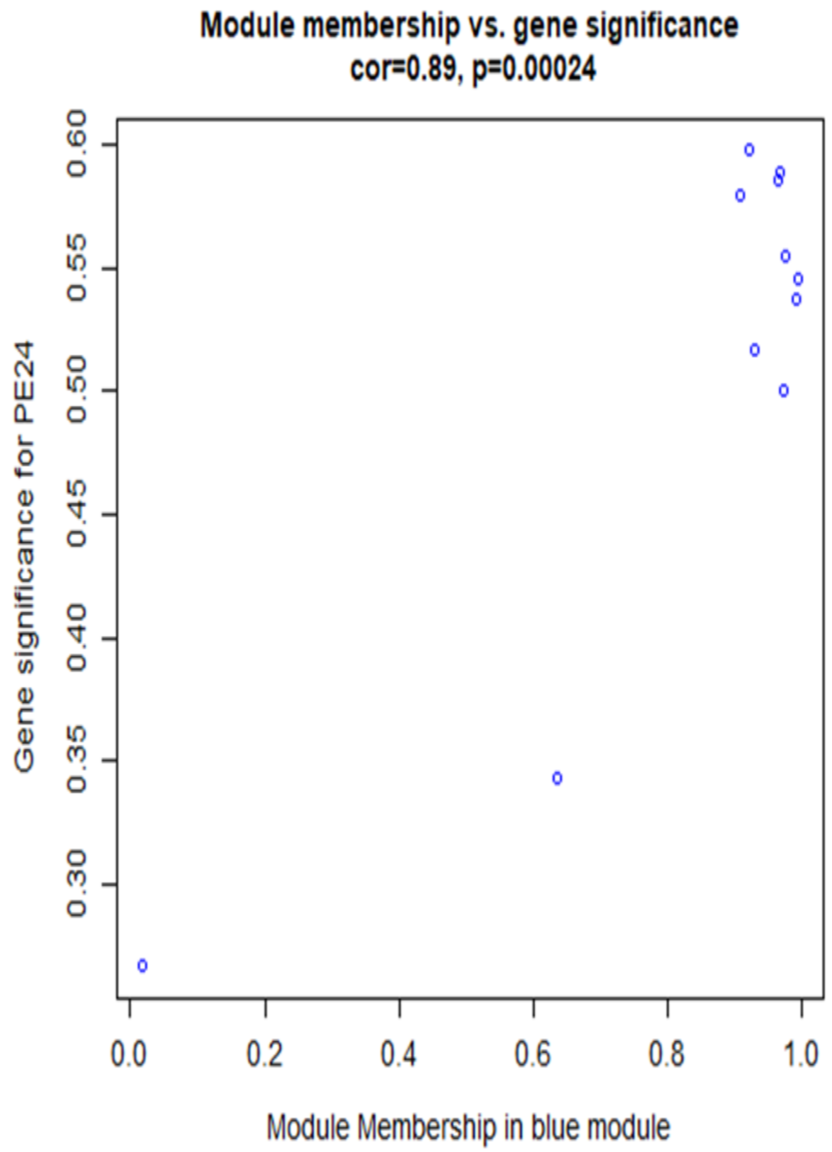

SN	Ensembl	p.GS	GS
1	ssc-miR-199a-5p	0.040219059	-0.597466061
2	ssc-miR-199a-3p	0.043915251	-0.588953817
3	ssc-miR-199b-3p	0.045342948	-0.585801726
4	ssc-miR-29a-3p	0.048354014	-0.579376721
5	ssc-miR-186-5p	0.061212417	-0.55472917
6	ssc-miR-122-5p	0.066485011	-0.545666388
7	ssc-miR-26a	0.071633127	-0.537284733
8	ssc-miR-191	0.085103763	-0.517150625
9	ssc-miR-22-3p	0.097325726	-0.500680466
10	ssc-miR-425-5p	0.274192548	-0.343579417
11	ssc-miR-10387	0.401664631	-0.266916914

Supp. Figure 29. Intra-modular analysis for MEM (blue module). The figure shows the scatter plot of GS (y-axis) vs. MM (x-axis) for phenotypic trait Conductivity 24 hours postmortem (PE24) in MEM blue. The **GS** is the absolute value describing the relationship between the miRNA and the phenotypic trait Conductivity 24 hours postmortem (PE24), while the **MM** describes the correlation between the MEM and the miRNA expression profile.

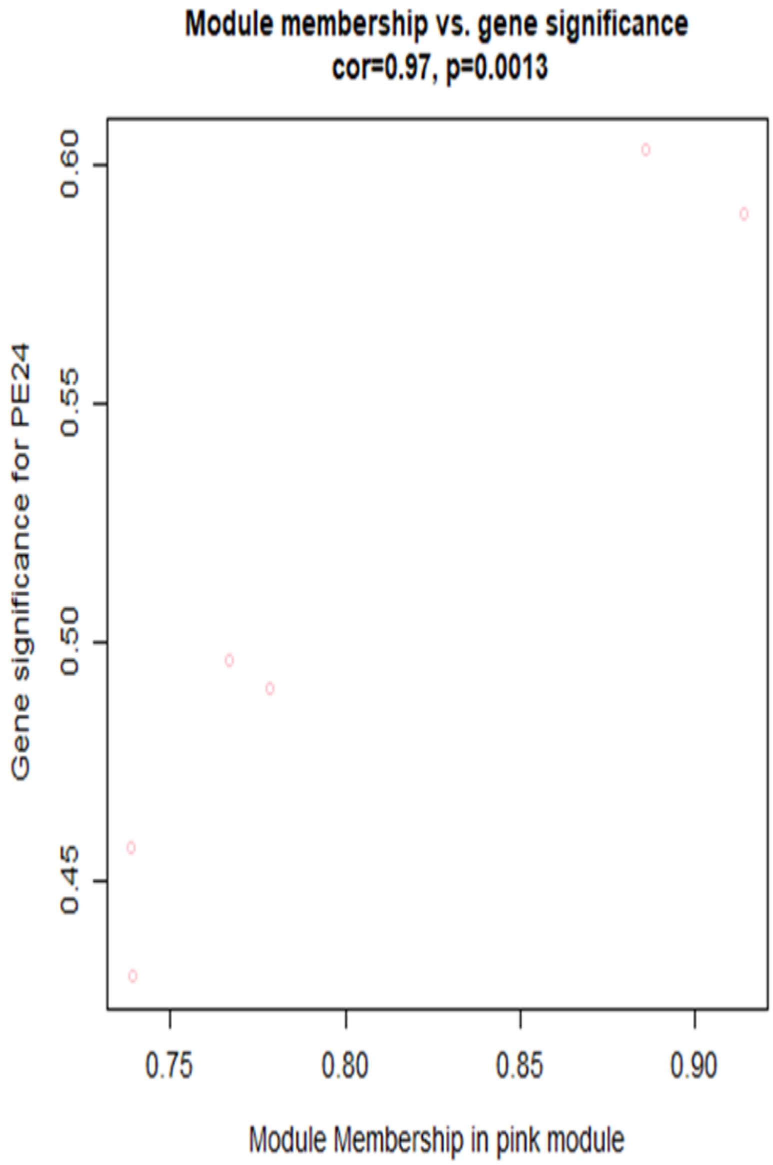

SN	Ensembl	p.GS	GS
1	ssc-miR-30b-5p	0.081547887	-0.522240974
2	ssc-miR-30e-3p	0.138630005	-0.453550799
3	ssc-miR-99a-5p	0.191080269	-0.405390679
4	ssc-miR-19b	0.261705572	-0.35207853
5	ssc-miR-339	0.766216014	0.096174188
6	ssc-miR-339-5p	0.816095063	0.075295913

Supp. Figure 30. Intra-modular analysis for MEM (Pink module). The figure shows the scatter plot of GS (y-axis) vs. MM (x-axis) for phenotypic trait Conductivity 24 hours postmortem (PE24) in MEM pink. The **GS** is the absolute value describing the relationship between the miRNA and the phenotypic trait Conductivity 24 hours postmortem (PE24), while the **MM** describes the correlation between the MEM and the miRNA expression profile.

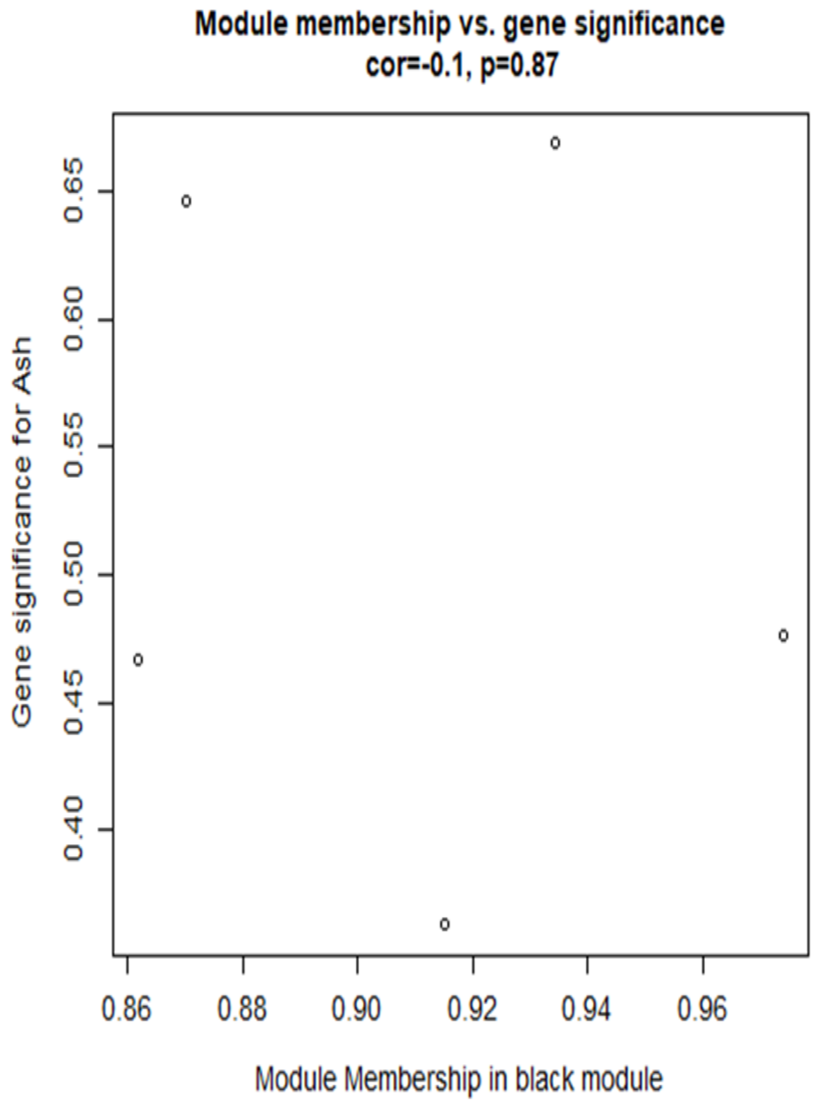

SN	Ensembl	p.GS	GS
1	ssc-let-7c	0.124625278	-0.468344851
2	ssc-miR-204	0.245931885	-0.363158849
3	ssc-miR-21-5p	0.272858221	-0.344476819
4	ssc-miR-30a-3p	0.33125619	-0.307289105
5	ssc-miR-142-3p	0.346512992	-0.298182567

Supp. Figure 31. Intra-modular analysis for MEM (Black module). The figure shows the scatter plot of GS (y-axis) vs. MM (x-axis) for phenotypic trait ashes in MEM black. The **GS** is the absolute value describing the relationship between the miRNA and the phenotypic trait ashes.

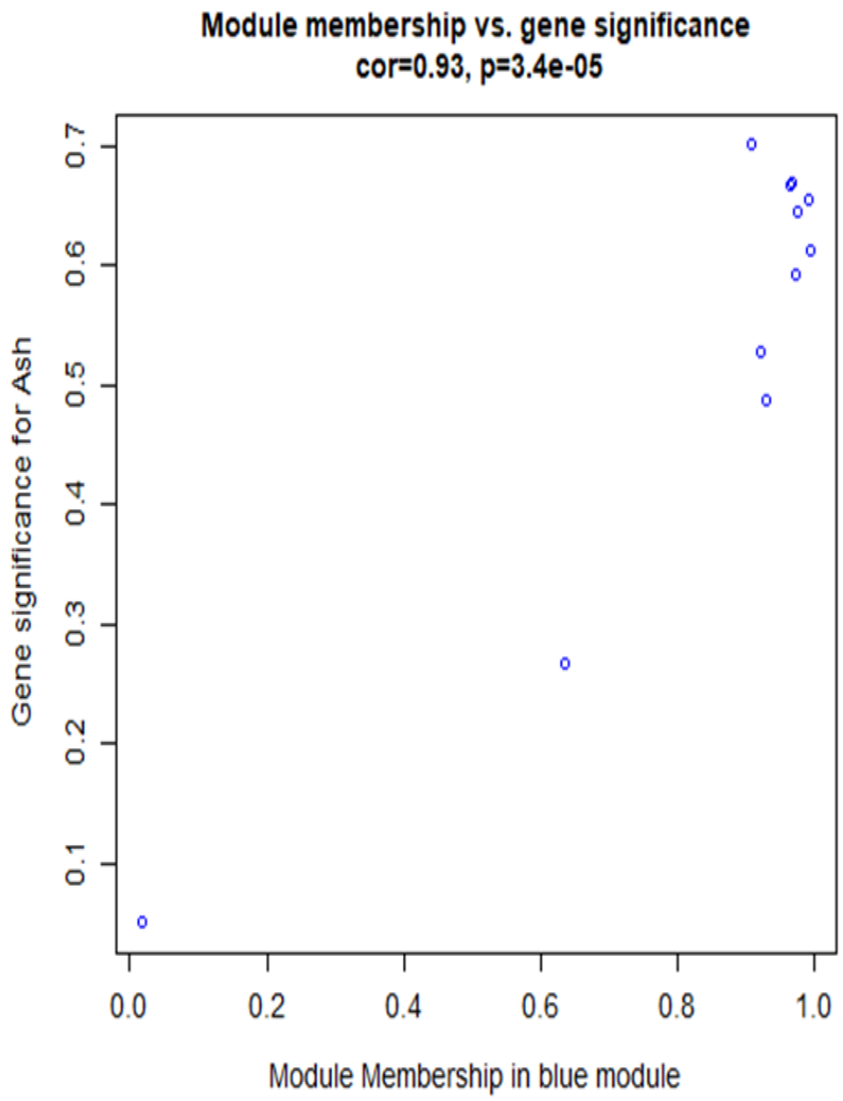

SN	Ensembl	p.GS	GS
1	ssc-miR-199a-3p	0.017373041	0.668939729
2	ssc-miR-199b-3p	0.017728166	0.667402779
3	ssc-miR-26a	0.020928264	0.654479058
4	ssc-miR-186-5p	0.02363066	0.644646854
5	ssc-miR-122-5p	0.034109896	0.612846836
6	ssc-miR-22-3p	0.042599447	0.591923728
7	ssc-miR-199a-5p	0.078032064	0.527424734
8	ssc-miR-191	0.107693913	0.487766048
9	ssc-miR-425-5p	0.402040762	0.266711546
10	ssc-miR-10387	0.877092617	0.050110715

Supp. Figure 32. Intra-modular analysis for MEM (Blue module). The figure shows the scatter plot of GS (y-axis) vs. MM (x-axis) for phenotypic trait ashes in MEM blue. The **GS** is the absolute value describing the relationship between the miRNA and the phenotypic trait ashes.

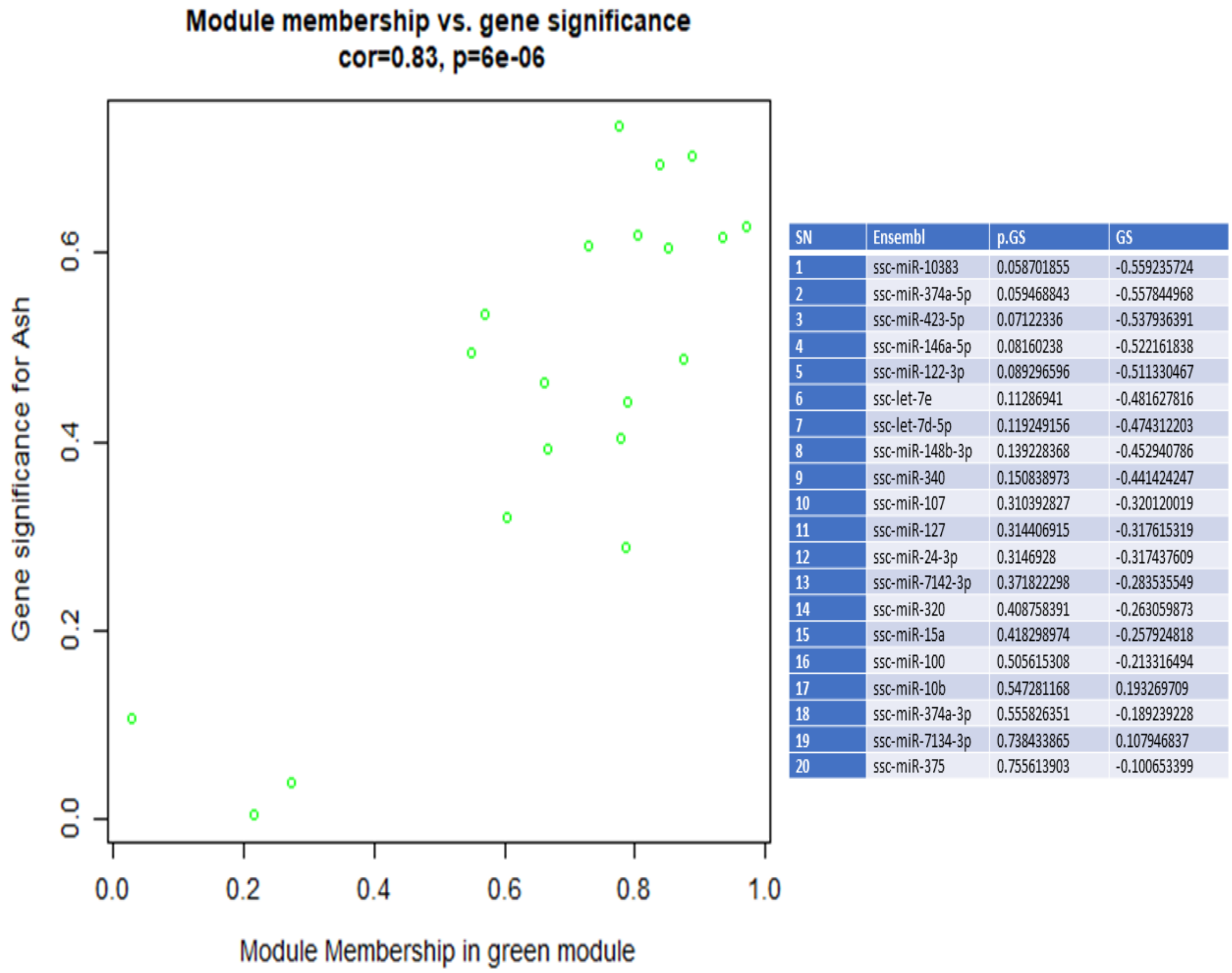

SN	Ensembl	p.GS	GS
1	ssc-miR-10383	0.058701855	-0.559235724
2	ssc-miR-374a-5p	0.059468843	-0.557844968
3	ssc-miR-423-5p	0.07122336	-0.537936391
4	ssc-miR-146a-5p	0.08160238	-0.522161838
5	ssc-miR-122-3p	0.089296596	-0.511330467
6	ssc-let-7e	0.11286941	-0.481627816
7	ssc-let-7d-5p	0.119249156	-0.474312203
8	ssc-miR-148b-3p	0.139228368	-0.452940786
9	ssc-miR-340	0.150838973	-0.441424247
10	ssc-miR-107	0.310392827	-0.320120019
11	ssc-miR-127	0.314406915	-0.317615319
12	ssc-miR-24-3p	0.3146928	-0.317437609
13	ssc-miR-7142-3p	0.371822298	-0.283535549
14	ssc-miR-320	0.408758391	-0.263059873
15	ssc-miR-15a	0.418298974	-0.257924818
16	ssc-miR-100	0.505615308	-0.213316494
17	ssc-miR-10b	0.547281168	0.193269709
18	ssc-miR-374a-3p	0.555826351	-0.189239228
19	ssc-miR-7134-3p	0.738433865	0.107946837
20	ssc-miR-375	0.755613903	-0.100653399

Supp. Figure 33. Intra-modular analysis for MEM (Green module). The figure shows the scatter plot of GS (y-axis) vs. MM (x-axis) for phenotypic trait ashes in MEM green. The **GS** is the absolute value describing the relationship between the miRNA and the phenotypic trait ashes.

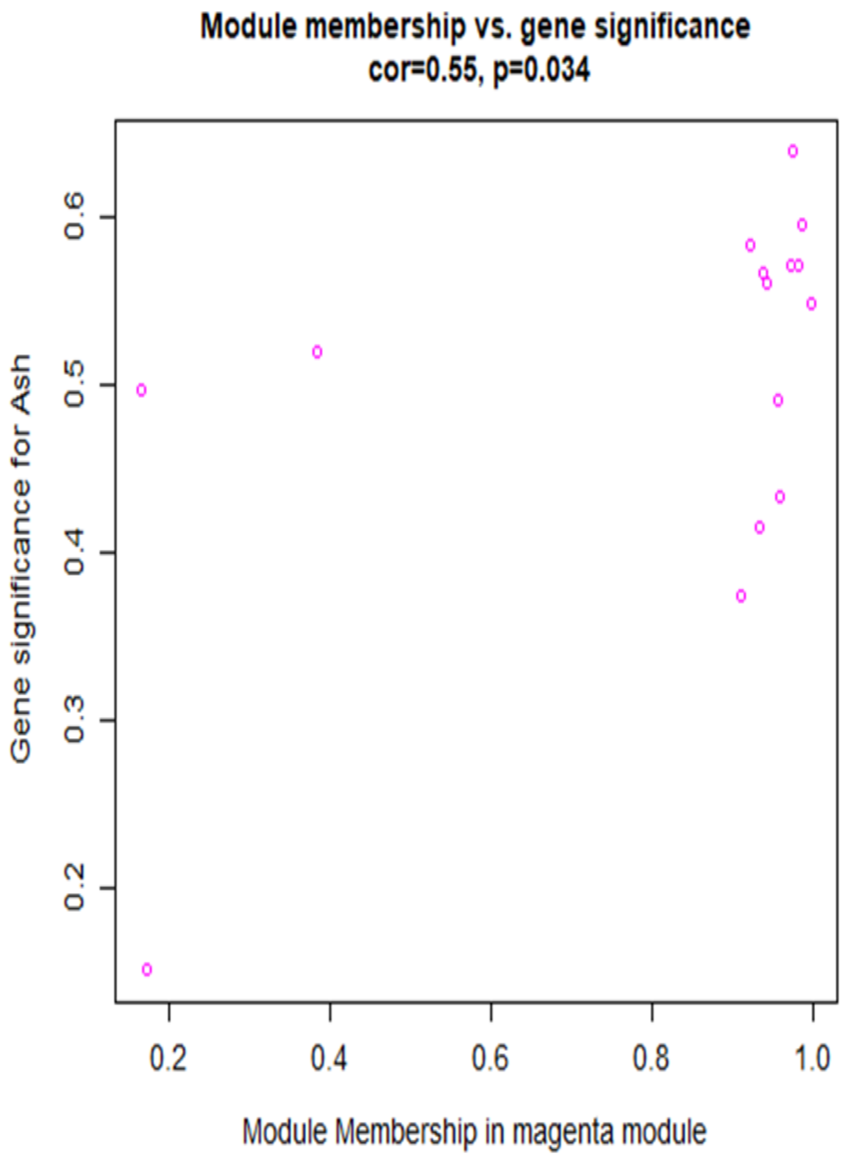

SN	Ensembl	p.GS	GS
1	ssc-miR-142-5p	0.086033597	-0.515843426
2	ssc-miR-30e-5p	0.09087084	-0.509192971
3	ssc-miR-10390	0.13018842	-0.462344722
4	ssc-let-7a	0.16450193	-0.428576367
5	ssc-miR-99b	0.166842006	-0.42644459
6	ssc-miR-192	0.171506997	-0.422250441
7	ssc-miR-101	0.183093322	-0.412134086
8	ssc-miR-30d	0.195980752	-0.401339138
9	ssc-miR-221-3p	0.267751945	-0.347934572
10	ssc-miR-98	0.290680057	-0.332690581
11	ssc-let-7i-5p	0.293257283	-0.331020483
12	ssc-miR-222	0.397045525	-0.269446905
13	ssc-miR-92a	0.408904668	-0.262980693
14	ssc-miR-103	0.428506575	-0.252494509
15	ssc-miR-542-3p	0.526053759	0.203397681

Supp. Figure 34. Intra-modular analysis for MEM (Magenta module). The figure shows the scatter plot of GS (y-axis) vs. MM (x-axis) for phenotypic trait ashes in MEM magenta. The **GS** is the absolute value describing the relationship between the miRNA and the phenotypic trait ashes.

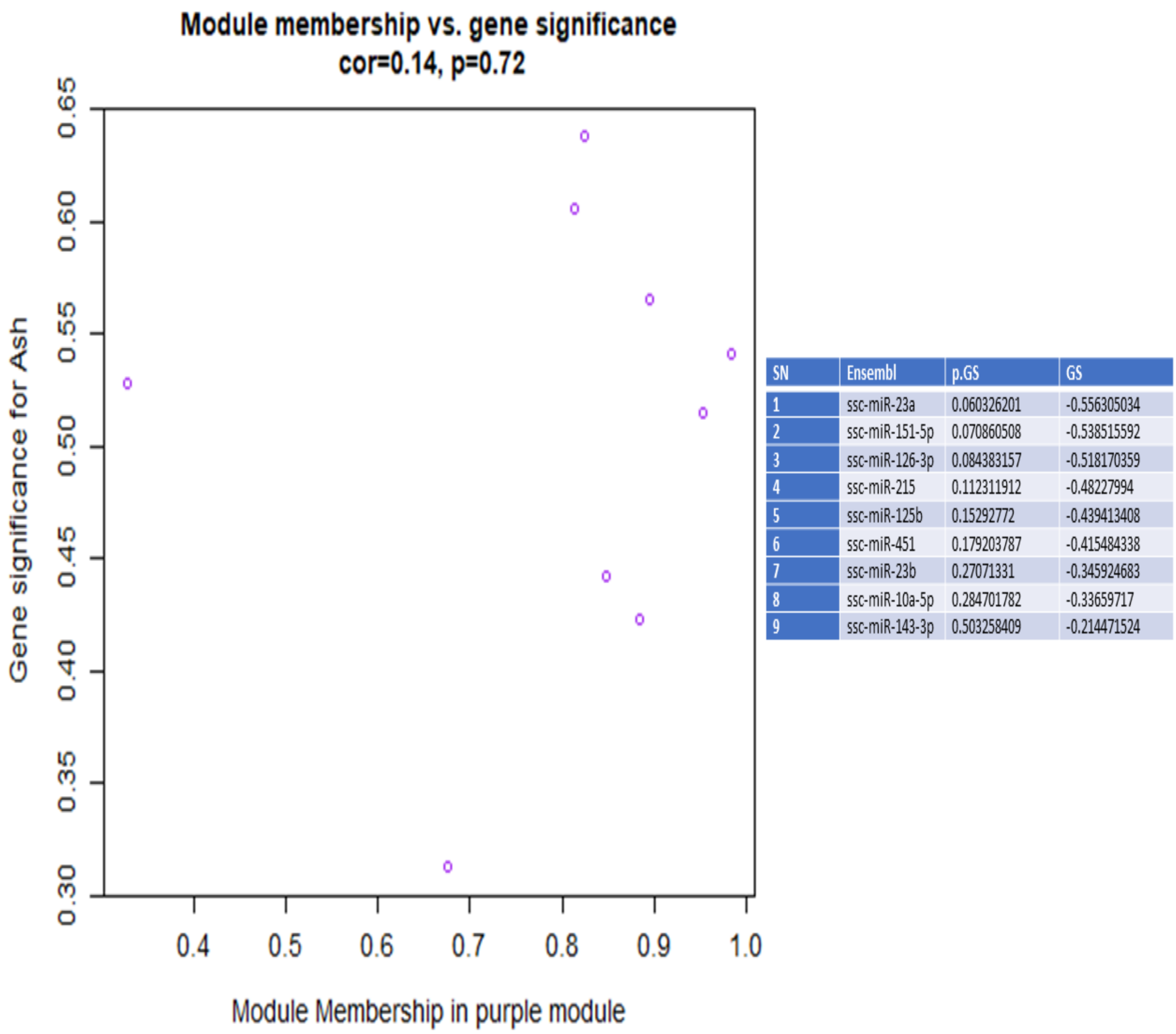

SN	Ensembl	p.GS	GS
1	ssc-miR-23a	0.060326201	-0.556305034
2	ssc-miR-151-5p	0.070860508	-0.538515592
3	ssc-miR-126-3p	0.084383157	-0.518170359
4	ssc-miR-215	0.112311912	-0.48227994
5	ssc-miR-125b	0.15292772	-0.439413408
6	ssc-miR-451	0.179203787	-0.415484338
7	ssc-miR-23b	0.27071331	-0.345924683
8	ssc-miR-10a-5p	0.284701782	-0.33659717
9	ssc-miR-143-3p	0.503258409	-0.214471524

Supp. Figure 35. Intra-modular analysis for MEM (Purple module). The figure shows the scatter plot of GS (y-axis) vs. MM (x-axis) for phenotypic trait ashes in MEM purple. The **GS** is the absolute value describing the relationship between the miRNA and the phenotypic trait ashes.

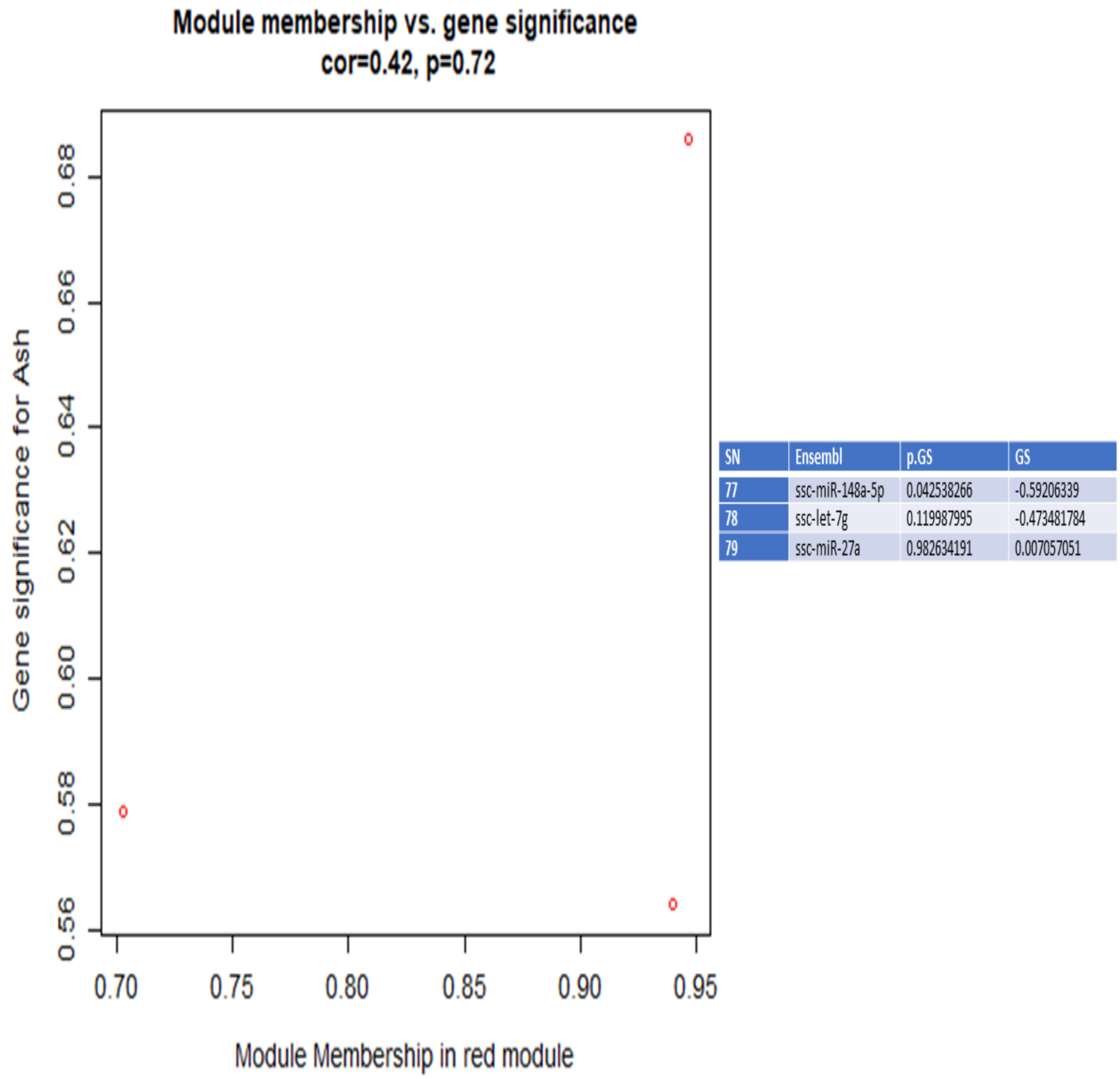

SN	Ensembl	p.GS	GS
77	ssc-miR-148a-5p	0.042538266	-0.59206339
78	ssc-let-7g	0.119987995	-0.473481784
79	ssc-miR-27a	0.982634191	0.007057051

Supp. Figure 36. Intra-modular analysis for MEM (Red module). The figure shows the scatter plot of GS (y-axis) vs. MM (x-axis) for phenotypic trait ashes in MEM red. The **GS** is the absolute value describing the relationship between the miRNA and the phenotypic trait ashes.

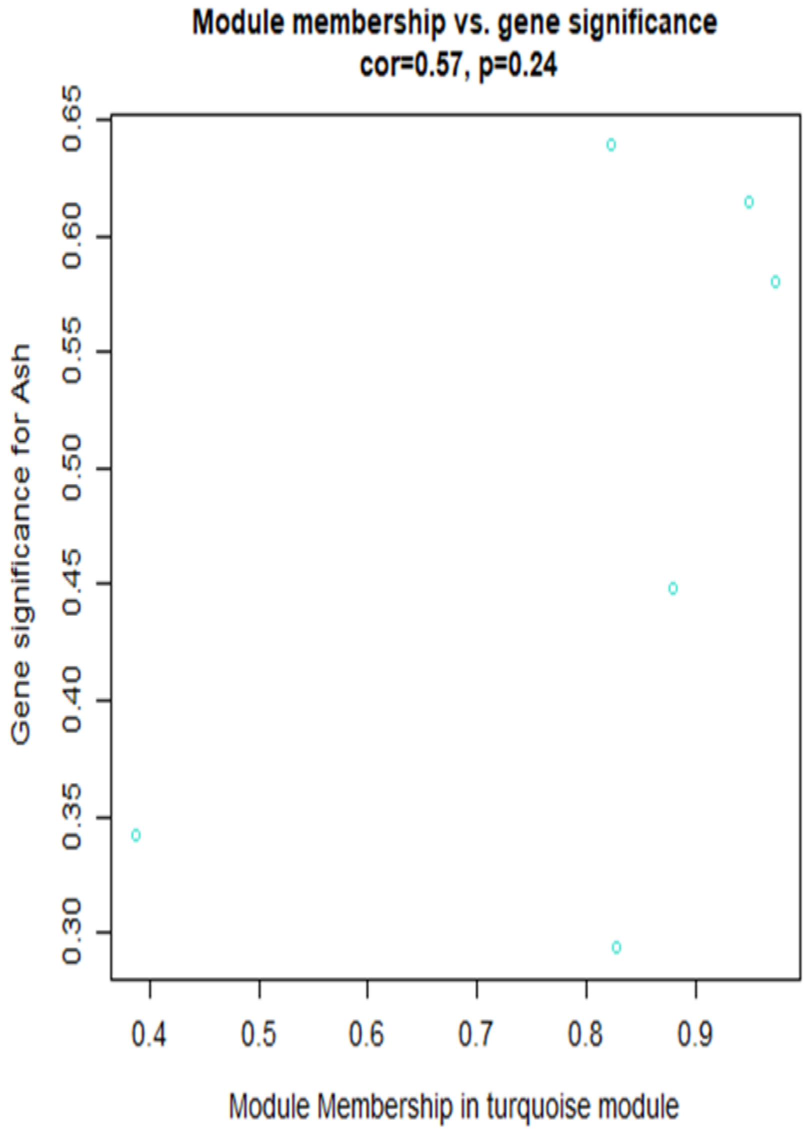

SN	Ensembl	p.GS	GS
1	ssc-miR-182	0.040230046	-0.597439952
2	ssc-miR-181c	0.10977179	-0.485278644
3	ssc-miR-130a	0.145743069	-0.446406062
4	ssc-miR-1285	0.163323676	-0.429656999
5	ssc-miR-92b-3p	0.212753505	-0.387924353
6	ssc-miR-146b	0.831635793	-0.068847932

Supp. Figure 37. Intra-modular analysis for MEM (Turquoise module). The figure shows the scatter plot of GS (y-axis) vs. MM (x-axis) for phenotypic trait ashes in MEM turquoise. The **GS** is the absolute value describing the relationship between the miRNA and the phenotypic trait ashes.

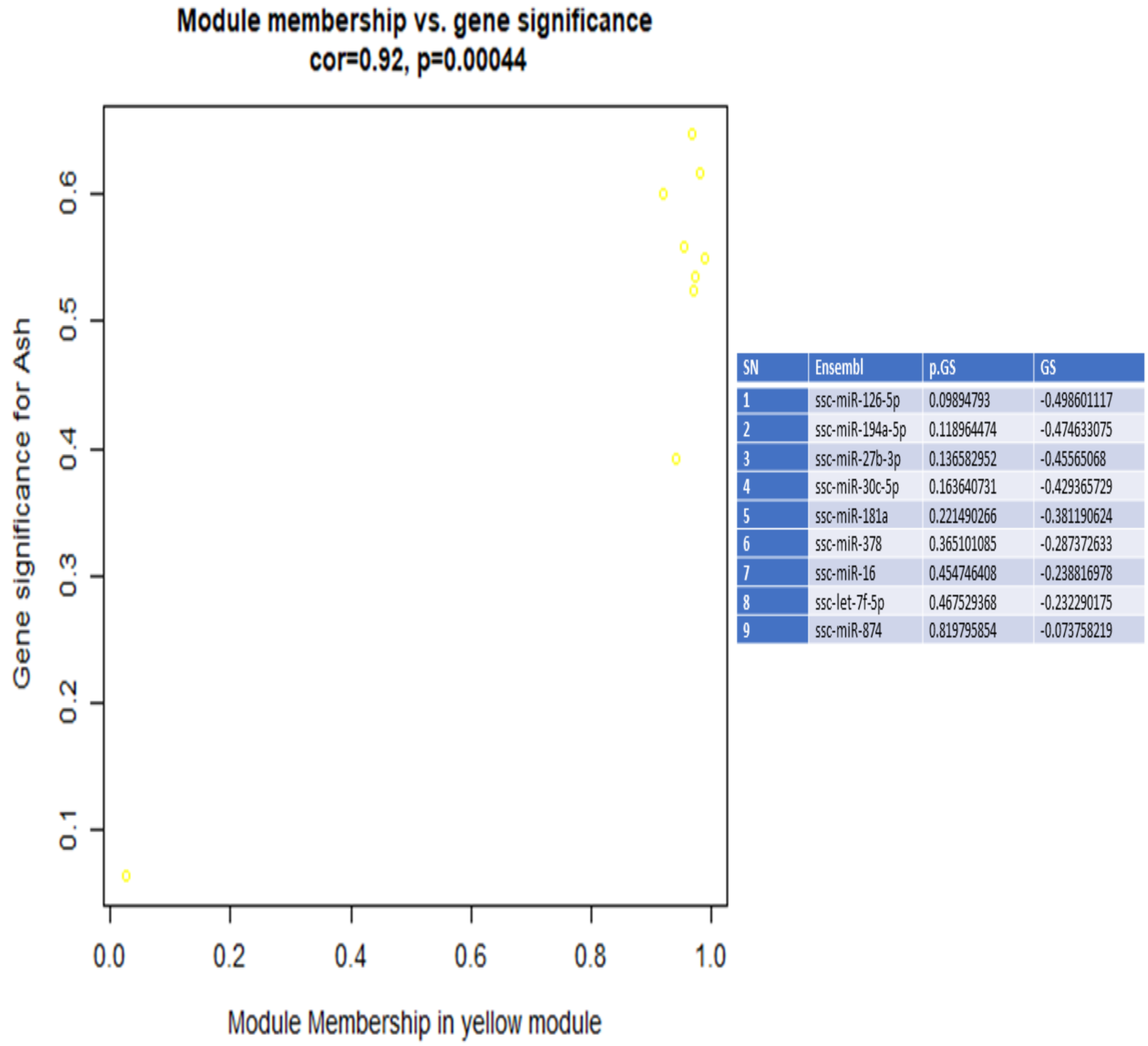

SN	Ensembl	p.GS	GS
1	ssc-miR-126-5p	0.09894793	-0.498601117
2	ssc-miR-194a-5p	0.118964474	-0.474633075
3	ssc-miR-27b-3p	0.136582952	-0.45565068
4	ssc-miR-30c-5p	0.163640731	-0.429365729
5	ssc-miR-181a	0.221490266	-0.381190624
6	ssc-miR-378	0.365101085	-0.287372633
7	ssc-miR-16	0.454746408	-0.238816978
8	ssc-let-7f-5p	0.467529368	-0.232290175
9	ssc-miR-874	0.819795854	-0.073758219

Supp. Figure 38. Intra-modular analysis for MEM (Yellow module). The figure shows the scatter plot of GS (y-axis) vs. MM (x-axis) for phenotypic trait ashes in MEM yellow. The **GS** is the absolute value describing the relationship between the miRNA and the phenotypic trait ashes.

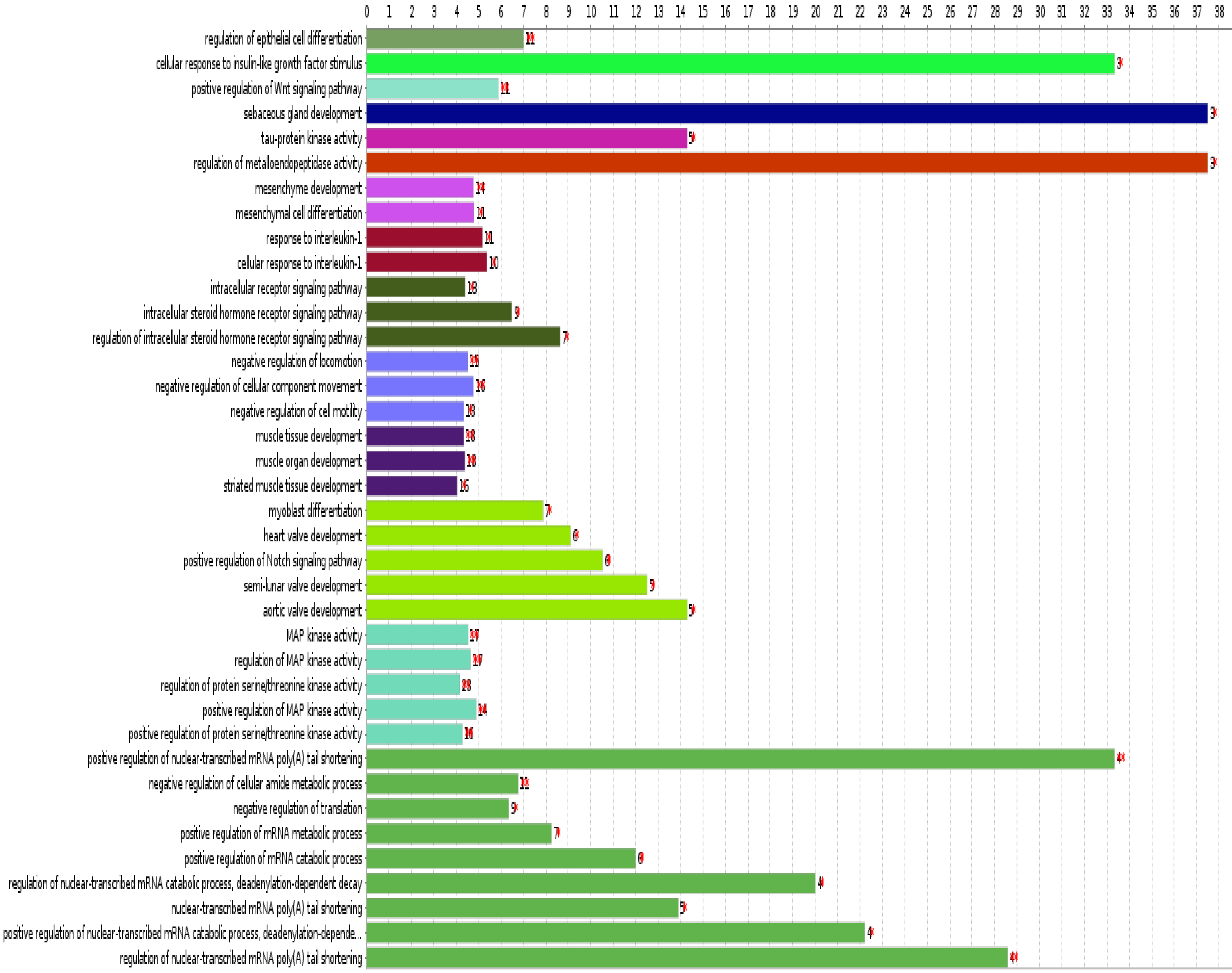

Supp. Figure 39. ClueGO analysis of black module miRNAs' target genes: The Figure shows the **GO/pathway terms** specific for black module miRNAs' target genes. The bars represent the number of genes associated with the terms. The percentage of genes per term is shown as bar label.

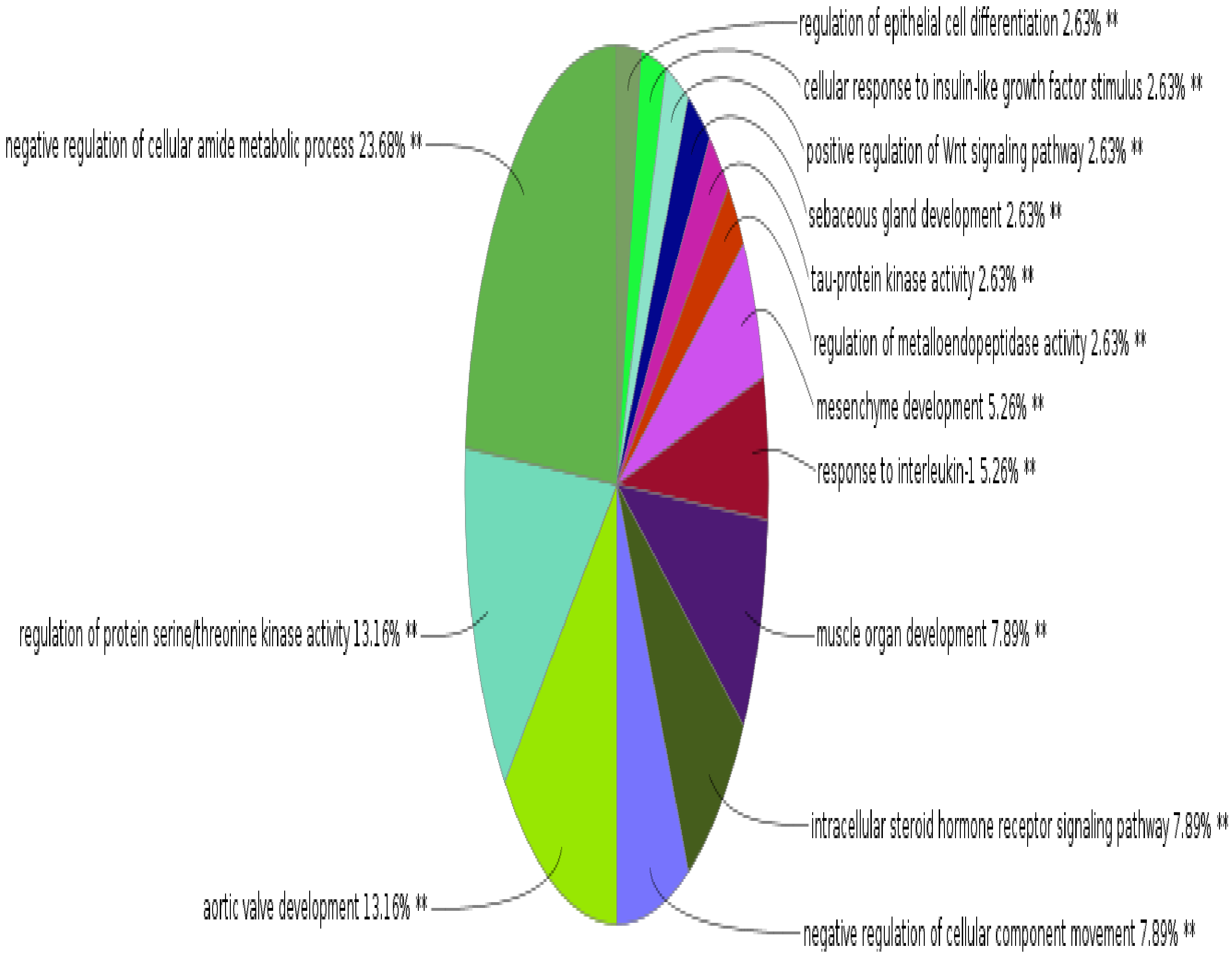

Supp. Figure 40. ClueGO analysis of black module miRNAs' target genes: The Figure shows an overview chart with **functional groups** including specific terms for black module miRNAs' target genes.

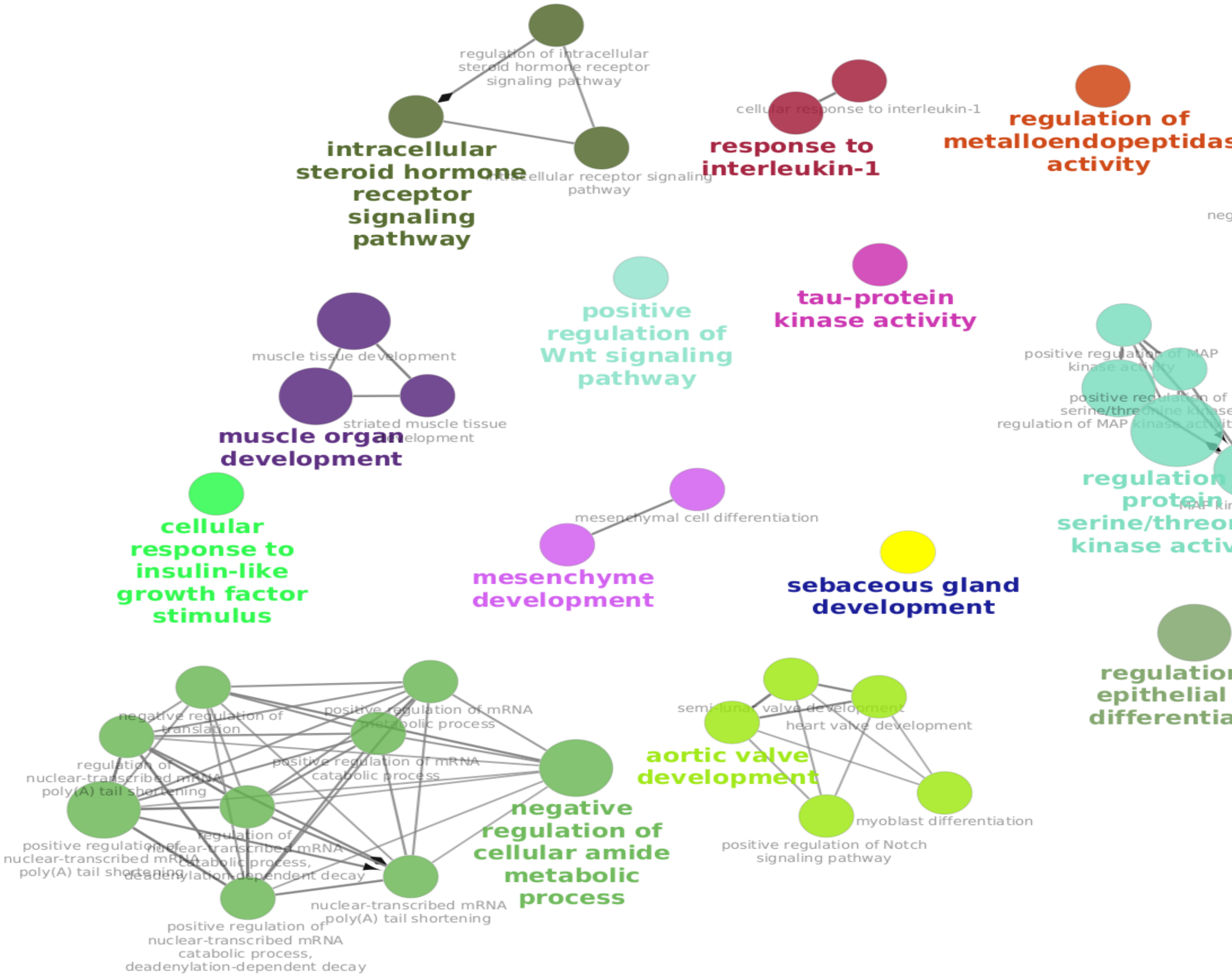

Supp. Figure 41. The distribution of all pathway terms (for black module miRNAs' target genes) visualized on the network. The Figure shows the functionally grouped network with terms as nodes (hubs) linked based on their kappa score level (≥0.3) and p-value after Bonferroni correction < 0.05, where only the label of the most significant term per group is shown. The node size represents the term enrichment significance. Node color represents the functional groups

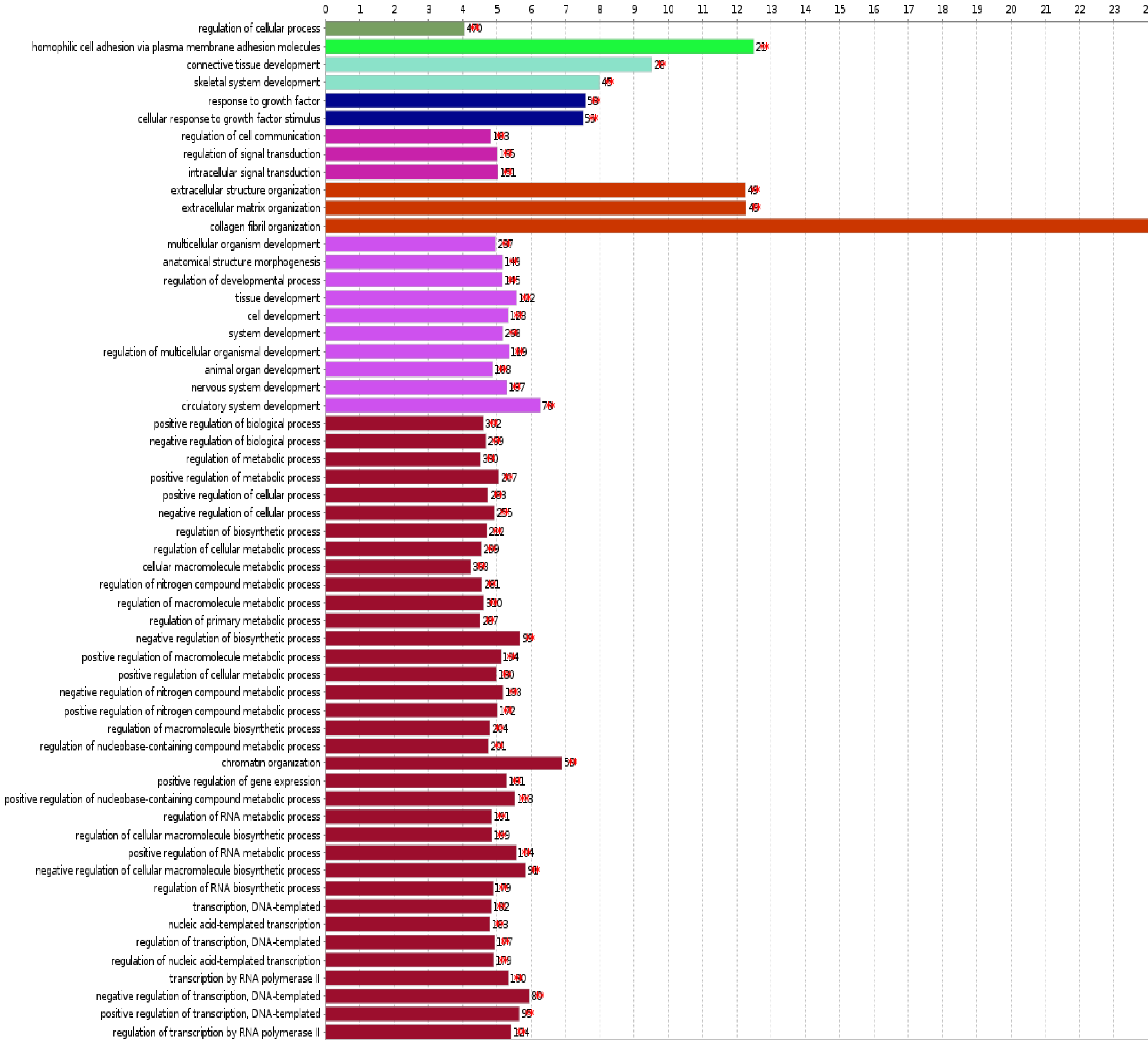

Supp. Figure 42. ClueGO analysis of blue module miRNAs' target genes: The Figure shows the GO/pathway terms specific for black module miRNAs' target genes. The bars represent the number of genes associated with the terms. The percentage of genes per term is shown as bar label.

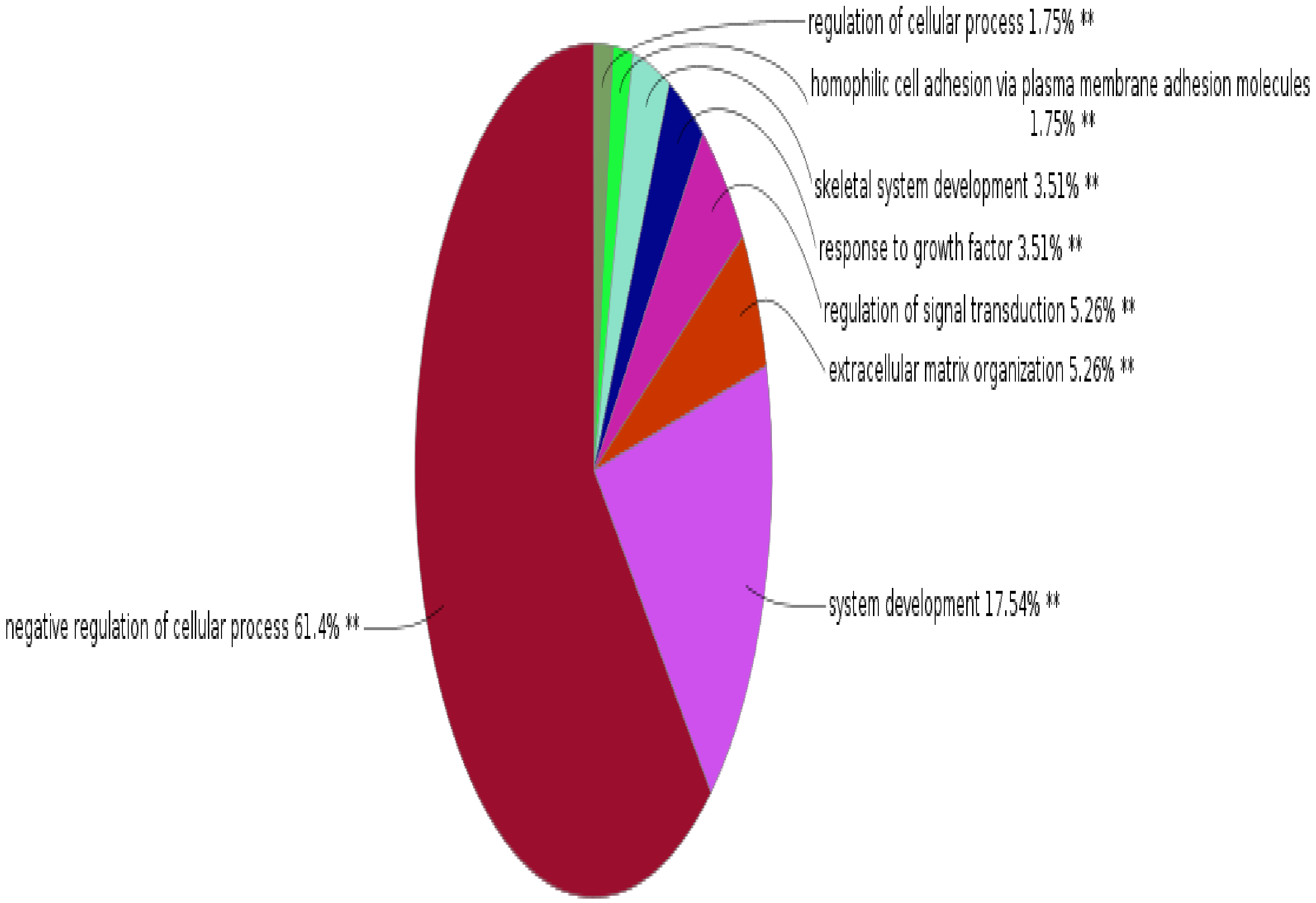

Supp. Figure 43. ClueGO analysis of blue module miRNAs' target genes: The Figure shows an overview chart with functional groups including specific terms for black module miRNAs' target genes.

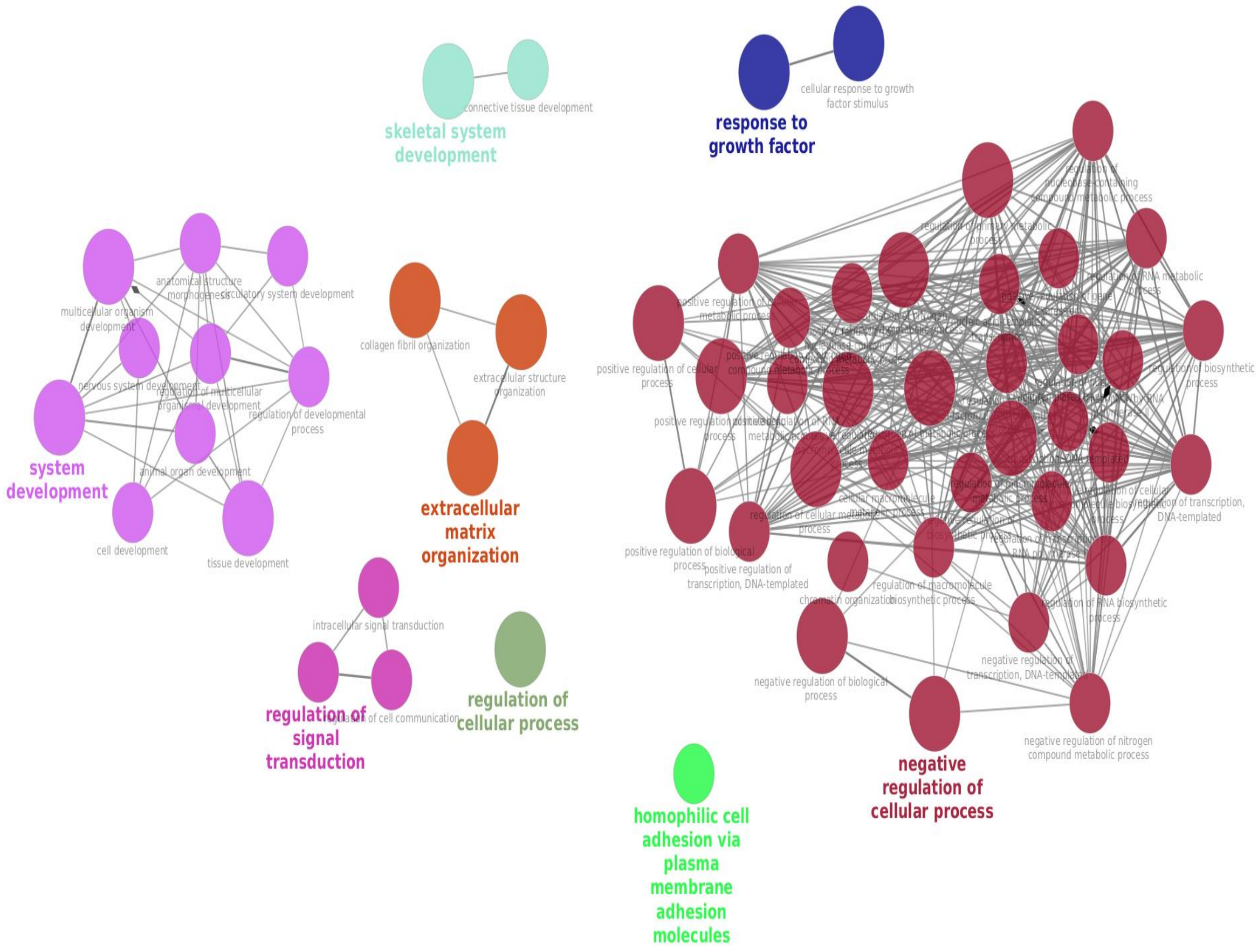

Supp. Figure 44. The distribution of all pathway terms (for blue module miRNAs' target genes) visualized on the network. The Figure shows the functionally grouped network with terms as nodes (hubs) linked based on their kappa score level (≥0.3) and p-value after Bonferroni correction < 0.05, where only the label of the most significant term per group is shown. The node size represents the term enrichment significance. Node color represents the functional groups.

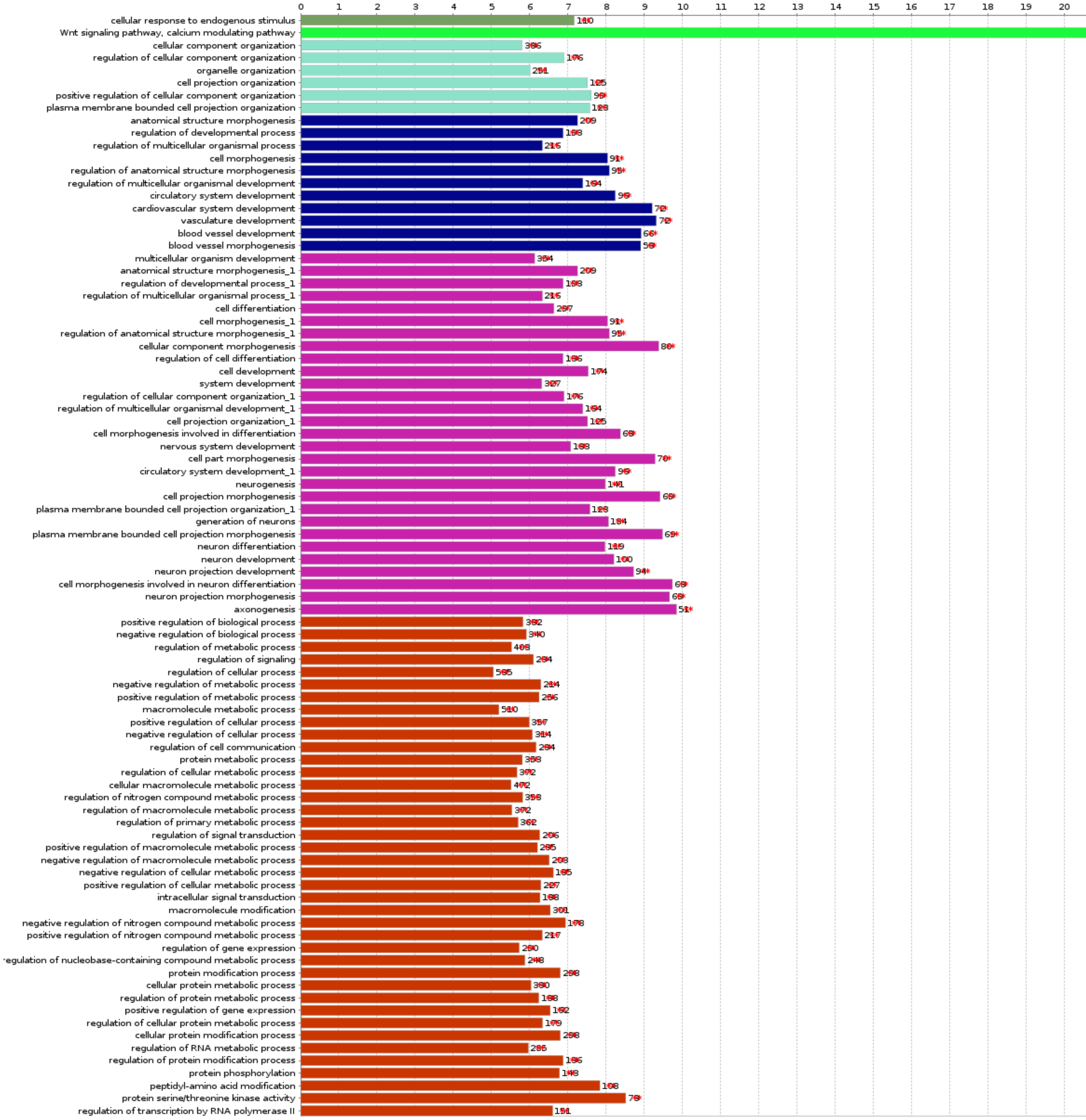

Supp. Figure 45. ClueGO analysis of brown module miRNAs' target genes: The Figure shows the GO/pathway terms specific for black module miRNAs' target genes. The bars represent the number of genes associated with the terms. The percentage of genes per term is shown as bar label.

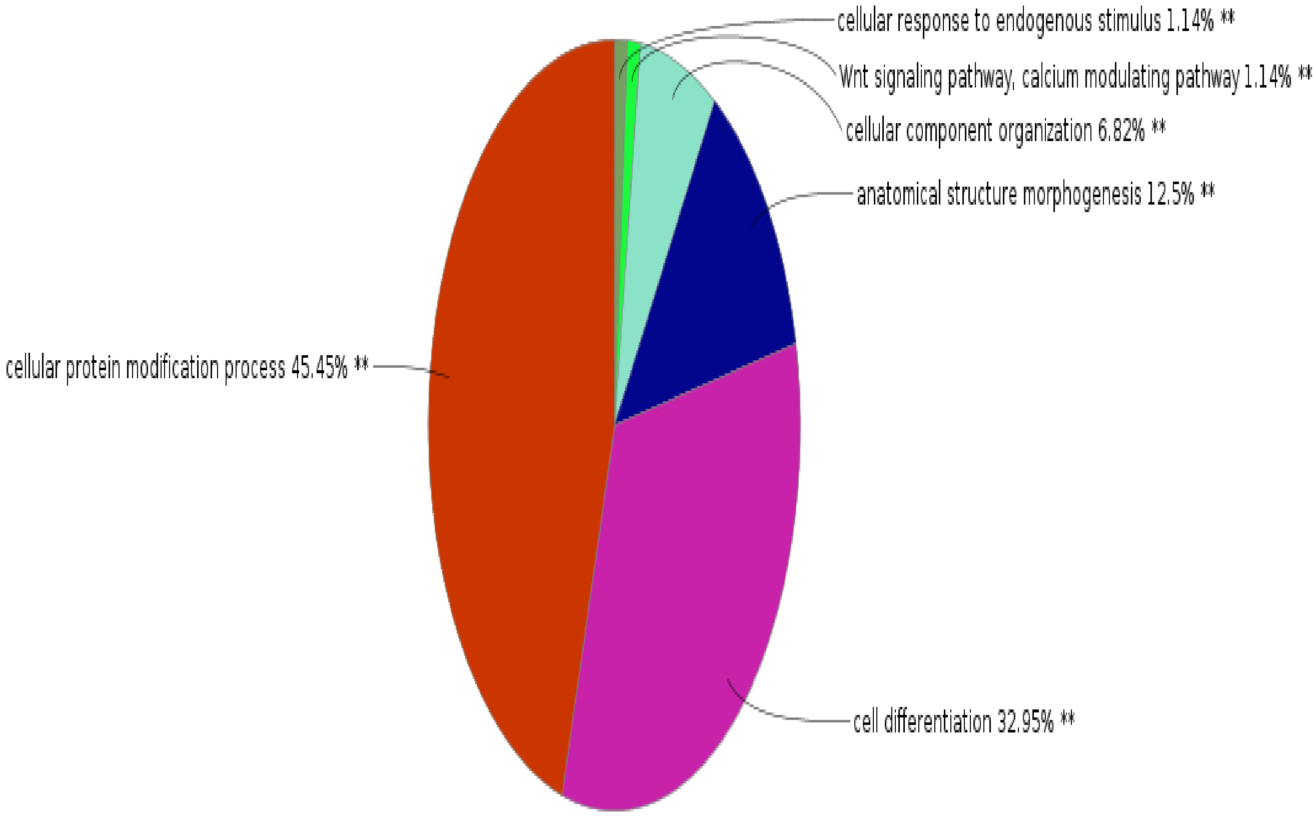

Supp. Figure 46. ClueGO analysis of brown module miRNAs' target genes: The Figure shows an overview chart with functional groups including specific terms for black module miRNAs' target genes.

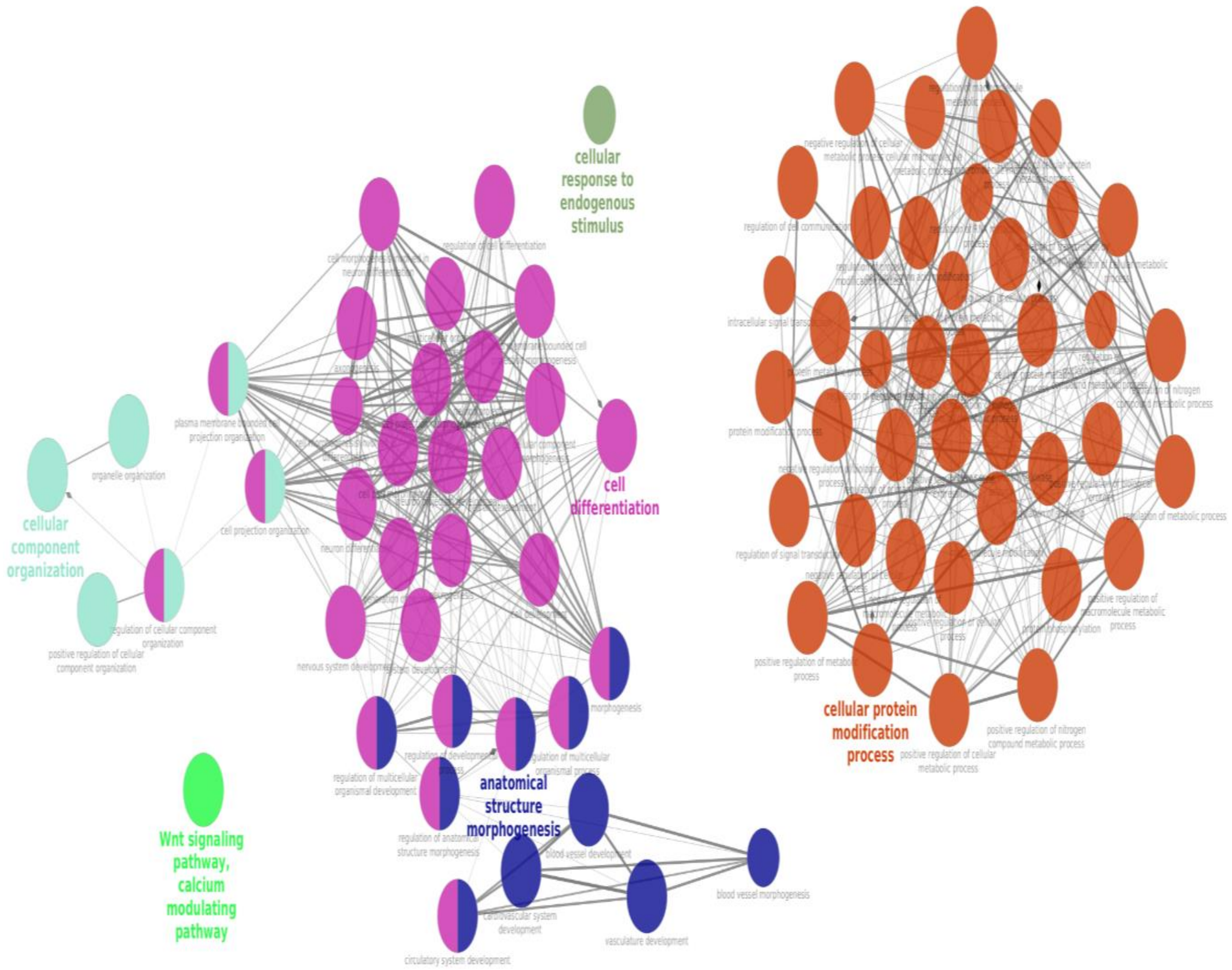

Supp. Figure 47. The distribution of all pathway terms (for brown module miRNAs' target genes) visualized on the network. The Figure shows the functionally grouped network with terms as nodes (hubs) linked based on their kappa score level (≥0.3) and p-value after Bonferroni correction < 0.05, where only the label of the most significant term per group is shown. The node size represents the term enrichment significance. Node color represents the functional groups.

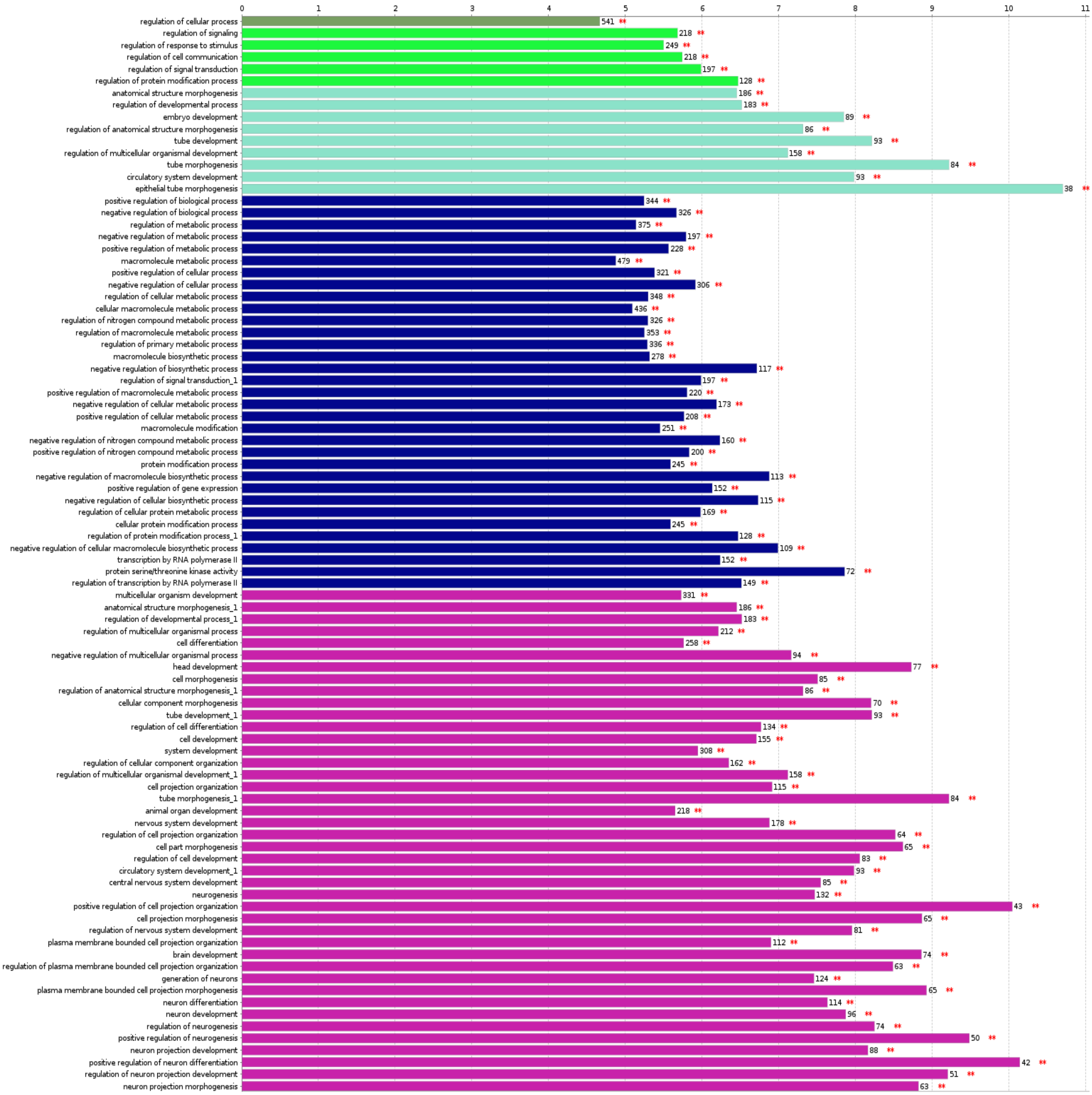

Supp. Figure 48. ClueGO analysis of green module miRNAs' target genes: The Figure shows the GO/pathway terms specific for black module miRNAs' target genes. The bars represent the number of genes associated with the terms. The percentage of genes per term is shown as bar label.

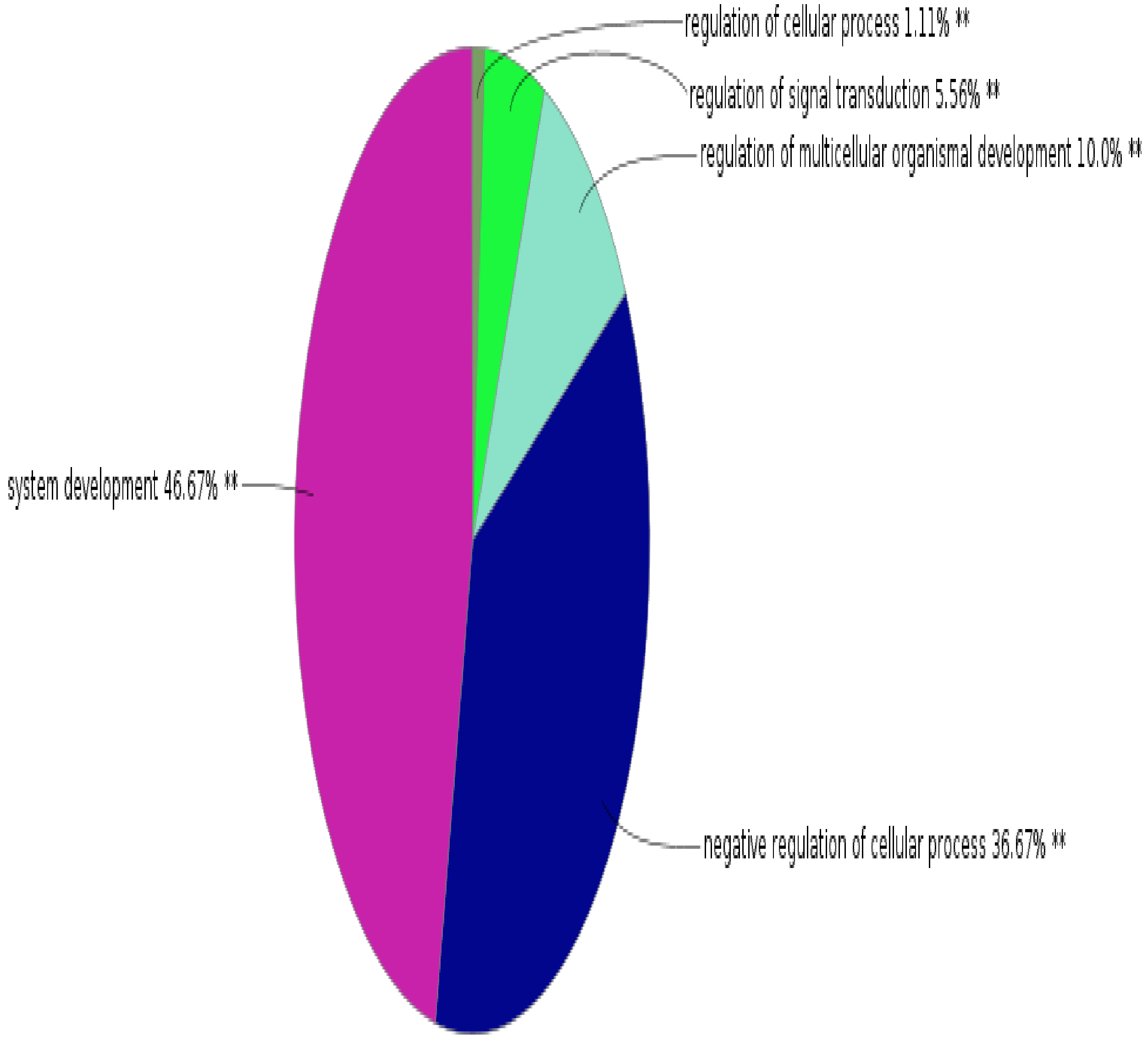

Supp. Figure 49. ClueGO analysis of green module miRNAs' target genes: The Figure shows an overview chart with functional groups including specific terms for black module miRNAs' target genes.

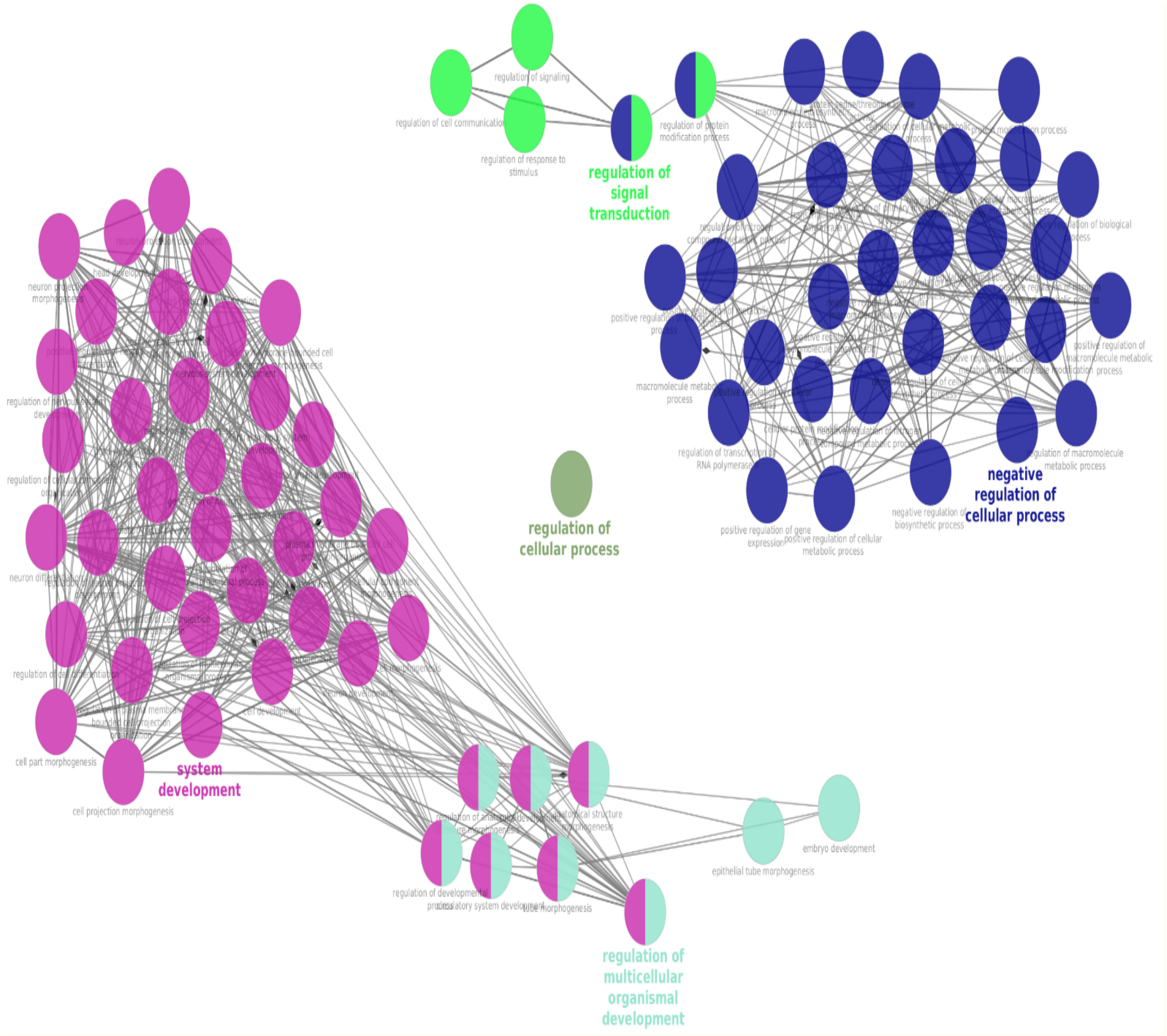

Supp. Figure 50. The distribution of all pathway terms (for green module miRNAs' target genes) visualized on the network. The Figure shows the functionally grouped network with terms as nodes (hubs) linked based on their kappa score level (≥0.3) and p-value after Bonferroni correction < 0.05, where only the label of the most significant term per group is shown. The node size represents the term enrichment significance. Node color represents the functional groups.

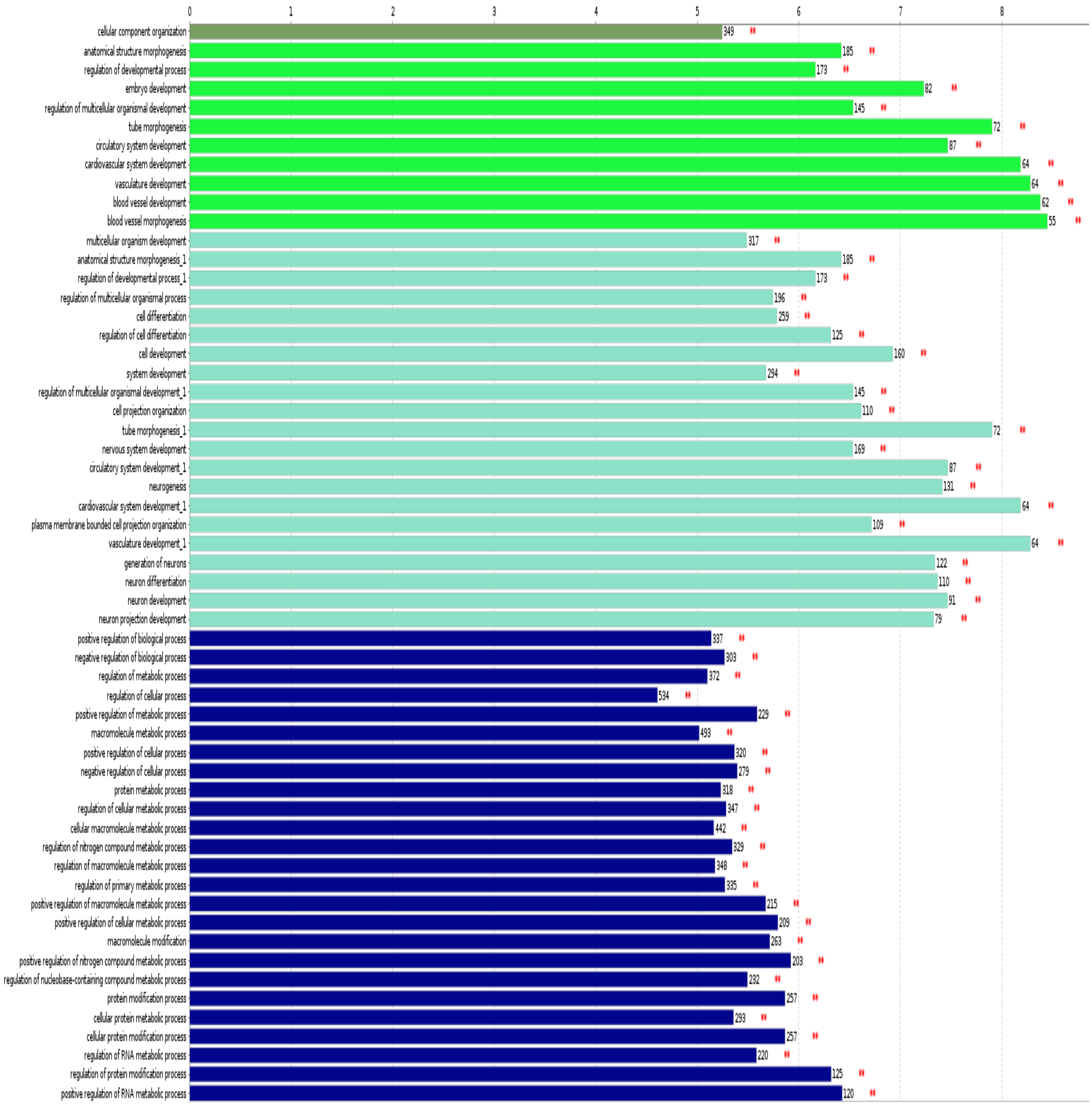

Supp. Figure 51. ClueGO analysis of magenta module miRNAs' target genes: The Figure shows the GO/pathway terms specific for black module miRNAs' target genes. The bars represent the number of genes associated with the terms. The percentage of genes per term is shown as bar label.

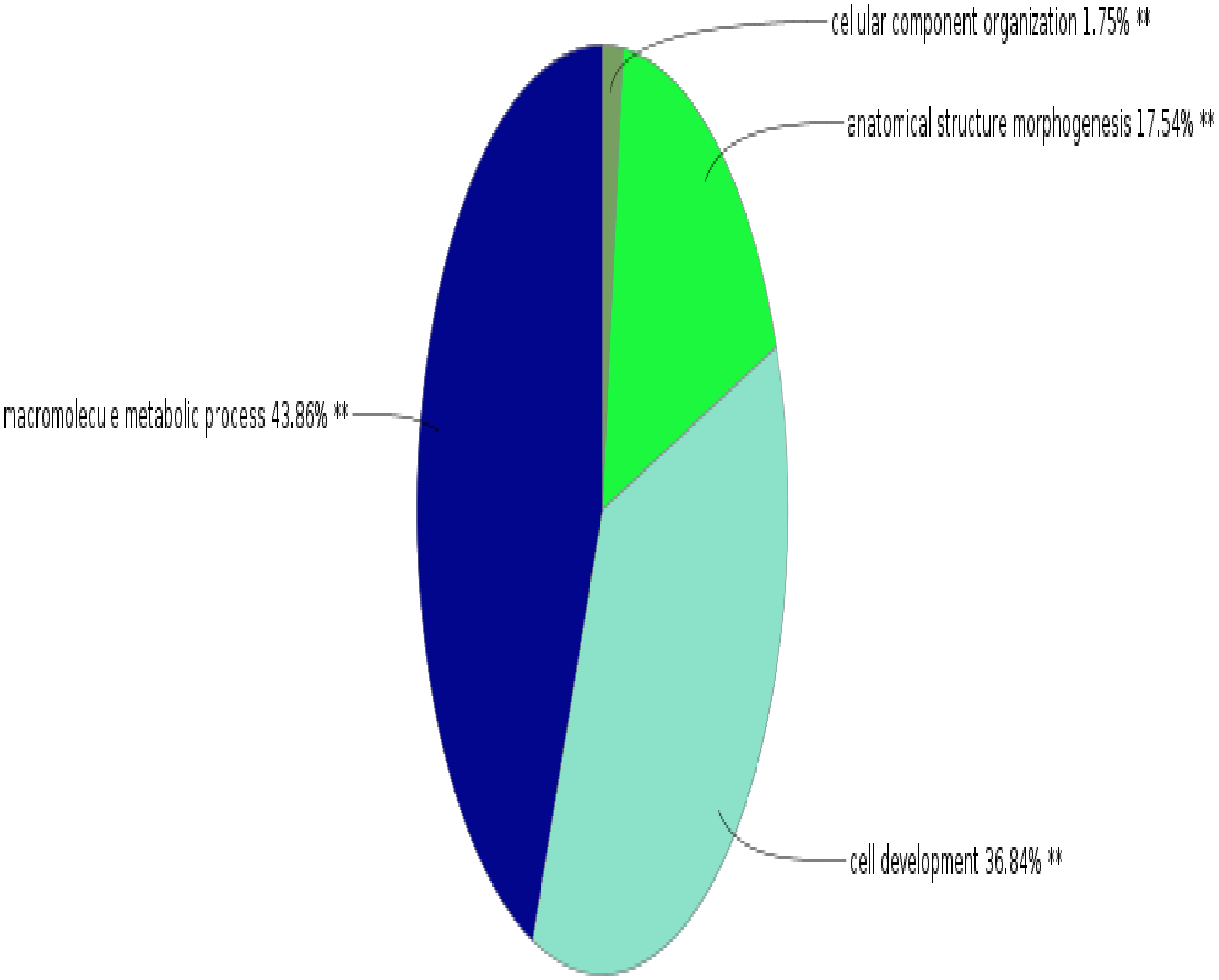

Supp. Figure 52. ClueGO analysis of magenta module miRNAs' target genes: The Figure shows an overview chart with functional groups including specific terms for black module miRNAs' target genes.

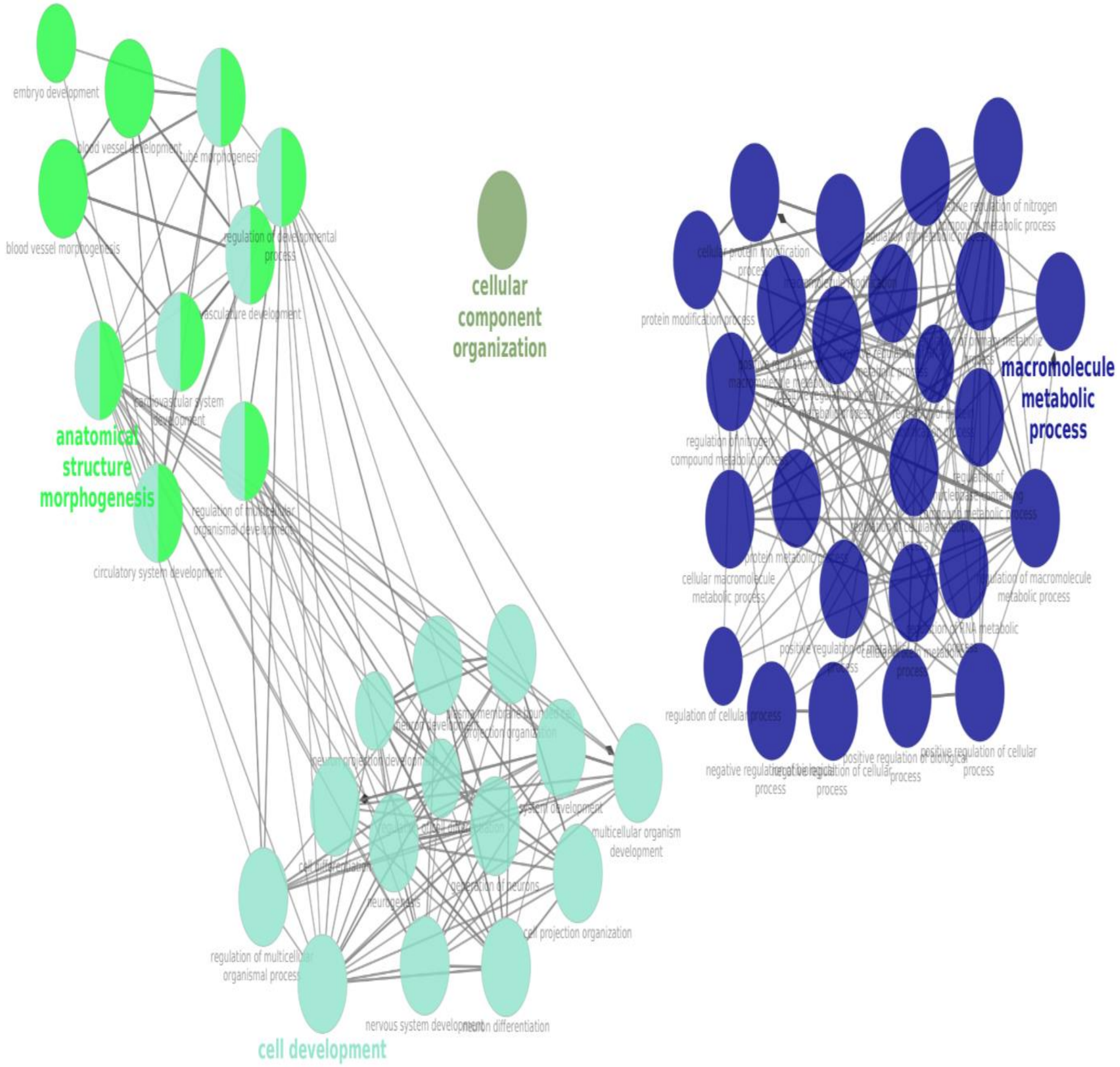

Supp. Figure 53. The distribution of all pathway terms (for magenta module miRNAs' target genes) visualized on the network. The Figure shows the functionally grouped network with terms as nodes (hubs) linked based on their kappa score level (≥0.3) and p-value after Bonferroni correction < 0.05, where only the label of the most significant term per group is shown. The node size represents the term enrichment significance. Node color represents the functional groups

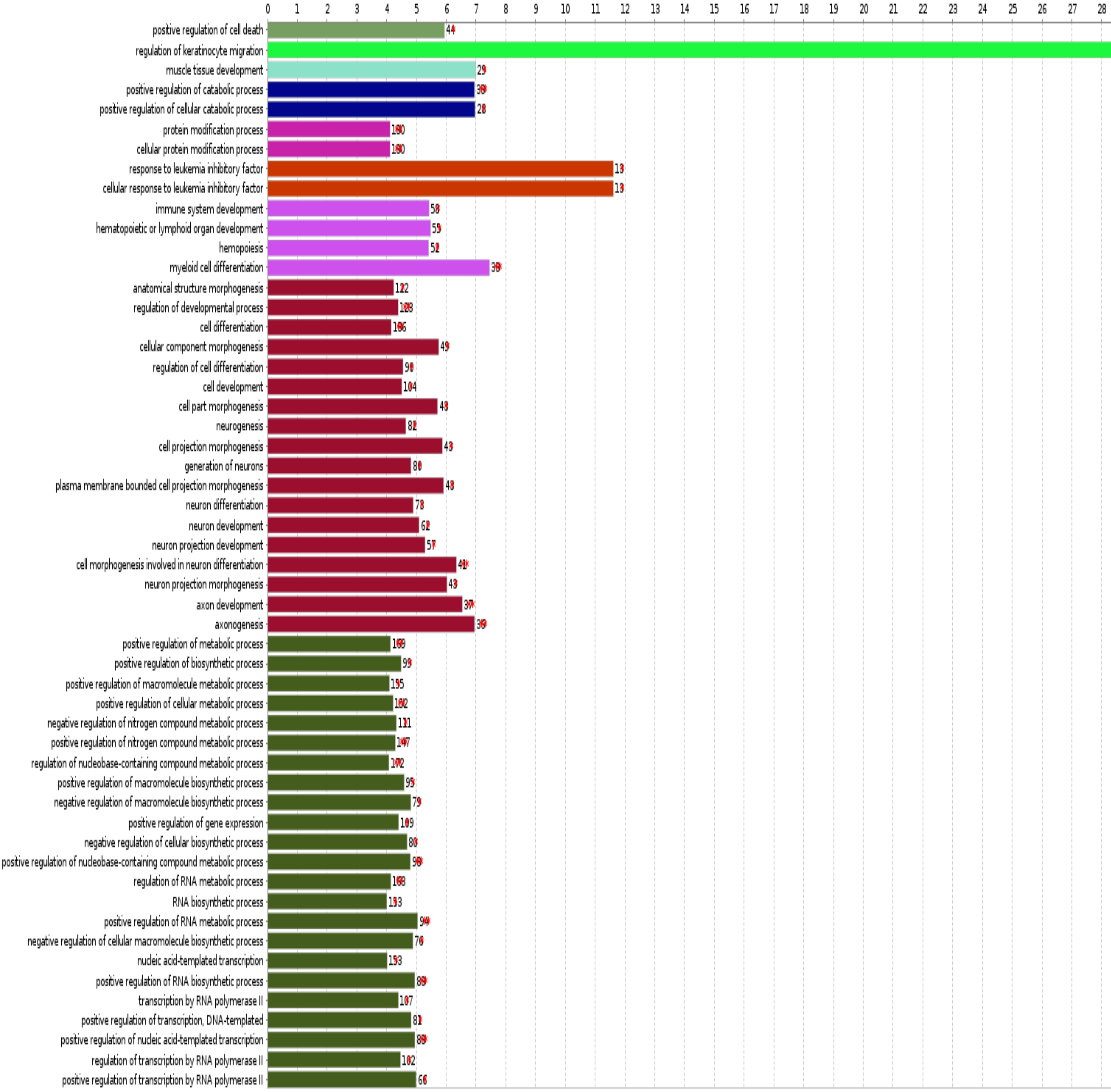

Supp. Figure 54. ClueGO analysis of pink module miRNAs' target genes: The Figure shows the GO/pathway terms specific for black module miRNAs' target genes. The bars represent the number of genes associated with the terms. The percentage of genes per term is shown as bar label.

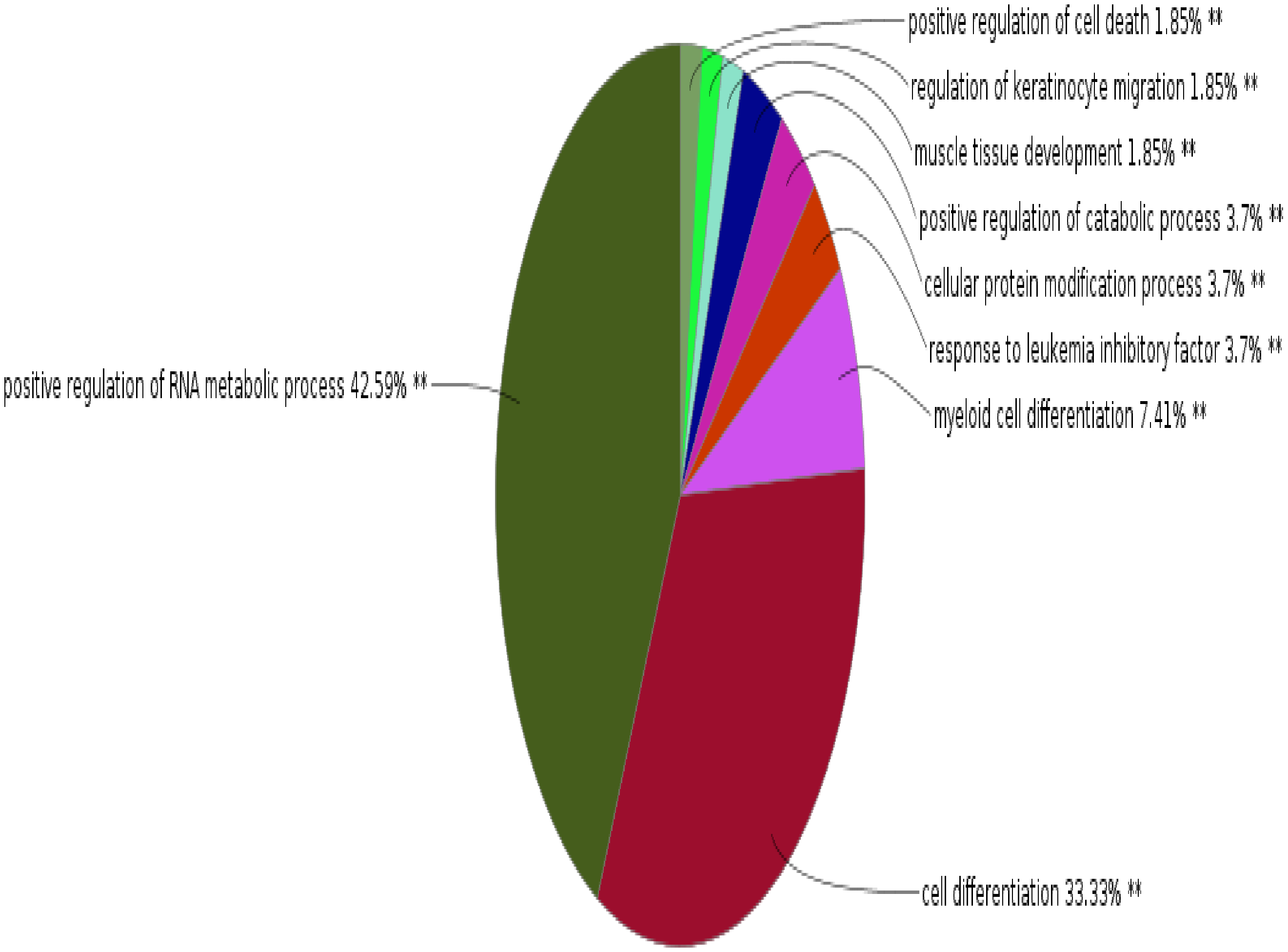

Supp. Figure 55. ClueGO analysis of pink module miRNAs' target genes: The Figure shows an overview chart with functional groups including specific terms for black module miRNAs' target genes.

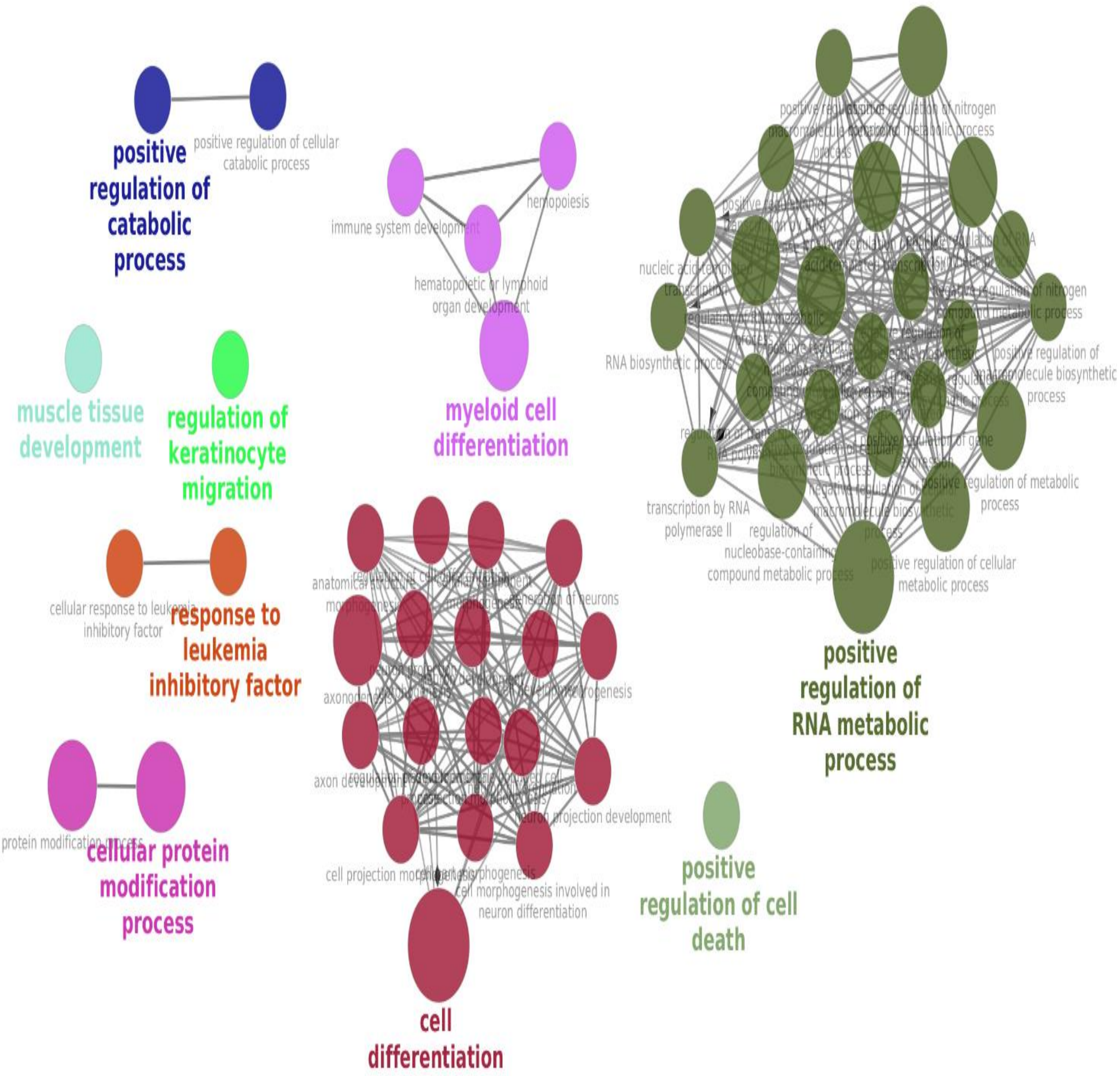

Supp. Figure 56. The distribution of all pathway terms (for pink module miRNAs' target genes) visualized on the network. The Figure shows the functionally grouped network with terms as nodes (hubs) linked based on their kappa score level (≥0.3) and p-value after Bonferroni correction < 0.05, where only the label of the most significant term per group is shown. The node size represents the term enrichment significance. Node color represents the functional groups.

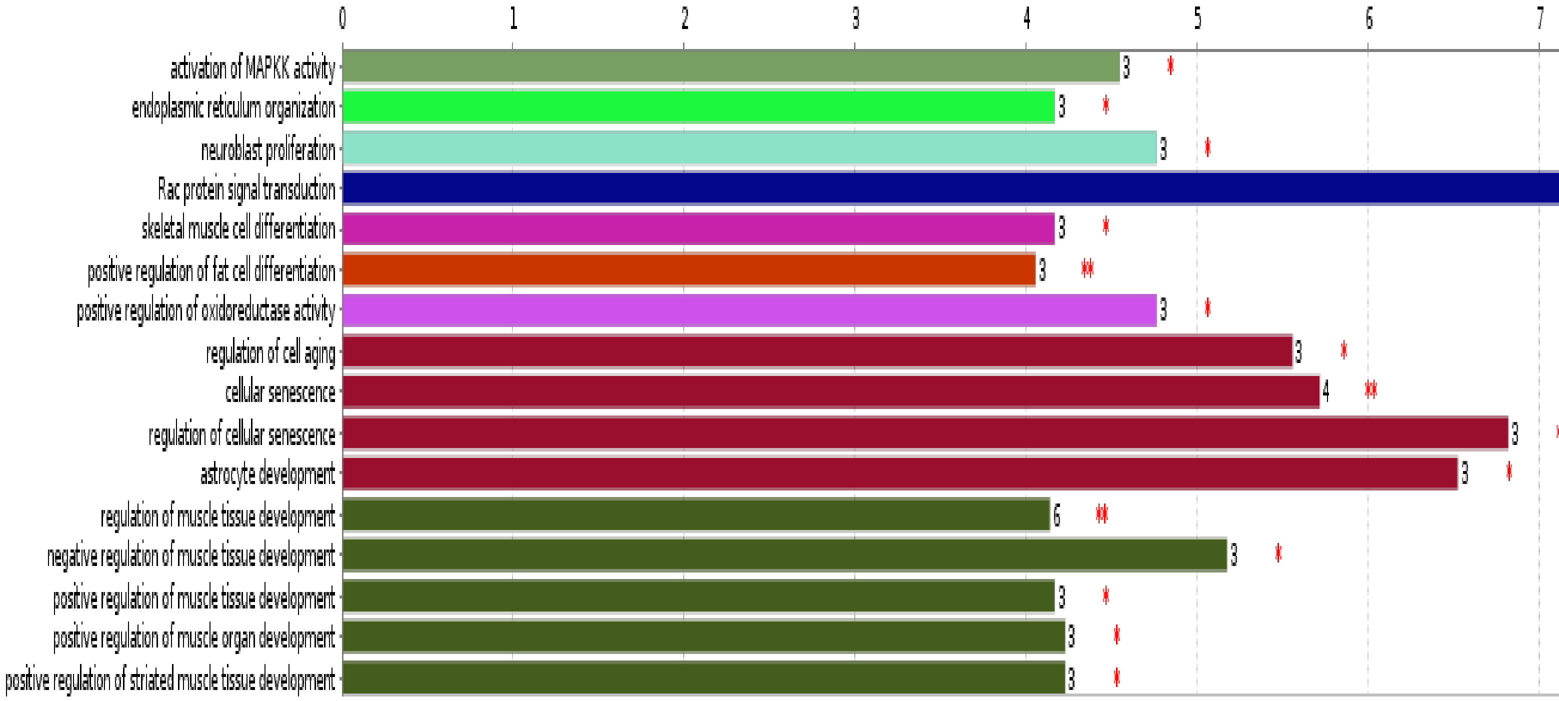

Supp. Figure 57. ClueGO analysis of purple module miRNAs' target genes: The Figure shows the GO/pathway terms specific for black module miRNAs' target genes. The bars represent the number of genes associated with the terms. The percentage of genes per term is shown as bar label.

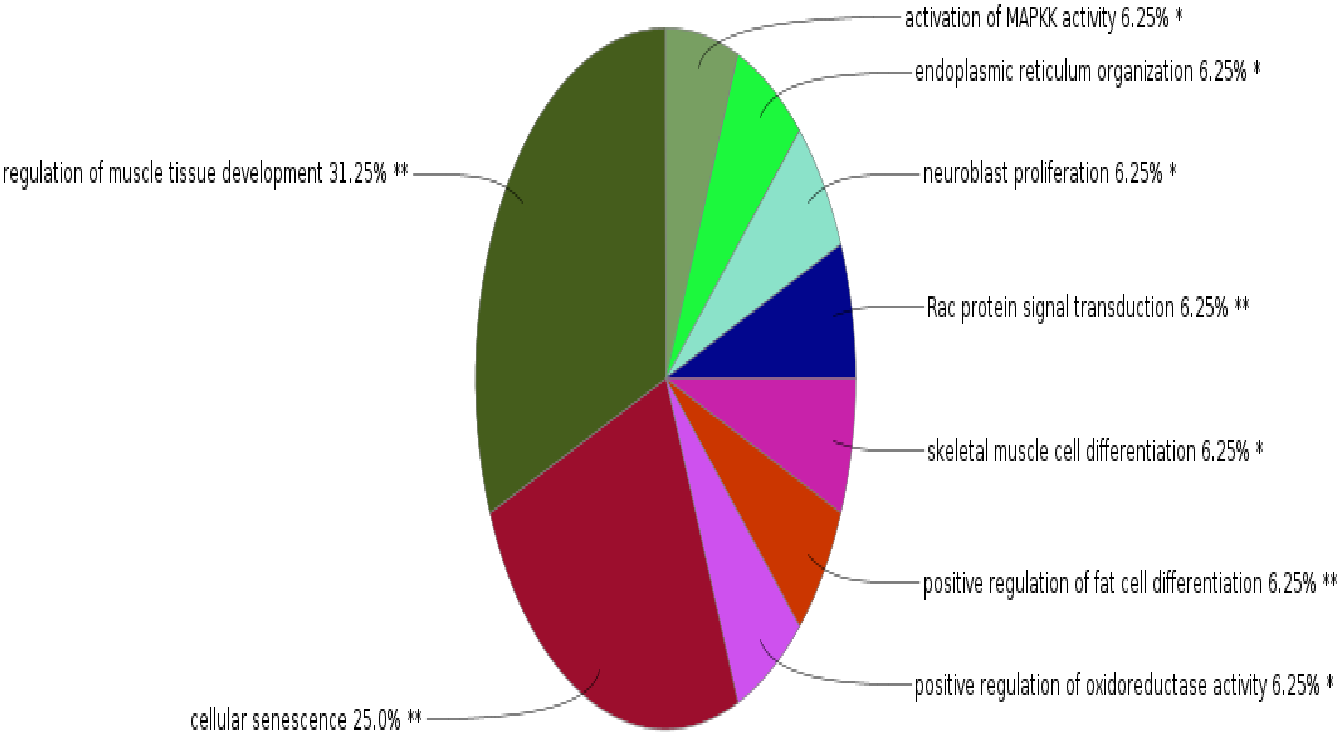

Supp. Figure 58. ClueGO analysis of purple module miRNAs' target genes: The Figure shows an overview chart with functional groups including specific terms for black module miRNAs' target genes.

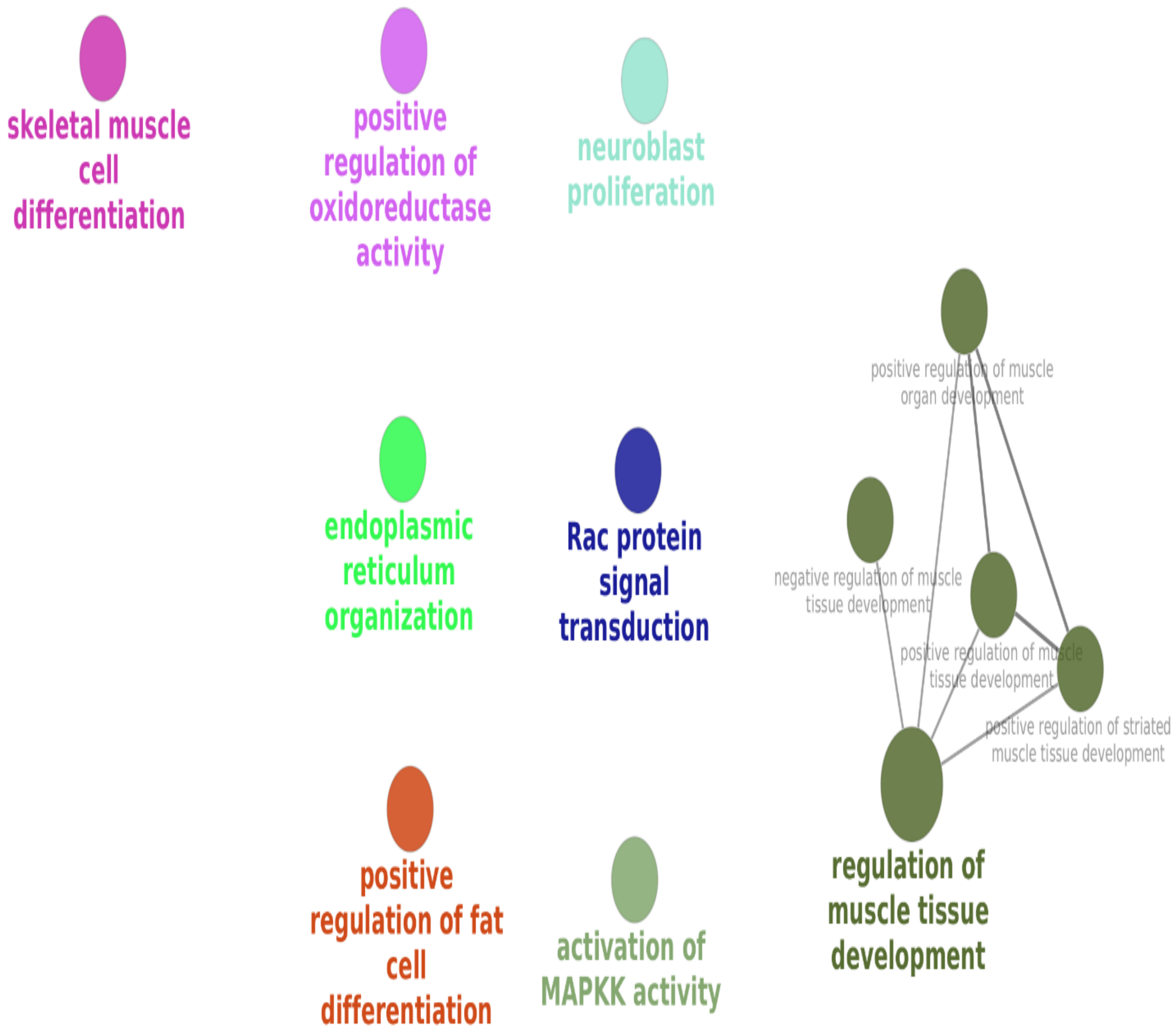

Supp. Figure 59. The distribution of all pathway terms (for purple module miRNAs' target genes) visualized on the network. The Figure shows the functionally grouped network with terms as nodes (hubs) linked based on their kappa score level (≥0.3) and p-value after Bonferroni correction < 0.05, where only the label of the most significant term per group is shown. The node size represents the term enrichment significance. Node color represents the functional groups.

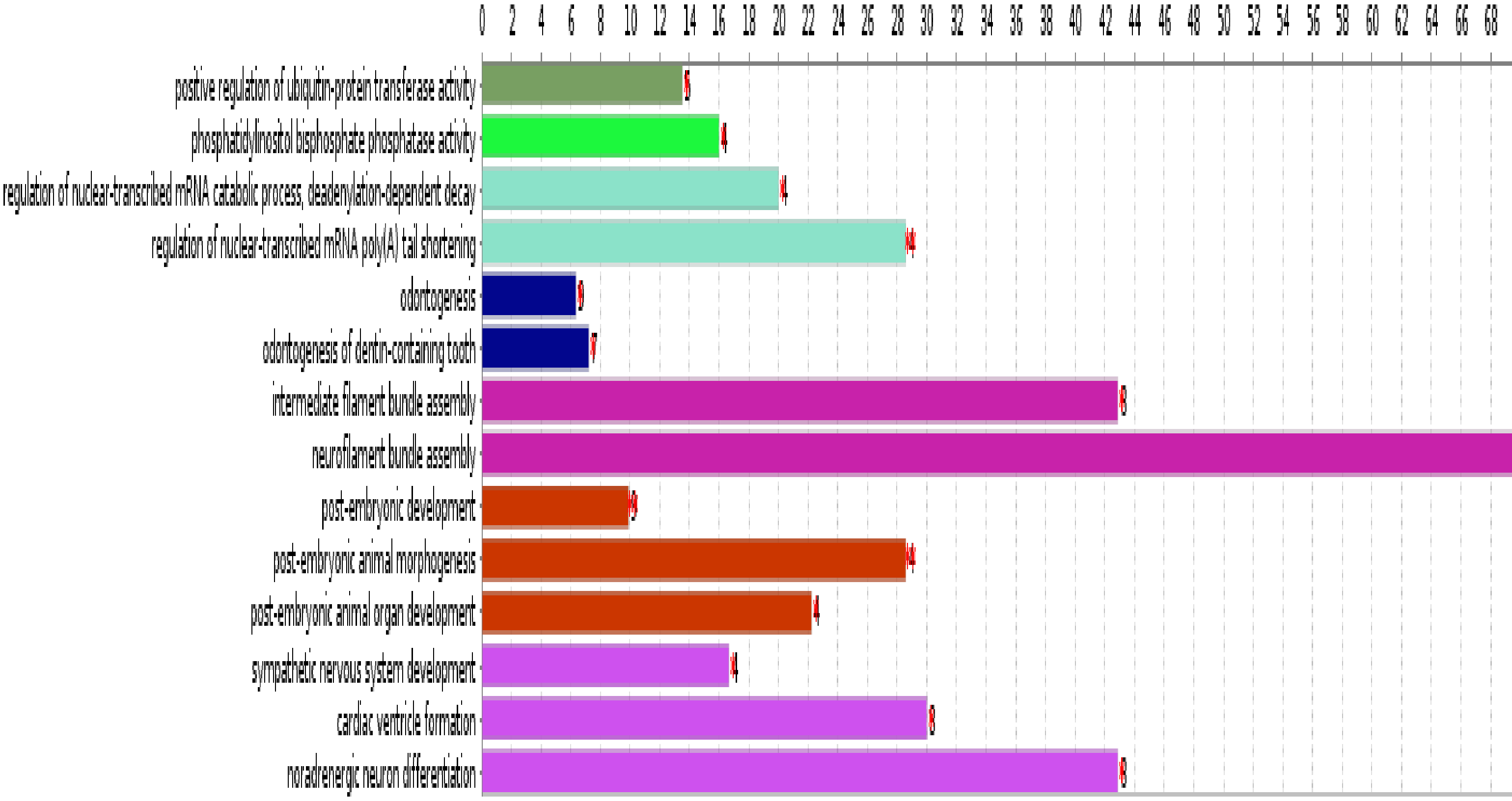

Supp. Figure 60. ClueGO analysis of turquoise module miRNAs' target genes: The Figure shows the GO/pathway terms specific for black module miRNAs' target genes. The bars represent the number of genes associated with the terms. The percentage of genes per term is shown as bar label.

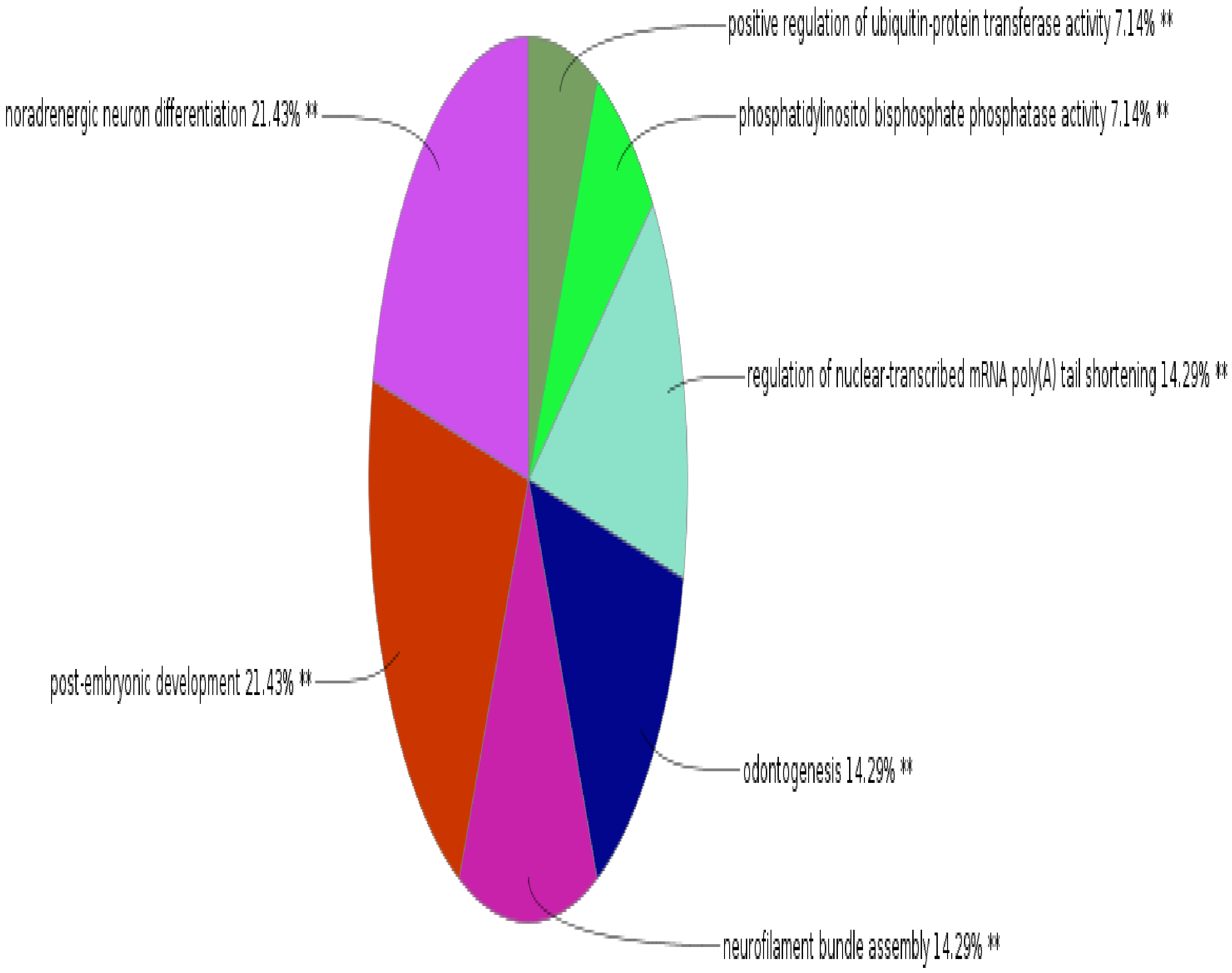

Supp. Figure 61. ClueGO analysis of turquoise module miRNAs' target genes: The Figure shows an overview chart with functional groups including specific terms for black module miRNAs' target genes.

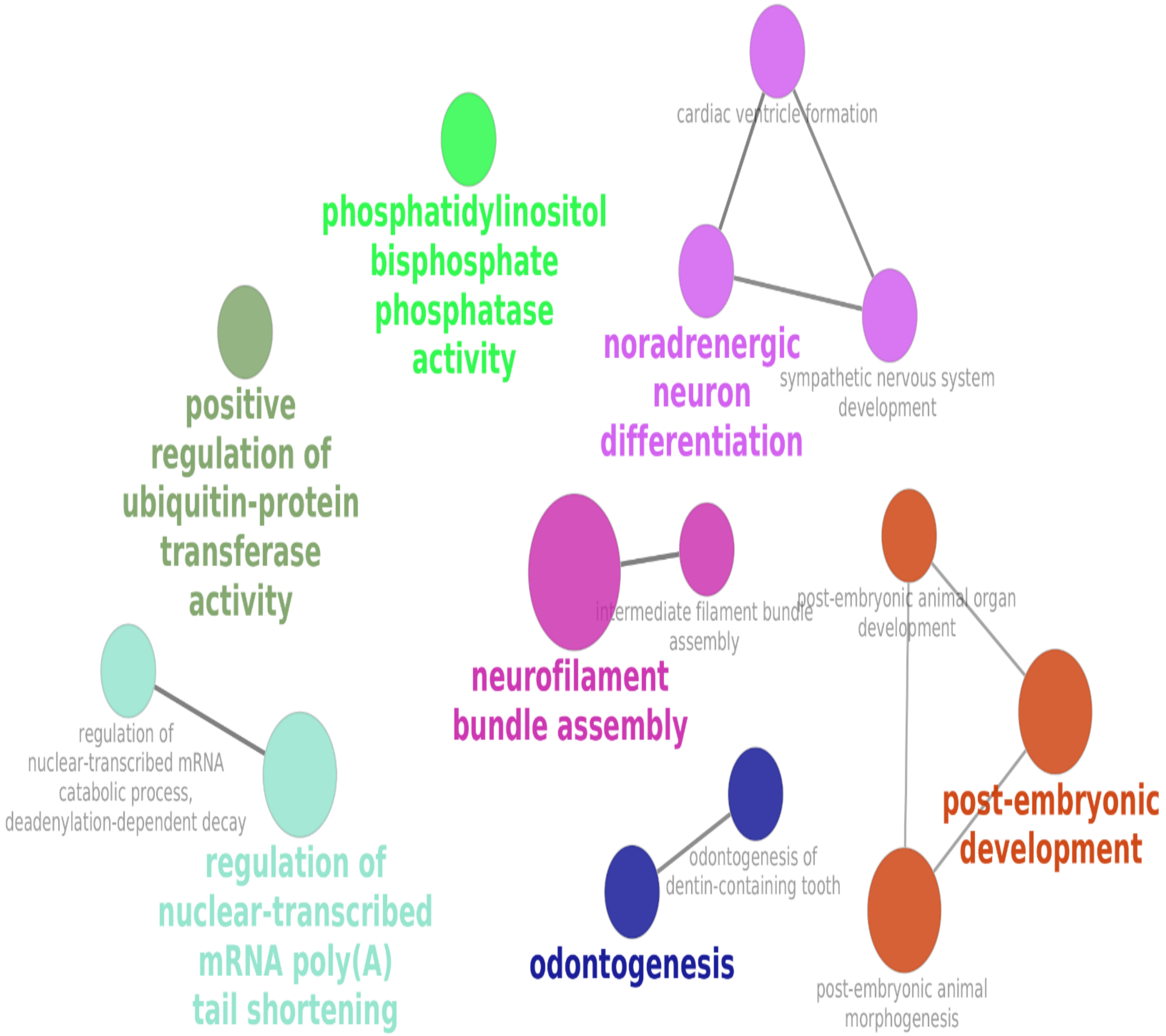

Supp. Figure 62. The distribution of all pathway terms (for turquoise module miRNAs' target genes) visualized on the network. The Figure shows the functionally grouped network with terms as nodes (hubs) linked based on their kappa score level (≥0.3) and p-value after Bonferroni correction < 0.05, where only the label of the most significant term per group is shown. The node size represents the term enrichment significance. Node color represents the functional groups

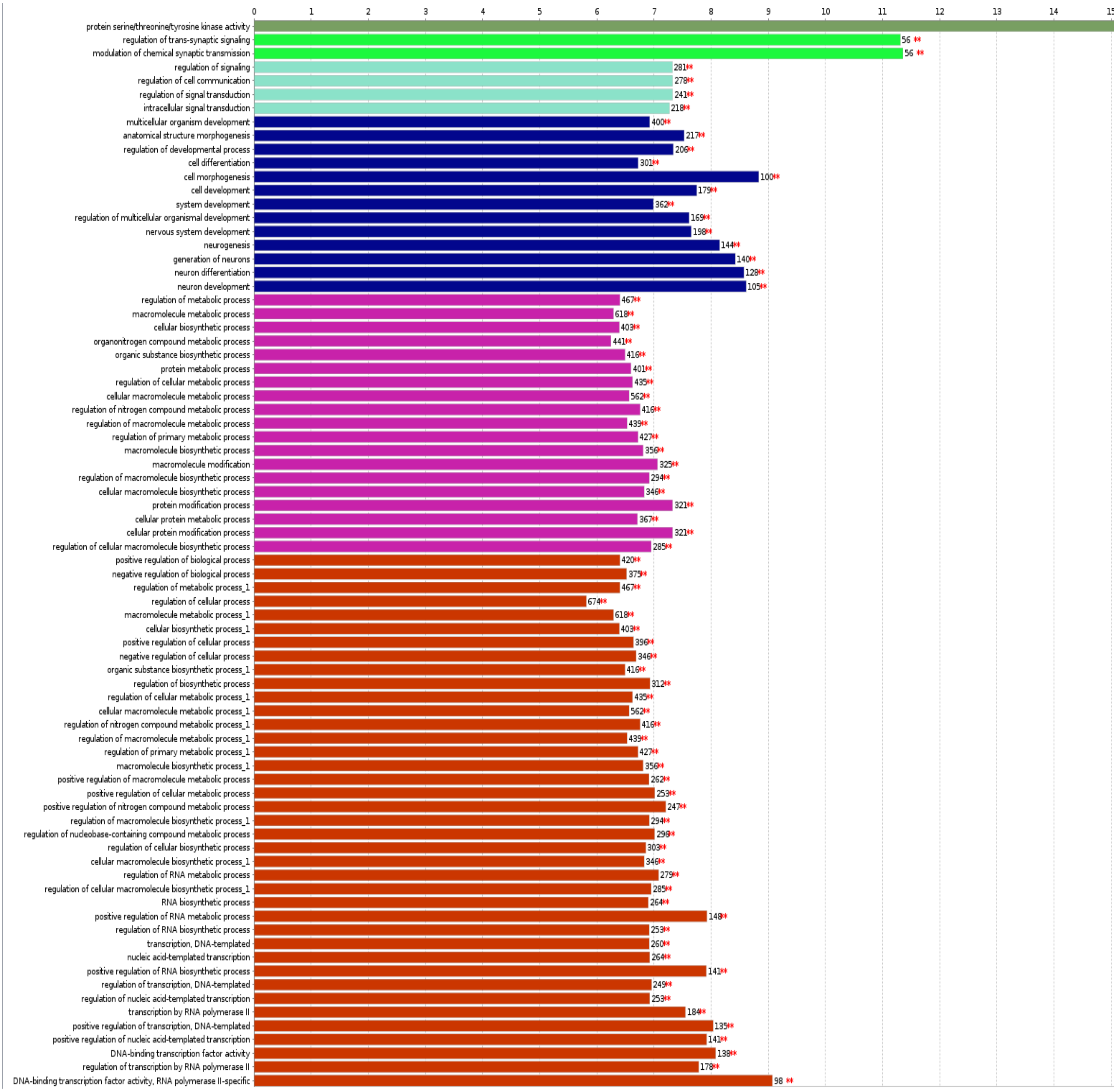

Supp. Figure 63. ClueGO analysis of yellow module miRNAs' target genes: The Figure shows the GO/pathway terms specific for black module miRNAs' target genes. The bars represent the number of genes associated with the terms. The percentage of genes per term is shown as bar label.

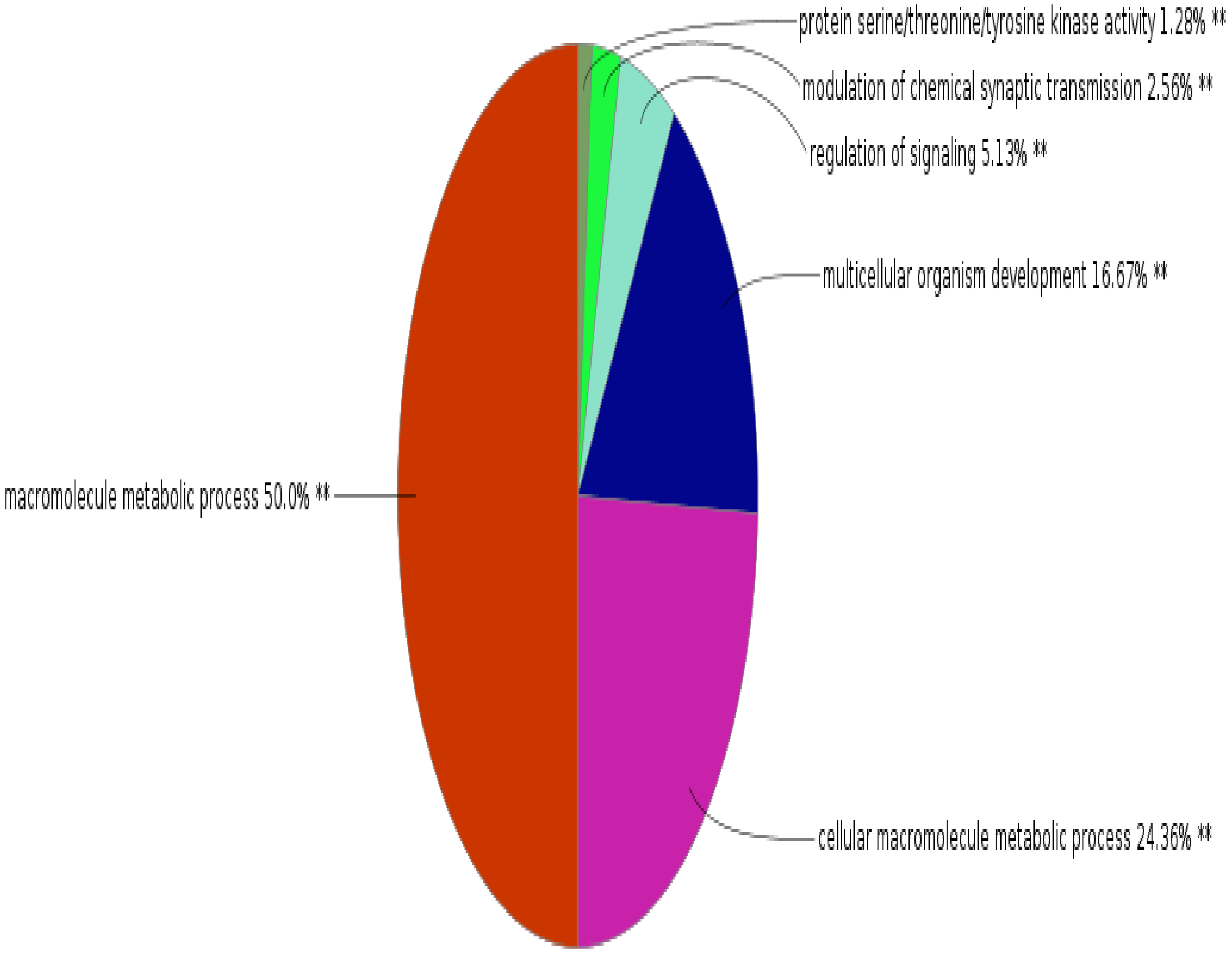

Supp. Figure 64. ClueGO analysis of yellow module miRNAs' target genes: The Figure shows an overview chart with functional groups including specific terms for black module miRNAs' target genes.

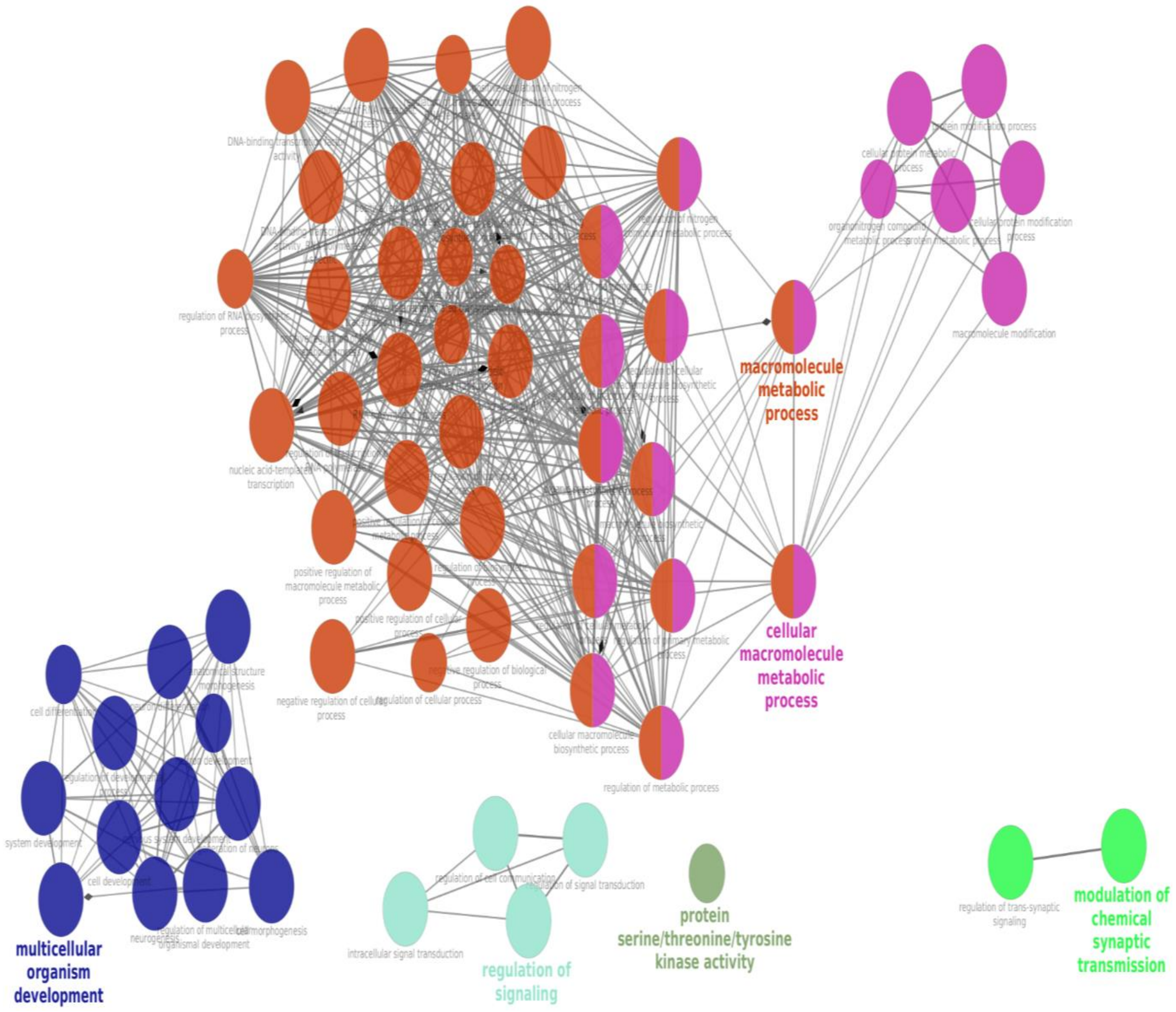

Supp. Figure 65. The distribution of all pathway terms (for yellow module miRNAs' target genes) visualized on the network. The Figure shows the functionally grouped network with terms as nodes (hubs) linked based on their kappa score level (≥0.3) and p-value after Bonferroni correction < 0.05, where only the label of the most significant term per group is shown. The node size represents the term enrichment significance. Node color represents the functional groups.
